# FOLFIRI Plus Durvalumab With or Without Tremelimumab in Second-Line Treatment of Advanced Gastric or Gastroesophageal Junction Adenocarcinoma: The PRODIGE 59-FFCD 1707-DURIGAST Randomized Clinical Trial

**DOI:** 10.1001/jamaoncol.2024.0207

**Published:** 2024-04-04

**Authors:** David Tougeron, Laetitia Dahan, Ludovic Evesque, Karine Le Malicot, Farid El Hajbi, Thomas Aparicio, Olivier Bouché, Nathalie Bonichon Lamichhane, Benoist Chibaudel, Antoine Angelergues, Anaïs Bodere, Jean-Marc Phelip, May Mabro, Laure Kaluzinski, Caroline Petorin, Gilles Breysacher, Yves Rinaldi, Aziz Zaanan, Denis Smith, Marie-Claude Gouttebel, Clément Perret, Nicolas Etchepare, Jean-François Emile, Ivan Sanfourche, Frédéric Di Fiore, Côme Lepage, Pascal Artru, Christophe Louvet

**Affiliations:** 1 Department of Gastroenterology and Hepatology, Poitiers University Hospital, Poitiers, France; 2 Department of Gastroenterology and Hepatology, Marseille University Hospital, Marseille, France; 3 Department of Digestive Oncology, A. Lacassagne Centre, Nice, France; 4 Fédération Francophone de Cancérologie Digestive, EPICAD INSERM LNC-UMR 1231, Bourgogne Franche-Comté University, Dijon, France; 5 Department of Gastroenterology and Digestive Oncology, Oscar Lambret Centre, Lille, France; 6 Department of Gastroenterology and Digestive Oncology, Saint Louis Hospital, Paris, France; 7 Department of Gastroenterology and Digestive Oncology, Reims University Hospital, Reims, France; 8 Department of Gastroenterology, Tivoli Clinic, Bordeaux, France; 9 Department of Oncology, Franco-Britannique Hospital, Levallois, France; 10 Diaconesses Croix Simon Hospital, Paris, France; 11 Saint Malo Hospital, Saint Malo, France; 12 Department of Gastroenterology and Hepatology, Saint Etienne University Hospital, Groupe URCAS, Université Jean Monet, Saint Etienne, France; 13 Department of Oncology, Foch Hospital, Suresnes, France; 14 Department of Oncology, Cherbourg-en-Cotentin Hospital, Cherbourg-en-Cotentin, France; 15 Department of Oncology, Clermont-Ferrand University Hospital, Clermont-Ferrand, France; 16 Department of Gastroenterology and Hepatology, Colmar Hospital, Colmar, France; 17 Department of Gastroenterology, Marseille European Hospital, Marseille, France; 18 Department of Digestive Oncology, Georges Pompidou European Hospital, AP-HP, Université Paris Cité, Paris Cancer Institute CARPEM, Paris, France; 19 Department of Gastroenterology and Hepatology, Bordeaux University Hospital, Bordeaux, France; 20 Department of Gastroenterology, Romans-sur-Isère Hospital, Romans-sur-Isère, France; 21 Department of Oncology, Private Saint-Grégoire Hospital, Saint-Grégoire, France; 22 Department of Gastroenterology, Valence Hospital, Valence, France; 23 Paris-Saclay University, Versailles SQY University, EA4340-BECCOH, Assistance Publique–Hôpitaux de Paris (APHP), Ambroise-Paré Hospital, Pathology Department, Boulogne, France; 24 Department of Pathology, Poitiers University Hospital, Poitiers, France; 25 Department of Hepatogastroenterology, Normandy University, UNIROUEN, Rouen University Hospital, Rouen, France; 26 Department of Gastroenterology, Mermoz Hospital, Lyon, France; 27 Department of Medical Oncology, Institute Mutualiste Montsouris, Paris, France

## Abstract

**Importance:**

Efficacy of second-line chemotherapy in advanced gastric or gastrooesphageal junction (GEJ) adenocarcinoma remains limited.

**Ojectives:**

To determine the efficacy of 1 or 2 immune checkpoint inhibitors combined with FOLFIRI (leucovorin [folinic acid], fluorouracil, and irinotecan) in the treatment of advanced gastric/GEJ adenocarcinoma.

**Design, Setting, and Participants:**

The PRODIGE 59-FFCD 1707-DURIGAST trial is a randomized, multicenter, noncomparative, phase 2 trial, conducted from August 27, 2020, and June 4, 2021, at 37 centers in France that included patients with advanced gastric/GEJ adenocarcinoma who had disease progression after platinum-based first-line chemotherapy.

**Intervention:**

Patients were randomized to receive FOLFIRI plus durvalumab (anti–programmed cell death 1 [PD-L1]) (FD arm) or FOLFIRI plus durvalumab and tremelimumab (anti–cytotoxic T-lymphocyte associated protein 4 [CTLA-4]) (FDT arm). The efficacy analyses used a clinical cutoff date of January 9, 2023.

**Main outcome and Measures:**

The primary end point was progression-free survival (PFS) at 4 months according to RECIST 1.1 criteria evaluated by investigators.

**Results:**

Overall, between August 27, 2020, and June 4, 2021, 96 patients were randomized (48 in each arm). The median age was 59.7 years, 28 patients (30.4%) were women and 49 (53.3%) had GEJ tumors. Four month PFS was 44.7% (90% CI, 32.3-57.7) and 55.6% (90% CI, 42.3-68.3) in the FD and FDT arms, respectively. The primary end point was not met. Median PFS was 3.8 and 5.4 months, objective response rates were 34.7% and 37.7%, and median overall survival was 13.2 and 9.5 months in the FD and FDT arms, respectively. Disease control beyond 1 year was 14.9% in the FD arm and 24.4% in the FDT arm. Grade 3 to 4 treatment-related adverse events were observed in 22 (47.8%) patients in each arm. A combined positive score (CPS) PD-L1 of 5 or higher was observed in 18 tumors (34.0%) and a tumor proportion score (TPS) PD-L1 of 1% or higher in 13 tumors (24.5%). Median PFS according to CPS PD-L1 was similar (3.6 months for PD-L1 CPS ≥5 vs 5.4 months for PD-L1 CPS <5) by contrast for TPS PD-L1 (6.0 months for PD-L1 TPS ≥1% vs 3.8 months for PD-L1 TPS <1%).

**Conclusions and Relevance:**

Combination of immune checkpoint inhibitors with FOLFIRI in second-line treatment for advanced gastric/GEJ adenocarcinoma showed an acceptable safety profile but antitumor activity only in a subgroup of patients.

**Trial Registration:**

ClinicalTrials.gov Identifier: NCT03959293

## Key Points

**Question:**

Are combinations of FOLFIRI (leucovorin [folinic acid], fluorouracil, and irinotecan) plus anti–PD-L1 with or without anti–cytotoxic T-lymphocyte associated protein 4 (CTLA4) in second-line treatment of advanced gastric/gastroesophageal junction (GEJ) adenocarcinoma effective?

**Findings:**

In this phase 2 randomized clinical trial including 96 patients, FOLFIRI plus anti–PD-L1, with or without anti-CTLA4, was associated with an acceptable safety profile. However, the primary end point, progression-free survival at 4 months, was not met despite a subgroup of patients with durable disease control.

**Meaning:**

These findings suggest that for patients with advanced gastric/GEJ adenocarcinoma, immune checkpoint inhibitors plus FOLFIRI should be evaluated in a selected subgroup of patients with favorable biomarkers that remain to be identified.

## Teaser

This randomized clinical trial examines the efficacy of 1 or 2 immune checkpoint inhibitors combined with FOLFIRI in the treatment of advanced gastric/GEJ adenocarcinoma.

## Introduction

The prognosis for advanced gastric and gastroesophageal junction (GEJ) adenocarcinomas remains poor, with overall survival (OS) ranging from 10% to 15% at 5 years.^1^ Until recently, in *ERBB2*-negative unresectable advanced/metastatic tumors, the most frequently used palliative first-line chemotherapy was a doublet of fluoropyrimidine plus a platinum salt.^2^ The addition of docetaxel to platinum/fluoropyrimidine regimens (DCF/TFOX/FLOT) suggest increased OS but with higher toxic effects and is not recommended by most guidelines.^3,4,5^

The first results of anti–programmed cell death 1 (anti-PD1) and anti–programmed cell death-ligand 1 (anti-PD-L1) monoclonal antibodies (mAbs), also called immune checkpoint inhibitors (ICIs), in metastatic gastric/GEJ adenocarcinoma have been negative.^6,7,8^ More recently, the phase 3 CheckMate-649 showed that nivolumab (anti-PD1) plus chemotherapy (XELOX or FOLFOX) was superior to chemotherapy alone in terms of OS and progression-free survival (PFS) in patients with a tumor with a PD-L1 CPS of 5 or higher.^9^ The KEYNOTE-859 also reported positive results of pembrolizumab plus chemotherapy in tumor with PD-L1 CPS of 1 or higher.

Second-line chemotherapies (docetaxel, paclitaxel, irinotecan, or FOLFIRI), compared with best supportive care (BSC) alone, have increased OS.^10,11,12,13^ Currently, the most widely used second-line treatment for gastric/GEJ adenocarcinoma is paclitaxel plus ramucirumab.^14^ The FOLFIRI regimen is also a treatment option in the second-line setting, especially in case of early recurrence after perioperative FLOT chemotherapy and in countries where ramucirumab is not reimbursed. The FOLFIRI regimen provides a median OS and PFS ranging from 4.0 to 9.5 months and 2.5 to 5.3 months, respectively.^11,13,15^

Durvalumab is a mAb directed against PD-L1 and tremelimumab is a mAb against cytotoxic T-lymphocyte associated protein 4 (CTLA-4), and combining these 2 mAbs showed a manageable safety profile.^16^ A recently published phase 1b/2 trial with either durvalumab or tremelimumab alone, or in combination in patients with advanced gastric/GEJ adenocarcinoma demonstrated significant efficacy with a 6-month PFS of 20.0% and a 12-month OS of 38.8% in the durvalumab plus tremelimumab arm.^17^

The PRODIGE 59-FFCD 1707-DURIGAST randomized phase 2 trial aimed to evalute the efficacy and safety of FOLFIRI with durvalumab with or without tremelimumab as the second-line treatment in patients with advanced gastric/GEJ adenocarcinoma.

## Methods

### Study Design

The trial protocol is in Supplement 1 and the statistical analysis plan is in Supplement 2. The PRODIGE 59-FFCD 1707-DURIGAST study was a randomized, open-label, multicenter, noncomparative, phase 2 study conducted at 37 centers in France and designed to evaluate the safety and efficacy of FOLFIRI plus durvalumab (FD arm) and FOLFIRI plus durvalumab and tremelimumab (FDT arm) in patients with advanced gastric/GEJ adenocarcinoma, pretreated with a platinum-based first-line treatment.^18^ This study was sponsored by the Fédération Francophone de Cancérologie Digestive (FFCD).

The PRODIGE 59-FFCD 1707-DURIGAST trial was approved by the French health authorities and an independent ethics committee (Comité de Protection des Personnes Nord-Ouest II, number 2018-002014-13 on April 16, 2019). Written informed consent was obtained from all patients before treatment.

### Patients

The main inclusion criteria were patients aged 18 years or older, histologically proven advanced unresectable (locally advanced or metastatic) gastric/GEJ (Siewert 2 or 3) adenocarcinoma, with progression or intolerance after first-line chemotherapy with fluoropyrimidine plus platinum salt with or without taxane with or without anti-ERBB2 therapies, with an Eastern Cooperative Oncology Group (ECOG) Performance Status (PS) 0 or 1 and adequate organ function. The main exclusion criteria were previous treatment with an ICI and active documented autoimmune or inflammatory disorders (Supplement 3).

Randomization was carried out using the minimisation technique according to a 1:1 ratio to receive FD or FDT and stratified on center and duration of disease control during first-line chemotherapy (no disease control vs <3 months vs ≥3 months).

Patients were evaluated every 8 weeks using clinical examinations, laboratory, and morphological assessments until progression.^18^ Briefly, clinical examinations included ECOG PS and quality of life (QoL) using European Organisation for Research and Treatment of Cancer Core Quality of Life Questionnaire (EORTC QLQ-C30). Morphological assessments were based on thoracic-abdominal-pelvic computed tomographic (CT) scan according to RECIST 1.1 criteria. Adverse events (AEs) were graded according to the National Cancer Institute Common Terminology Criteria for Adverse Events (NCI-CTCAE), version 4.03.

### Treatments

Patients received the FOLFIRI regimen with folinic acid, 400 mg/m^2^, a 5-fluorouracil bolus, 400 mg/m^2^, continuous 5-fluorouracil, 2400 mg/m^2^, and irinotecan at 180 mg/m^2^ every 2 weeks. Durvalumab was administered at a dose of 1500 mg every 4 weeks. Tremelimumab was administered at a dose of 75 mg every 4 weeks. Tremelimumab was administered for only 4 cycles. The treatment was repeated until documented disease progression, unacceptable toxic effects, withdrawal of consent, or patient refusal. In the FDT arm, in cases of progression on FOLFIRI plus durvalumab after previous disease control, tremelimumab could be reintroduced once at the investigator’s discretion.

Due to no data concerning the combination of ICIs plus FOLFIRI, a safety run-in phase was conducted before the randomized phase 2 trial. The results have already been published and have showed an expected safety profile.^19^

### Study Objectives and End Points

The primary end point was the percentage of patients alive and without progression at 4 months (PFS at 4 months) with FD or FDT based on RECIST 1.1 criteria and evaluated by the investigator.

Secondary end points included OS, safety profile, and QoL. Time to strategy failure (TTSF), PFS, objective response rate (ORR), disease control rate (DCR), and duration of response (DoR) were also analyzed. PFS was defined as the time from randomization to first disease progression (according RECIST 1.1 criteria) or death from any cause. Patients alive without progression were censored on the date of last news. TTSF is the time from the treatment start to confirmed progression or death. Pseudoprogression in both arms and reintroduction of tremelimumab at progression in the FDT arm were not considered events to calculate TTSF. Pseudoprogression was defined as unconfirmed disease progression according to iRECIST criteria.^20^ Indeed, at investigator discretion, in case of suspicion of pseudoprogression it is possible to continue the treatment and perform a new CT scan 6 to 12 weeks later to confirm the progression. Disease control beyond 1 year is defined as the percentage of patients with a time from the treatment start to confirmed progression or death after 1 year.

Analyses of tumor biomarkers included expression of DNA mismatch repair (MMR) protein by immunohistochemistry (IHC), microsatellite instability (MSI), and PD-L1 expression. PD-L1 immunohistochemistry was done at a central laboratory using PD-L1 primary antibody (QR-1, 1/100 dilution; Diagomics) to calculate the PD-L1 tumor proportion score (TPS) and combined positive score (CPS).

### Sample Size and Statistical Considerations

Median PFS with FOLFIRI as a second-line chemotherapy in gastric/GEJ adenocarcinoma is between 2 and 4 months.^11,13,15^ Given this and using the binomial exact method to calculate sample size, the hypotheses were H0: 50% of patients alive and without progression at 4 months was not acceptable and H1: 70% of patients alive and without progression at 4 months was expected. With a risk α of 5%, a power of 85% and, according to the binomial exact method, 44 evaluable patients were needed by arm.^21^ If 28 or more patients were alive and without disease progression at 4 months then the arm was considered as efficient. Assuming 5% nonevaluable or lost to follow-up patients, 47 patients were included by arm.

Analyses of primary and secondary efficacy end points were conducted on the modified intention-to-treat (mITT) population, ie, all patients who had received at least 1 dose of treatment in the study. Safety analyses were performed on all patients receiving at least 1 dose of treatment and according to the treatment received (safety population).

Quantitative variables are described with means, medians, standard deviations (SDs), or interquartile ranges (IQRs). Qualitative variables are described as frequencies and percentages. For quantitative variables, baseline characteristics of treatment arms were compared using a *t* test or Wilcoxon test, and for qualitative variables a χ^2^ test or a Fisher exact test was used.

For the primary end point, a 2-sided 90% CI was calculated. Survival criteria were estimated using the Kaplan-Meier method and described by medians and their 95% CIs. Follow-up time was estimated by the reverse Kaplan-Meier method. SAS statistical software (version 9.4; SAS Institute, Inc) was used for all statistical analyses. Efficacy analyses used a clinical cutoff date of January 9, 2023.

## Results

### Patient and Tumor Characteristics

**Figure 1.**
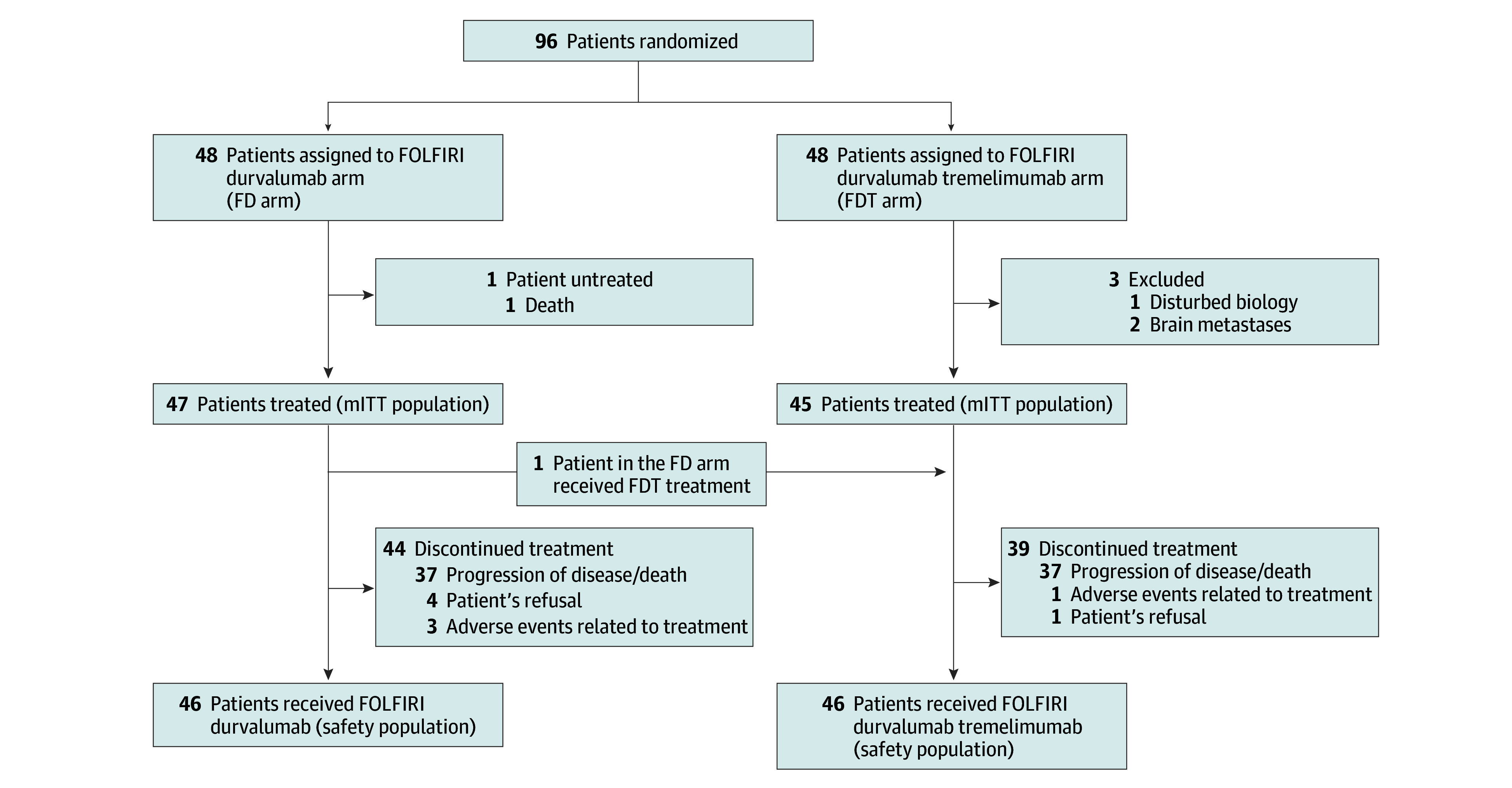
Trial Profile FD arm indicates FOLFIRI (leucovorin [folinic acid], fluorouracil, and irinotecan) plus durvalumab; FDT arm, FOLFIRI plus durvalumab and tremelimumab; mITT, modified intention-to-treat population.

Between August 27, 2020, and June 4, 2021, 96 patients in 37 centers were randomized and 92 patients received 1 or more doses of the treatment (mITT population, 92; 47 in the FD arm and 45 in the FDT arm). One patient randomized in the FD arm received the FDT treatment and was analyzed in the FDT arm for safety analyses (safety population, 92; 46 in each arm) (Figure 1).

The median (SD) age was 59.7 (12.6) years, 28 patients (30.4%) were women and 61 (66.3%) had an ECOG PS of 1 (Table 1). Forty-nine patients had GEJ tumors (53.3%), most with synchronous metastases (60 [65.2%]) and were treated with doublet first-line chemotherapy (56 [60.9%]). Most patients experienced disease control at 3 or more months with the first-line regimen (66 [68.8%]).

**Table 1.** Patient and Tumor Characteristics

Variable	No. (%)		
	All patients (n = 92)	Folfiri plus durvalumab (n = 47)	Folfiri plus durvalumab plus tremelimumab (n = 45)
Age, median (range), y	59.7 (24.7-83.3)	59.3 (28.2-83.3)	60.0 (24.7-82.6)
Sex			
Female	28 (30.4)	14 (29.8)	14 (31.1)
Male	64 (69.6)	33 (70.2)	31 (68.9)
ECOG PS^a^			
0	31 (33.7)	11 (23.4)	20 (44.4)
1	61 (66.3)	36 (76.6)	25 (55.6)
Body mass index, median (range), kg/m^2^	26.3 (16.6-48.4)	26.0 (17.7-39.1)	27.2 (16.6-48.4)
Primary tumor site			
Gastroesophageal junction	49 (53.3)	27 (57.4)	22 (48.9)
Stomach	43 (46.7)	20 (42.6)	23 (51.1)
Tumour subtype (Lauren classification)			
Intestinal type	48 (56.5)	24 (54.5)	24 (58.5)
Diffuse type	37 (43.5)	20 (45.5)	17 (41.5)
Unknown	7	3	4
*ERBB2* status			
Positive	21 (23.1)	11 (23.9)	10 (22.2)
Negative	70 (76.9)	35 (76.1)	35 (77.8)
Unknown	1	1	0
Microsatellite instability			
Deficient	4 (4.5)	3 (6.4)	1 (2.2)
Proficient	85 (92.4)	41 (87.2)	44 (97.8)
Unknown	3	3	0
Time to metastatic disease			
Metachronous	32 (34.8)	17 (36.2)	15 (33.3)
Synchronous	60 (65.2)	30 (63.8)	30 (66.7)
Resection of primary tumor			
No	67 (72.8)	34 (72.3)	33 (73.3)
Yes	25 (27.2)	13 (27.7)	12 (26.7)
Type of disease			
Locally advanced	7 (7.6)	4 (8.5)	3 (6.7)
Metastatic	85 (92.4)	43 (91.5)	42 (93.3)
Site of metastases			
Liver	37 (40.2)	19 (40.4)	18 (40.0)
Lung	18 (19.6)	9 (19.1)	9 (20.0)
Peritoneal carcinomatosis	33 (35.9)	16 (34.0)	17 (37.8)
Lymph nodes	36 (39.1)	19 (40.4)	17 (37.8)
Prior first-line chemotherapy regimen			
Doublet regimen^b^	56 (60.9)	33 (70.2)	23 (51.1)
Triplet regimen^c^	34 (37.0)	13 (27.7)	21 (46.7)
Single agent	2 (2.2)	1 (2.1)	1 (2.2)
CPS PD-L1			
≥5	18 (34.0)	4 (19.0)	14 (43.8)
<5	35 (66.0)	17 (81.0)	18 (56.2)
Unknown	39	26	13
TPS PD-L1			
≥1	13 (24.5)	5 (23.8)	8 (25.0)
<1	40 (75.5)	16 (76.2)	24 (75.0)
Unknown	39	26	13

Abbreviations: CPS, combined positive score; ECOG PS, Eastern Cooperative Oncology Group performance status; PD-L1, programmed cell death-ligand 1; TPS, tumor proportion score.

^a^ Significant difference between 2 groups.

^b^ Fluoropyrimidine plus platinum salt.

^c^ Fluoropyrimidine plus platinum salt plus taxane.

The most frequent metastatic sites were the liver (40.2%), lymph nodes (39.1%) and peritoneum (35.9%). The number of metastatic sites was not different according to treatment arm (patients with 2 or more metastatic sites, 53.5% in FD arm vs 50.0% in FDT arm). Forty-eight tumors (56.5%) were the intestinal type, 4 (4.5%) had deficient MMR and/or MSI status, and 21 (23.1%) were *ERBB2* positive. PD-L1 status was available in 57.6% of the tumors. A PD-L1 CPS of 5 or higher was observed in 34.0% of tumors (19.0% in the FD arm and 43.8% in the FDT arm) and a PD-L1-positive TPS of 1% or higher in 24.5% of tumors (23.8% in the FD arm and 25.0% in the FDT arm).

### Survival and Response Rates

The median follow-up was 20.3 (95% CI, 18.0-22.0) months in the FD arm and 23.2 (95% CI, 17.9-23.5) months in the FDT arm. At the time of the analysis, 44 patients had definitively discontinued FD treatment, and 39 patients had discontinued FDT treatment; the most common reason for treatment discontinuation in both groups was disease progression/death (88.1% in the FD arm and 94.9% in the FDT arm) (Figure 1).

**Figure 2.**
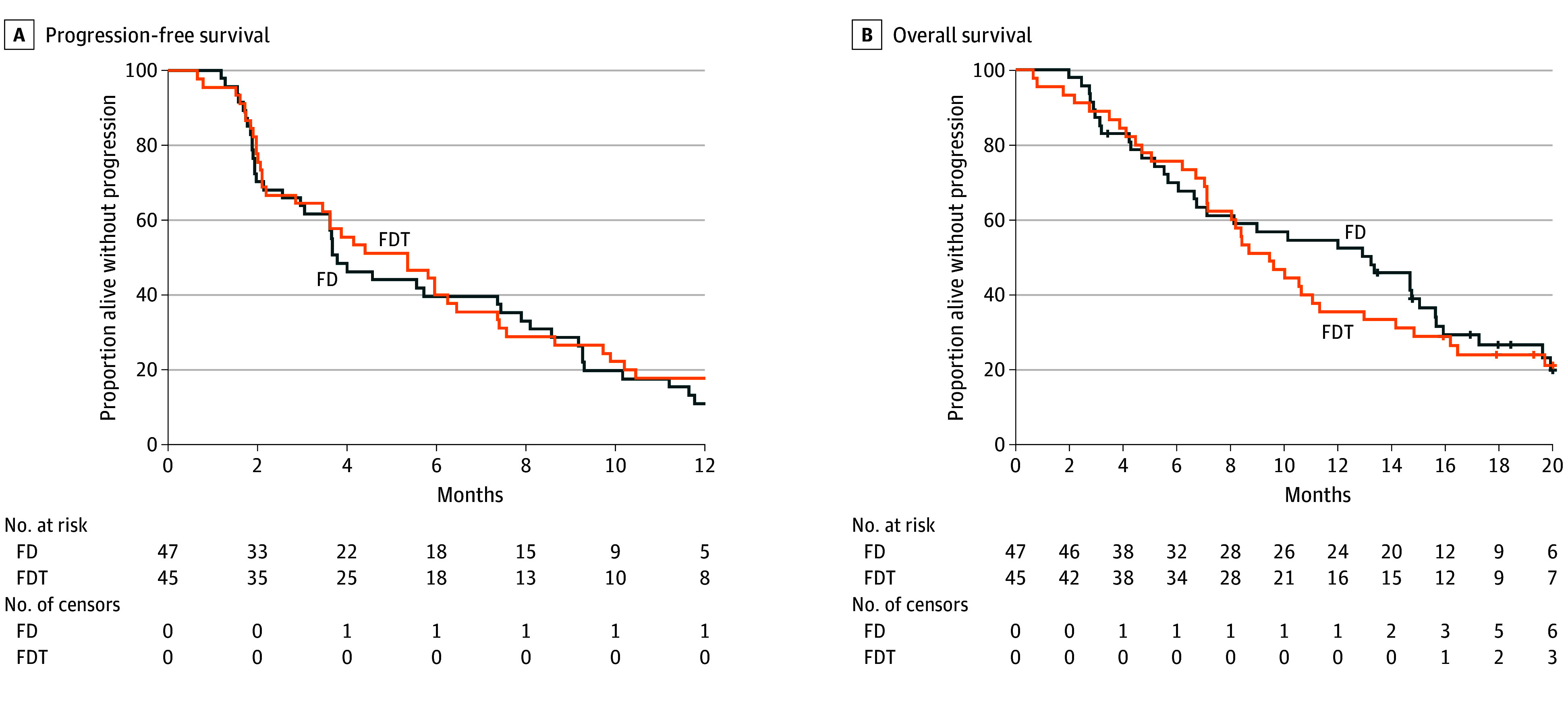
Kaplan-Meier Curves A, There were 44 treatment events in the FD arm and and 41 in the FDT arm. B, There were 36 treatment events in the FD arm and 36 in the FDT arm. FD arm indciates FOLFIRI (leucovorin [folinic acid], fluorouracil, and irinotecan) plus durvalumab; FDT arm, FOLFIRI plus durvalumab and tremelimumab.

At the time of analysis (January 9, 2023), 2 patients (4.3%) in the FD arm and 7 patients (15.2%) in the FDT arm were still under treatment. Median (IQR) duration of treatment was 3.8 (1.5-8.2) and 5.5 (2.3-9.4) months in the FD and FDT arms, respectively. According to RECIST 1.1 criteria, 4-month PFS was 44.7% (90% CI, 32.3%-57.7%) and 55.6% (90% CI 42.3%-68.3%) in the FD and FDT arms, respectively. The primary end point was not met, whatever the treatment arm. Median PFS was 3.8 (95% CI, 3.0-7.4) and 5.4 (95% CI 2.9-6.4) months in the FD and FDT arms, respectively (Figure 2A). Twelve-month PFS was 11.0% (95% CI, 4.0%-21.9%) and 17.8% (95% CI, 8.3%-30.1%) in the FD and FDT arms, respectively.

Overall, ORR (16 patients [34.7%] in the FD arm and 17 patients [37.7%] in the FDT arm) and DCR (31 patients [67.4%] and 31 patients [68.9%]) were similar in both arms. Median DoR was 6.1 months in the FD arm and 10.0 months in the FDT arm. Disease control beyond 1 year was 14.9% in the FD arm and 24.4% in the FDT arm. Pseudo-progression was observed in 2 patients in the FD arm and 3 patients in the FDT arm. Tremelimumab was reintroduced in 9 patients in the FDT arm after progression but none showed disease control after this reintroduction. Median TTSF was 4.9 months in the FD arm and 6.0 months in the FDT arm. Among patients who stopped the experimental treatment, we observed a similar rate of third-line treatment in the 2 arms (59.1% in the FD arm vs 59.0% in the FDT arm).

Most patients died (36 patients [76.6%] in the FD arm and 35 patients [77.8%] in the FDT arm). Median OS was 13.2 (95% CI, 6.6-15.6) and 9.5 (95% CI, 7.1-11.3) months in the FD and FDT arms, respectively (Figure 2B). At 12 months it was 52.4% (95% CI, 37.2-65.6) in the FD arm and 35.6% (95% CI, 22.0-49.3) in FDT arm, respectively.

### Predictive Factors of Treatment Efficacy

In the overall population, median PFS according to PD-L1 CPS was 3.6 (95% CI, 1.9-5.9) months for PD-L1 CPS of 5 or higher vs 5.4 months (95% CI, 3.6-7.4) for PD-L1 CPS lower than 5 (eFigure 1 in Supplement 3). Twelve-month PFS was 16.7% (95% CI, 4.1-36.5) and 8.6% (95% CI, 2.2-20.6), respectively. Median PFS according to PD-L1 TPS tended to be higher in tumors with PD-L1 TPS of 1% or higher than in tumors with PD-L1 TPS lower than 1% (6.0 months [95% CI, 2.0-7.4] vs 3.8 months [95% CI, 2.9-5.6]) (eFigure 2 in Supplement 3). Twelve-month PFS was 15.4% (95% CI, 2.5-38.8) and 10.0% (95% CI, 3.2-21.5), respectively.

For tumors with PD-L1 CPS of 5 or higher, median PFS and 12-month PFS was 1.9 months (95% CI, 1.7-NA) and 0% vs 5.0 months (95% CI, 1.9-7.4) and 21.4% (95% CI, 5.2%-44.8%) for the FD and FDT arms, respectively. For tumors with PD-L1 TPS of 1% or higher, median PFS and 12-month PFS was 3.6 months (95% CI, 1.8-7.9) and 0% vs 6.2 months (95% CI, 1.7-NR) and 25.0% (95% CI, 3.7%-55.8%) in the FD and FDT arms, respectively.

In univariate and multivariable analyses, only the presence of liver metastases was associated with worse PFS (eTable in Supplement 3). For patients with and without liver metastases, median PFS and 12-month PFS was 3.1 months (95% CI, 2.0-4.1) and 2.9% (95% CI, 0.2%-12.7%) vs 5.9 months (95% CI, 3.6-9.2) and 21.8% (95% CI, 12.1%-33.4%), respectively.

### Safety and Quality of Life

Most patients had at least 1 treatment-related AE (43 [93.5%] in the FD arm and 43 [93.5%] in the FDT arm). Grade 3 to 4 treatment-related AEs were observed in 22 patients (47.8%) in both arms (asthenia, 8 [17.4%] vs 13 [28.3%]; neutropenia, 7 [15.2%] vs 11 [23.9%]; anemia, 5 [10.9%] vs 3 [6.5%]; diarrhea, 1 [2.2%] vs 5 [10.9%]; and vomiting, 3 [6.5%] vs 3 [6.5%] in the FD and FDT arms, respectively) (Table 2). Immune-related grade 3 to 4 AEs were observed in 4 patients (8.7%) in the FD arm and 5 patients (10.9%) in the FDT arm and were mostly diarrhea/colitis (5 [5.4%]). No death was considered treatment related.

**Table 2.** Treatment-Related Adverse Events^a^

Event^b^	No. (%)			
	Folfiri plus durvalumab (n = 46)		Folfiri plus durvalumab plus tremelimumab (n = 46)	
	Grades 1-2	Grades 3-4-5	Grades 1-2	Grades 3-4-5
Patients with ≥1 adverse event	43 (93.5)	22 (47.8)	43 (93.5%)	22 (47.8%)
Nausea	29 (63.0)	2 (4.3)	22 (47.8)	5 (10.9)
Fatigue	26 (56.5)	8 (17.4)	23 (50.0)	13 (28.3)
Diarrhea	23 (50.0)	1 (2.2)	30 (65.2)	5 (10.9)
Anaemia	20 (43.5)	5 (10.9)	30 (65.2)	3 (6.5)
Neutrophil count decrease	14 (30.4)	7 (15.2)	10 (21.7)	11 (23.9)
Vomiting	12 (26.1)	3 (6.5)	12 (26.1)	3 (6.5)
Anorexia	11 (23.9)	1 (2.2)	15 (32.6)	2 (4.3)
Stomatitis	11 (23.9)	0	19 (41.3)	1 (2.2)
Lymphocyte count decrease	10 (21.7)	2 (4.3)	13 (28.3)	3 (6.5)
Platelet count decrease	9 (19.6)	0	10 (21.7)	0
Alopecia	7 (15.2)	0	12 (26.1)	0
AST increase	6 (13.0)	0	6 (13.0)	0
ALT increase	6 (13.0)	0	4 (8.7)	0
Pruritus	5 (10.9)	0	9 (19.6)	0
PPES	5 (10.9)	0	7 (15.2)	0
Rash	3 (6.5)	0	5 (10.9)	0
Hypothyroidism	3 (6.5)	0	6 (13.0)	0
Hyperthyroidism	1 (2.2)	0	8 (17.4)	**0**
Colitis	0	2 (4.3)	0	0

Abbreviations: ALT, alanine aminotransferase; AST, aspartate aminotransferase; PPES, palmar-plantar erythrodysesthesia syndrome.

^a^ Only adverse events in 10% or more of treated patients were reported as well as immune-related adverse events.

^b^ The total of adverse events could be superior to the total number of patients because some patients could have more than 1 adverse event.

Four patients (8.7%) in the FD arm and 3 patients (6.5%) in the FDT arm definitively stopped treatment due to treatment-related AEs. One patient (2.2%) in each arm definitively stopped durvalumab due to immune-related AEs. Two patients (4.3%) in the FDT arm definitively stopped tremelimumab due to immune-related AEs.

Median time until deterioration in QoL (loss of more than 10 points in the EORTC QLQC30 score) was 7.4 (95% CI, 4.2-12.0) months in the FD arm and 8.3 (95% CI, 4.7-14.8) months in the FDT arm (eFigure 3 in Supplement 3).

## Discussion

To our knowledge, PRODIGE 59-FFCD 1707-DURIGAST is the first trial to evaluate FOLFIRI plus ICI for patients with gastric/GEJ adenocarcinoma, for which treatment options in second-line settings are limited. The primary end point was not met because the 90% CI of PFS at 4 months did not include 70%. Four-month PFS was 44.7% (90% CI, 32.3%-57.7%) and 55.6% (90% CI, 42.3%-68.3%) in the FD and FDT arm, respectively. The primary end point was perhaps a too early end point because it did not take into account patients with long disease control. When we designed the study, we did not know that long-term disease control would be a more relevant end point to evaluate ICI efficacy. Indeed, we observed remarkable disease control beyond 1 year in about 20% of patients compared with less than 10% for chemotherapy with or without targeted therapy.^11,13,14,15^ In the same way, the median duration of response was 6.1 months in the FD arm and 10.0 months in the FDT arm as compared with 4.4 months with paclitaxel plus ramucirumab or about 3 months with FOLFIRI.^11,12,13,14,15^

Median OS was about 12 months (13.2 months in the FD arm and 9.5 months in the FDT arm); to our knowledge, this has never been reached in any other second-line trials. The best OS to date was 9.6 months obtained with ramucirumab combined with paclitaxel.^11,13,14,15^ OS results need to be interpreted with caution in comparisons with other trials because OS was a secondary end point and not used to calculate the number of patients. In addition, new treatments, such as trifluridine/tipiracil, were included in later lines of therapy for advanced gastric/GEJ adenocarcinoma, which could also explain the high OS.

Many data suggest that anti-PD1/anti–PD-L1 efficacy depends on PD-L1 CPS in advanced gastric/GEJ adenocarcinoma.^8,9^ In PRODIGE 59-FFCD 1707-DURIGAST, PFS did not seem to vary according to PD-L1 CPS. By contrast, there was a trend toward better PFS for tumors with PD-L1 TPS of 1% or higher. These results need to be interpreted with caution because PD-L1 expression was available in only 57.6% of tumors. Moreover, the number of patients was too small to analyze PFS in the subgroup of difficient MMR/MSI or *ERBB2*-positive tumors.

FD and FDT combinations had an acceptable safety profile, with 47.8% of treatment-related grade 3 to 4 AEs. The most frequent grade 3 to 4 AEs were asthenia (21 [22.8%]), neutropenia (18 [19.6%]), anemia (8 [8.7%]), and diarrhea (6 [6.5%]) in accordance with grade 3 to 4 AE rates for an irinotecan-based regimen.^11,12,15^ It is known that combining anti–PD-L1/anti–PD-1 with anti–CTLA-4 increases the proportion of grade 3 to 4 immune-related AEs from 10% to 55%, which is higher than the rate observed in our study of 10.9%.^17,22^ The fact that we used only 4 courses of anti–CTLA-4 probably lowered the proportion of immune-related AEs. The median time to a deterioration in QoL was similar in both arms (7-8 months) and close to previously published studies in first-line settings.^23,24^

### Limitations

The main limitation of the PRODIGE 59-FFCD 1707-DURIGAST trial is that no patients received nivolumab combined with oxaliplatin-based chemotherapy in the first-line setting, which is now the standard of care for tumors with PD-L1 CPS of 5 or higher.^9^ Another limitation is the absence of a control arm with FOLFIRI alone. Currently, the most widely used standard second-line treatment for gastric/GEJ adenocarcinoma is paclitaxel plus ramucirumab but an irinotecan-based regimen is also an option, especially in patients with early recurrence or progression during/after a perioperative FLOT regimen.^5,14^

## Conclusions

PRODIGE 59-FFCD 1707-DURIGAST showed that FOLFIRI combined with ICIs has an acceptable safety profile and provides significant antitumor activity in a subgroup of about 20% of patients, even if the primary end point was not met. This regimen thus deserves evaluation in a randomized phase 3 clinical trial comparing FOLFIRI combined with ICIs vs FOLFIRI alone in a selected subgroup of patients with favorable biomarkers that remain to be identified. This combination should be evaluated in second-line settings not only after oxaliplatin-based chemotherapy alone or combined with ICIs in the first-line setting, but also in cases of early recurrence or progression during perioperative FLOT combinations.

## Notes

Supplement 1. Trial Protocol

Supplement 2. Statistical Analysis Plan

Supplement 3. eTable 1. Univariate and multivariate analysis of predictive of factors of progression-free survival eFigure 1. Progression-free survival according to combined positive score (CPS) eFigure 2. Progression-free survival according to tumor proportion score (TPS) eFigure 3. Time to deterioration (10 points) in global quality of life score

Supplement 4. The PRODIGE 59-FFCD 1707-DURIGAST investigators/collaborators

Supplement 5. Data Sharing Statement
