# Supplementary material for: FOLFIRI Plus Durvalumab With or Without Tremelimumab in Second-Line Treatment of Advanced Gastric or Gastroesophageal Junction Adenocarcinoma: The PRODIGE 59-FFCD 1707-DURIGAST Randomized Clinical Trial
Source: JAMA Oncol. 2024 Apr 4;10(6):709–17. doi: 10.1001/jamaoncol.2024.0207 (PMC11190792; doi:10.1001/jamaoncol.2024.0207)
Supplement: Supplement 1. — Trial Protocol [file jamaoncol-e240207-s001.pdf]

PRODIGE 59 - (FFCD 1707) – DURIGAST
A randomized phase II study evaluating FOLFIRI + durvalumab vs FOLFIRI + durvalumab and tremelimumab in second-line treatment of patients with advanced gastric or gastro-oesophageal junction adenocarcinoma

Randomized – non-comparative – multicenter phase II

N° EudraCT : 2018-002014-13

An intergroup trial: FFCD – UNICANCER-GI – GERCOR

Principal investigator (FFCD):
Pr. David Tougeron
CHU de Poitiers
Service d'Hépato-gastro-entérologie
2 Rue de la Milétrie
86021 POITIERS
FRANCE
Phone : +33 (0)5 49 44 37 51
Fax : +33 (0)5 49 44 48 60
Email : david.tougeron@chu-poitiers.fr

Co-principal investigator (FFCD):
Pr. Frédéric Di Fiore
CHU Charles Nicolle
Service d'Oncologie
1 Rue Germont
76031 ROUEN
FRANCE
Phone : + 33 (0)2 32 88 86 10
Fax : +33 (0)2 32 88 86 69
Email : frederic.di-fiore@chu-rouen.fr

Co-principal investigator (GERCOR):
Pr. Christophe Louvet
Institut Mutualiste Montsouris
42 Boulevard Jourdan
75014 PARIS
FRANCE
Phone : + 33 (0)1 56 61 60 35
Fax : +33 (0)1 56 61 69 60
Email : christophe.louvet@imm.fr

Co-principal investigator (UNICANCER GI):
Dr. Farid El Hajbi
Centre Oscar Lambret
Département d'oncologie générale
3 Rue Frédéric Combemale
BP 307- 59020 Lille CEDEX
FRANCE
Phone : + 33 (0)3 20 29 59 42
Fax : +33 (0)3 20 29 55 00
Email : f-elhajbi@o-lambret.fr

COMMITTEE OF REDACTION :
David Tougeron (Poitiers, CHU), Frédéric Di Fiore (Rouen, CHU), Karine le Malicot (Dijon, FFCD), Pierre Laurent-Puig (Paris HEGP), Frédéric Bibeau (Caen, CHU), Christophe Louvet (Paris, Montsouris), Farid El Hajbi (Lille, CAC), Thierry Lecomte (Tours, CHU), Thomas Aparicio (Paris, Saint-Louis), Astrid Lièvre (Rennes, CHU), Rosine Guimbaud (Toulouse, CHU), Stefano Kim (Besançon, CHU), Emilie Barbier (Dijon, FFCD), Sabrina Pierre (Dijon, FFCD), Daniel Gonzalez (Dijon, FFCD)

BIOLOGICAL COMMITTEE :
Pierre-Laurent Puig (Paris), David Tougeron (Poitiers), Frédéric Di Fiore (Rouen), Frédéric Bibeau (Caen), Thierry Lecomte (Tours), Thomas Aparicio (Paris), Astrid Lièvre (Rennes), Harry Sokol (Paris)

SPONSOR AND DATA CENTER :
Fédération Francophone de Cancérologie Digestive (FFCD) - Faculté de Médecine - 7, Boulevard Jeanne d'Arc - BP 87900 - 21079 Dijon Cedex - France

Executive director: **Cécile GIRAULT**
Tél. : +33 (0)3 80 66 80 13 – Fax : +33 (0)3 80 38 18 41
Courriel : cecile.girault@u-bourgogne.fr

Statistician: **Karine LE MALICOT**
Tél. : +33 (0)3 80 39 34 71– Fax : +33 (0)3 80 38 18 41
Courriel : karine.le-malicot@u-bourgogne.fr

Project manager: **Daniel GONZALEZ**
Tél. : +33 (0)3 80 39 34 04 – Fax : +33 (0)3 80 38 18 41
Courriel : daniel.gonzalez@u-bourgogne.fr

Confidential
This document is the property of the Fédération Francophone de Cancérologie Digestive and may not be transferred, reproduced, published or used, in whole or in part, without the federation's express authorization.

TABLE OF CONTENTS

1. STUDY OBJECTIVE 14
1.1. Safety run-in phases objectives 14
1.3. Secondary objectives for phase II study 14
2. PATIENT SELECTION ON REGISTRATION/RANDOMIZATION 14
2.1. Inclusion criteria 14
2.2. Non-inclusion criteria 15
3. INCLUSION ASSESSMENT 16
4. REGISTRATION/RANDOMIZATION 17
5. STUDY DESIGN 18
5.1. Safety run-in 18
5.2. Phase II study 18
6. TREATMENTS 19
6.1. Description, packaging and labeling of durvalumab 19
6.2. Description, packaging and labeling of tremelimumab 20
6.3. FOLFIRI + durvalumab (1st and 2nd steps of the safety run-in phase and Arm A) 21
6.4. FOLFIRI + durvalumab + tremelimumab (2nd step of the safety run-in phase and Arm B) 21
7. DOSE ADJUSTMENT BASED ON TOXICITY 23
7.1. Criteria that must be met before each subsequent cycle (D1 of each cycle) 23
7.2. Dose adjustment based on toxicities observed during the rest period 23
7.3. Dose adjustment based on infusion-related reaction 24
7.4. Dose adjustment of FOLFIRI 24
7.5. Management of Immune-mediated adverse event (imAE) 26
7.6. Partial treatments stop 35
7.7. Premedications, concomitant treatments and contraindicated treatments 36
7.7.1. Neutropenia 36
7.7.2. Contraindicated treatments (see SmPCs and IB of each protocol’s molecular entities) 36
8. LOGISTICS OF THE BIOLOGICAL STUDY (FOR PHASE II ONLY) 36
9. PATIENT MONITORING 37
9.1. During treatment 37
9.1.1. Before each administration of treatment 37
9.1.2. Evaluation every 8 weeks until radiological progression 38
9.2. After treatment discontinuation 38
9.2.1. Within 30 days for evaluating the toxicity of the last treatment 38
9.2.2. After premature discontinuation of treatment other than for radiological progression* 38
9.2.3. After treatment discontinuation because of radiological progression 39
10. SUBSEQUENT TREATMENTS 39
11. MANAGEMENT OF SERIOUS ADVERSE EVENTS 39
12. STATISTICAL ANALYSIS 42
12.1. Safety analyses (safety run-in phase) 42
12.2. Endpoints for phase II study 42
12.2.1. Primary efficacy endpoint 42
12.2.2. Secondary endpoints 42
12.3. Sample size justification, statistical hypothesis 43
12.4. Statistical analysis for phase II study 44
12.4.1. Population definition 44
12.4.2. Endpoint evaluation 44
13. STUDY COMMITTEES 45
13.1. Independent data monitoring committee 45
13.2. Steering committee 45
13.3. Medical review 45
13.4. Biological research committee 45
14. BACKGROUND INFORMATION AND RATIONALE FOR THE TRIAL 45
15. REFERENCES 47
16. ADMINISTRATIVE CONSIDERATIONS 49
17. RULES FOR PUBLICATION 50
18. APPENDICES 50
APPENDIX 1: CLINICAL AND BIOLOGICAL INFORMED CONSENT 51
APPENDIX 2: BIOLOGICAL STUDIES 52

APPENDIX 3: QUALITY OF LIFE – QLQ-C30 .... **55**
APPENDIX 4: QUALITY OF LIFE - STO-22 .... **56**
APPENDIX 5: ECOG PERFORMANCE STATUS – CALCULATION OF CLEARANCE **57**
APPENDIX 6 : RECIST CRITERIA, VERSION 1.1 .... **58**
APPENDIX 7: IRECIST CRITERIA OF IMMUNOLOGICAL RESPONSE **60**
APPENDIX 8: ASSESSMENT OF TOXICITIES **64**
APPENDIX 9: SUMMARY OF CHARACTERISTICS PRODUCT AND INVESTIVATOR'S BROCHURE **88**
APPENDIX 10: AUTHORIZED AND PROHIBITED CONCOMITTANT MEDICATIONS **89**
APPENDIX 11: SERIOUS ADVERSE EVENT REPORT FORM **91**
APPENDIX 12: RULES FOR PUBLICATION FOR PRODIGE TRIALS **95**
APPENDIX 13: INSURANCE CERTIFICATE **97**
APPENDIX 14: APPROVAL OF THE IRB **98**
APPENDIX 15: ANSM AUTHORIZATION **99**

LIST OF ABBREVIATIONS

Abbreviation or special term	Explanation
AChE	Acetylcholine esterase
ADA	Anti-drug antibody
AE	Adverse event
AESI	Adverse event of special interest
ALK	Anaplastic lymphoma kinase
ALT	Alanine aminotransferase
ANC	Absolute neutrophil count
APF12	Proportion of patients alive and progression free at 12 months from randomization
AST	Aspartate aminotransferase
AUC	Area under the curve
$AUC_{0\text{-}28day}$	Area under the plasma drug concentration-time curve from time zero to Day 28 post-dose
AUC_{ss}	Area under the plasma drug concentration-time curve at steady state
BICR	Blinded Independent Central Review
BoR	Best objective response
BP	Blood pressure
C	Cycle
CBC	Complete blood count
CD	Cluster of differentiation
CI	Confidence interval
CL	Clearance
C_{max}	Maximum plasma concentration
$C_{max,ss}$	Maximum plasma concentration at steady state
CR	Complete response
CSA	Clinical study agreement
CSR	Clinical study report
CT	Computed tomography
CTCAE	Common Terminology Criteria for Adverse Event
CTLA-4	Cytotoxic T–lymphocyte-associated antigen 4
$C_{trough,ss}$	Trough concentration at steady state
CXCL	Chemokine (C-X-C motif) ligand
DoR	Duration of response
EC	Ethics Committee, synonymous to Institutional Review Board and Independent Ethics Committee
ECG	Electrocardiogram
ECOG	Eastern Cooperative Oncology Group
eCRF	Electronic case report form
EDoR	Expected duration of response
EGFR	Epidermal growth factor receptor
EU	European Union
FAS	Full analysis set
FDA	Food and Drug Administration
GCP	Good Clinical Practice
GI	Gastrointestinal
GMP	Good Manufacturing Practice
hCG	Human chorionic gonadotropin
HIV	Human immunodeficiency virus
HR	Hazard ratio
IB	Investigator's Brochure
ICF	Informed consent form
ICH	International Conference on Harmonization
IDMC	Independent Data Monitoring Committee
IFN	Interferon
IgE	Immunoglobulin E
IgG	Immunoglobulin G
IHC	Immunohistochemistry
IL	Interleukin
ILS	Interstitial lung disease
IM	Intramuscular

IMT	Immunomodulatory therapy
IP	Investigational product
irAE	Immune-related adverse event
IRB	Institutional Review Board
iRECIST	Immune-related Response Evaluation Criteria in Solid Tumors
ITT	Intent-to-Treat
IV	Intravenous
IVRS	Interactive Voice Response System
IWRS	Interactive Web Response System
LFT	Liver function tests
mAb	Monoclonal antibody
MDSC	Myeloid-derived suppressor cell
MedDRA	Medical Dictionary for Regulatory Activities
MHLW	Minister of Health, Labor, and Welfare
miRNA	Micro-ribonucleic acid
MRI	Magnetic resonance imaging
NCI	National Cancer Institute
NE	Not evaluable
NSCLC	Non–small-cell lung cancer
OAE	Other significant adverse event
ORR	Objective response rate
OS	Overall survival
PBMC	Peripheral blood mononuclear cell
PD	Progressive disease
PD-1	Programmed cell death 1
PD-L1	Programmed cell death ligand 1
PD-L2	Programmed cell death ligand 2
PDx	Pharmacodynamic(s)
PFS	Progression-free survival
PFS2	Time to second progression
PGx	Pharmacogenetic research
PJP	Pneumocystis jiroveci Pneumonia
PK	Pharmacokinetic(s)
PR	Partial response
QTcF	QT interval corrected for heart rate using Fridericia's formula
RECIST 1.1	Response Evaluation Criteria in Solid Tumors, version 1.1
RNA	Ribonucleic acid
RR	Response rate
RT-QPCR	Reverse transcription quantitative polymerase chain reaction
SAE	Serious adverse event
SAP	Statistical analysis plan
SAS	Safety analysis set
SD	Stable disease
SNP	Single nucleotide polymorphism
SoC	Standard of Care
sPD-L1	Soluble programmed cell death ligand 1
T_3	Triiodothyronine
T_4	Thyroxine
TSH	Thyroid-stimulating hormone
TNF	Tumor necrosis factor
ULN	Upper limit of normal
US	United States
WBDC	Web-Based Data Capture
WHO	World Health Organization

FFCD CONTACTS FOR THE STUDY

NAME	FUNCTION	TEL (+33)	FAX (+33)	EMAIL
**FFCD DATA CENTER**				
Cécile GIRAULT	Executive Director	(0)3 80 39 33 87	(0)3 80 38 18 41	cecile.girault@u-bourgogne.fr
Marie MOREAU	Clinical Operation leader	(0)3 80 39 34 04	(0)3 80 38 18 41	marie.moreau@u-bourgogne.fr
**Operational team**				
Daniel GONZALEZ	Project manager	(0)3 80 39 34 04	(0)3 80 38 18 41	daniel.gonzalez@u-bourgogne.fr
Caroline CHOINE-POURET	CRA coordinator	(0)4 69 18 19 02	(0)9 74 44 22 47	caroline.choine@u-bourgogne.fr
Coralie DEVORSINE	Assistant project manager	(0)3 80 39 34 86	(0)3 80 38 18 41	coralie.devorsine@u-bourgogne.fr
Carole MONTERYMARD	Data manager	(0)3 80 39 34 84	(0)3 80 38 18 41	carole.monterymard@u-bourgogne.fr
Karine Le MALICOT	Biostatistician	(0)3 80 39 34 71	(0)3 80 38 18 41	Karine.Le-Malicot@u-bourgogne.fr
Sabrina PIERRE	Pharmacovigilance manager	(0)7 76 00 09 72	(0)3 80 38 18 41	sabrina.pierre@u-bourgogne.fr

PRODIGE 59 - (FFCD 1707) – DURIGAST
A randomized phase II study evaluating FOLFIRI + durvalumab vs FOLFIRI + durvalumab and tremelimumab in second-line treatment of patients with advanced gastric or gastro-oesophageal junction adenocarcinoma
EudraCT no. 2018-002014-13

Version 1.0–08/11/2018

This version of the protocol has been approved by:
The sponsor: Cécile Girault Date: 08.11.2018 Signature:
The coordinator: Prof. David Tougeron Date: 08.11.2018 Signature:

I the undersigned, Dr./Pr………. having taken note of the pre-requisites of this research and of the protocol and its appendices, certify that I will undertake to conduct this trial in compliance with Good Clinical Practice and in accordance with the applicable provisions of the French Public Health Code.

I particularly undertake to:
- comply with the protocol as well as any amendments of which the sponsor notifies me
- supervise the research in the center, train my colleagues in conducting the research, and provide a list of the names of my colleagues
- obtain the status of patients from the vital records office at the time of analysis or if the sponsor so requests if patients are lost to follow-up
- have each patient sign a written consent form once I have familiarized the patient with the information sheet. This I undertake to do before any procedure is performed for the research
- report any serious adverse events or new facts within 24 hours of their being brought to my attention in accordance with the protocol's instructions
- comply with the inclusion and non-inclusion criteria as well as with the start and end dates of the trial
- participate in the biological section of the trial and dispatch the samples according to the guidelines
- fill in all items of the CRF and ensure that data collection is up to standard and that the products are properly managed
- retain the data and documents relating to the trial for 15 years after the trial has ended
- inform the sponsor of any conflict of interest that may damage my scientific independence within the framework of the research
- immediately inform the sponsor of any legal action, whether amicable or contentious, brought by a person participating in the research or by that person's assignees in which the sponsor may be held accountable
- accept periodic visits from the sponsor's representatives and make all source documents and materials relating to the research available to them so that they may verify the quality of data recorded in the CRF. Accept audits by the sponsor or one of its representatives and/or inspections by the health authorities
- reply by phone or email to requests for corrections or clarifications concerning the CRF
- accord the FFCD CRA the time necessary for signing forms, answering any questions and carrying out corrective actions

Date: Signature:

CENTER'S STAMP:

Send the original to the FFCD Randomization, Management and Analysis Center – 7 bd Jeanne d'Arc – BP 87900 – 21079 Dijon Cedex, France

SYNOPSIS

**Title**	**PRODIGE 59 - (FFCD 1707) – DURIGAST**<br>**A randomized phase II study evaluating FOLFIRI + durvalumab vs FOLFIRI + durvalumab and tremelimumab in second-line treatment of patients with advanced gastric or gastro-oesophageal junction adenocarcinoma**
**Sponsor**	French Federation of Digestive Oncology (FFCD)
**Design**	**Safety Run-In** and multicenter randomized phase II non-comparative open-label study
**Study objectives**	**Safety Run-In phase:**<br>Validate the good tolerability of treatments combination in 2 steps:<br>- 1st step : treated 5 patients with FOLFIRI (irinotecan at 180 mg/m²) + durvalumab<br>- 2nd step: randomized 6 patients between FOLFIRI (irinotecan at 180 mg/m²) + durvalumab versus FOLFIRI (**irinotecan at 150 mg/m²**) + durvalumab + tremelimumab (3 patients per arm)<br>Patients will be treated in 5 expert centers with a huge experience in the use of immune checkpoints inhibitors. Inclusions will be stopped at each step for safety analyses.<br>**Phase II study:**<br>**Primary objective:**<br>- Percentage of patients alive and without progression at 4 months of FOLFIRI plus durvalumab versus FOLFIRI plus durvalumab plus tremelimumab in patients with advanced-stage gastric or gastro-oesophageal junction adenocarcinoma and who progressed after a first line chemotherapy (based on RECIST 1.1 rating scale evaluated by the investigator).<br>**Secondary objectives:**<br>- Percentage of patients alive and without progression at 4 months according to centralized review<br>- Overall survival (OS)<br>- Time to strategy failure<br>- Safety profile<br>- Quality of life (QoL)<br>- Time to progression (TTP), progression-free survival (median PFS), best objective response rate (BRR) and disease control rate (DCR) according to the investigator and centralized review (according RECIST V1.1 and iRECIST criteria)<br>- Efficacy endpoints (OS, PFS, TTP, BRR and DCR) according to the expression of PD-L1 and others biomarkers (see biological study)
**Inclusion criteria**	- Age ≥ 18 years.<br>- Body weight > 30kg.<br>- Histologically proven advanced-stage unresectable adenocarcinoma of the stomach or the GEJ (Siewert II or III).<br>- Known MSS/MSI status or tumor tissue available (frozen or paraffin-embedded, primary tumors or metastases) in order to allow determination of MSS/MSI status. The investigator needs to ensure that tumor tissues will be sent after patient randomization.<br>- Failure to platinium-based 1st line therapy with or without trastuzumab, or early recurrent disease after surgery with neo-adjuvant and/or adjuvant platinium-based chemotherapy (within 6 months of the end of chemotherapy) or progression during neo-adjuvant and/or adjuvant platinium-based chemotherapy.<br>- Eligible for a second-line treatment with irinotecan and 5-FU.<br>- Measurable or non-measurable lesion according to the Response Evaluation Criteria in Solid Tumors (RECIST 1.1).<br>- Eastern Cooperative Oncology Group (ECOG) performance status 0-1.<br>- Adequate organ function: ANC ≥ 1.5 x 10^9/L, haemoglobin ≥ 9 g/dL, platelets ≥ 100 x 10^9/L, AST/ALT ≤ 3 x ULN (≤ 5 x ULN in case of liver metastase(s)), GGT ≤ 3 x ULN (≤ 5 x ULN in case of liver metastase(s)), bilirubin ≤ 1.5 x ULN, creatinin clearance > 40 mL/min (MDRD).<br>- Evidence of post-menopausal status or negative urinary or serum pregnancy test for female pre-menopausal patients.<br>- Man and woman who childbearing potential agrees to use two methods (one for the patient and one for the partner) of medically acceptable forms of contraception during the study and for 6 months after the last treatment intake.<br>- Patient is able to understand, sign, and date the written informed consent form at the screening visit prior to any protocol-specific procedures performed.

**Non-inclusion criteria**	- Concurrent enrolment in another clinical study – unless it is an observational study or during the follow-up period of an interventional study.<br>- Receipt of the last dose of anticancer therapy ≤ 2 weeks prior to the first dose of study drug.<br>- Radiotherapy within 4 weeks prior to the first dose of treatment.<br>- History of chronic inflammatory bowel disease (IBD).<br>- Current or prior bowel obstruction within 28 days before the first dose of study drugs.<br>- Any unresolved significant toxicity NCI CTCAE v4.0 ≥ grade 2 from previous anticancer therapy.<br>- Concurrent use of hormonal therapy for non–cancer-related conditions is acceptable<br>- Major surgical procedure (e.g. exploratory laparoscopy is not considered as a major surgical procedure) within 28 days prior to the first dose of treatment.<br>- Prior allogeneic bone marrow transplantation or prior solid organ transplantation.<br>- Active or prior documented autoimmune or inflammatory disorders (patients with alopecia, vitiligo, controlled hypo or hyperthyroidism, any chronic skin condition not requiring immunosuppressant therapy are eligible). Patients without active disease in the last 5 years may be included.<br>- Uncontrolled intercurrent illness, including but not limited to, ongoing or active infection, symptomatic congestive heart failure, uncontrolled hypertension, unstable angina pectoris, cardiac arrhythmia, interstitial lung disease, serious chronic gastrointestinal conditions associated with diarrhea, or psychiatric illness/social situations that would limit compliance with study requirement, substantially increase risk of incurring AEs or compromise the ability of the patient to give written informed consent.<br>- Severe cardiac disorders within 6 months.<br>- Severe liver dysfunction<br>- History of idiopathic pulmonary fibrosis, drug-induced pneumonitis, organizing pneumonia, or evidence of active pneumonitis on screening chest CT-scan.<br>- History of leptomeningeal carcinomatosis. Patients whose brain metastases have been treated may participate provided they show radiographic stability. In addition, any neurologic symptoms that developed either as a result of the brain metastases or their treatment must have resolved or be stable either, without the use of steroids, or are stable on a steroid dose of ≤10mg/day of prednisone or its equivalent for at least 14 days prior to the start of treatment<br>- Positive test for HIV, active hepatitis B or hepatitis C, active tuberculosis.<br>- History of active primary immunodeficiency<br>- Current or prior use of immunosuppressive medication within 14 days before the first dose of study drugs (excepted: intranasal, inhaled, topical steroids or local steroid injection –at physiologic dose does not exceed 10 mg/day of prednisone or its equivalent – steroids as premedication for hypersensitivity reactions).<br>- Receipt of live attenuated vaccine within 30 days prior to the first dose of treatment<br>- Known allergy or hypersensitivity to any of the study drugs or any of the study drug excipients. In order to check all the contraindications of each drugs, please refer to the updated versions of the SmPCs presented in Appendix 9.<br>- Current or prior use of St. John's Wort within 14 days before the first dose of study drugs (St. John's Wort is not allowed during participation in the trial).<br>- Treatment with sorivudine or analogs (brivudine).<br>- Treatment with phenytoin or analogs.<br>- Prior treatment with irinotecan, anti-PD1, anti PD-L1, anti-CLTA4 or other immunotherapy for cancer treatment<br>- Known Uridine Diphosphate Glucuronyltransferase (UGT1A1) or Dihydropyrimidine Dehydrogenase (DPD) enzyme deficiencies.<br>- Active infection requiring intravenous antibiotics at the time of Day 1 of Cycle 1.<br>- Other malignancy within 5 years prior to study enrolment, except for localized cancer *in situ*, basal or squamous cell skin cancer.<br>- Pregnant or breastfeeding female patient.

Study treament	**Safety Run-in phase**<br><br>**1st step: Administered in 5 patients**<br>**FOLFIRI plus durvalumab**<br>- **Durvalumab:** 1500 mg by 1-hour IV infusion.<br>Every 4 weeks until progression<br>**- FOLFIRI (1 course every 2 weeks, until progression):**<br>- Irinotecan: 180 mg/m² by 2-hour IV infusion,<br>- Folinic acid: 400 mg/m² (or 200 mg/m² if Elvorine) by 2-hours IV infusion,<br>- 5-FU bolus: 400 mg/m² by 10-minutes IV bolus,<br>- Continuous 5-FU: 2400 mg/m² by 46-hour IV infusion<br><br>**2nd step: Administered in 6 patients (3 patients per arm)**<br>**FOLFIRI plus durvalumab**<br>- **Durvalumab:** 1500 mg by 1-hour IV infusion.<br>Every 4 weeks until progression<br>**- FOLFIRI (1 course every 2 weeks, until progression):**<br>- Irinotecan: 180 mg/m² by 2-hour IV infusion,<br>- Folinic acid: 400 mg/m² (or 200 mg/m² if Elvorine) by 2-hours IV infusion,<br>- 5-FU bolus: 400 mg/m² by 10-minutes IV bolus,<br>- Continuous 5-FU: 2400 mg/m² by 46-hour IV infusion<br><br>**FOLFIRI plus durvalumab plus tremelimumab**<br>Induction treatment: 4 cycles (*i.e.* 1 course every 4 weeks)<br>- **Durvalumab:** 1500 mg by 1-hour IV infusion - Every 4 weeks.<br>- **Tremelimumab:** 75 mg by 1-hour IV infusion - Every 4 weeks (for only 4 cycles).<br>**- FOLFIRI (1 course every 2 weeks, until progression):**<br>**- Irinotecan: 150 mg/m²** by 2-hour IV infusion<br>- Folinic acid: 400 mg/m² (or 200 mg/m² if Elvorine) by 2-hours IV infusion<br>- 5-FU bolus: 400 mg/m² by 10-minutes IV bolus<br>- Continuous 5-FU: 2400 mg/m² by 46-hour IV infusion<br><br>Tremelimumab is administered for 4 courses (4 months) and then patient will continue to receive FOLFIRI plus durvalumab. In case of progression on FOLFIRI plus durvalumab and disease control, tremelimumab can be re-introduced at investigator discretion.<br>Patient must have 2 weeks of washout period of first-line treatment before receiving the treatment in the trial. Treatment will be repeated every 4 weeks until disease progression, unacceptable toxicity or patient's refusal.<br>All drugs (irinotecan, folinic acid and 5-FU) except durvalumab and tremelimumab will be used in the context of their marketed authorization or recommendations (Thésaurus National de Cancérologie Digestive (www.tncd.org)) in France. Thus, durvalumab and tremelimumab will be provided in this clinical trial. A pharmacovigilance follow-up will be implemented during the study.<br><br>**Phase II:**<br>**Arm A: FOLFIRI plus durvalumab**<br>- **Durvalumab:** 1500 mg by 1-hour IV infusion.<br>Every 4 weeks until progression<br>**- FOLFIRI (1 course every 2 weeks, until progression):**<br>- Irinotecan: 180 mg/m² by 2-hour IV infusion,<br>- Folinic acid: 400 mg/m² (or 200 mg/m² if Elvorine) by 2-hours IV infusion,<br>- 5-FU bolus: 400 mg/m² by 10-minutes IV bolus,<br>- Continuous 5-FU: 2400 mg/m² by 46-hour IV infusion<br><br>**Arm B: FOLFIRI plus durvalumab plus tremelimumab**<br>Induction treatment: 4 cycles (*i.e.* 1 course every 4 weeks)<br>- **Durvalumab:** 1500 mg by 1-hour IV infusion - Every 4 weeks.<br>- **Tremelimumab:** 75 mg by 1-hour IV infusion - Every 4 weeks (for only 4 cycles).<br>**- FOLFIRI (1 course every 2 weeks, until progression):**<br>- Irinotecan: 180 mg/m² by 2-hour IV infusion<br>- Folinic acid: 400 mg/m² (or 200 mg/m² if Elvorine) by 2-hours IV infusion<br>- 5-FU bolus: 400 mg/m² by 10-minutes IV bolus<br>- Continuous 5-FU: 2400 mg/m² by 46-hour IV infusion

	In arm B: tremelimumab is administered for 4 courses (4 months) and then patient will continue to receive FOLFIRI plus durvalumab. In case of progression on FOLFIRI plus durvalumab and disease control, tremelimumab can be re-introduce at investigator discretion.<br><br>Patient must have 2 weeks of washout period of first-line treatment before receiving the treatment in the trial. Treatment will be repeated every 4 weeks until disease progression, unacceptable toxicity or patient's refusal.<br><br>All drugs (irinotecan, folinic acid and 5-FU) except durvalumab and tremelimumab will be used in the context of their marketed authorization or recommendations (Thésaurus National de Cancérologie Digestive (www.tncd.org)) in France. Thus, durvalumab and tremelimumab will be provided in this clinical trial. A pharmacovigilance follow-up will be implemented during the study.
**Safety analysis**	For safety run-in phases, patients will be treated in 5 expert centers with a huge experience in the use of immune checkpoints inhibitors.<br><br>1st step: In order to check the good tolerability of FOLFIRI plus durvalumab combination, 5 patients will be treated by FOLFIRI (irinotecan 180mg/m²) plus durvalumab (1500 mg) in 5 expert centers. The inclusion will be stopped at 5 patients. When the 5th patient will have received 2 cycles of treatment, the safety analysis will be done with all the safety data available at this date. The review will be done by an Independent Data Monitoring Committee (IDMC).<br><br>The decision of IDMC and the data available will be sent to ANSM. We will wait the ANSM approval to re-open the inclusion of patients.<br><br>2nd step: 3 patients per arm will be randomized to receive either FOLFIRI (irinotecan 180 mg/m²) plus durvalumab (1500 mg) or FOLFIRI (**irinotecan 150 mg/m²**) plus durvalumab (1500 mg) plus tremelimumab (75 mg). These 6 patients will be treated in the same 5 expert centers. When the 6th patient will have received 2 cycles of treatment, the safety analysis will be done with all the safety data available at this date (for the 11 patients included in these safety run-in phases). The review will be done by an Independent Data Monitoring Committee (IDMC).<br><br>The decision of IDMC and the data available will be sent to ANSM. We will wait the ANSM approval to open the phase II trial.
**Randomization**	The safety run-in will be done for the first step on an open-labeled part on 5 patients. A simple enrollment process will be performed for the first step of safety run-in.<br><br>For the second step of safety run-in (3 patients per arm) and the phase II, the same randomization process will be performed as described hereinafter.<br><br>The randomization will be done using minimization technique according to the ratio 1:1 and the following factors will be considered for the stratification:<br>- Center<br>- Duration of disease control with previous first-line chemotherapy (no disease control vs < 3 months vs ≥ 3 months)
**Sample size calculation**	No statistical hypotheses for the safety run-in phases. A total of 11 patients will be included in the 2 steps before the randomized phase II will begin.<br><br>The clinical hypotheses for the randomized phase II study are:<br>- H_0: 50% of patients alive and without progression at 4 months is not acceptable.<br>- H_1: 70% of patients alive and without progression at 4 months is expected.<br><br>With a risk α (one-sided) of 5%, a power of 85% and according to the binomial-exact design, 44 evaluable patients are needed by arms (*i.e.* patients randomized and with at least one dose of products taken). Assuming 5% of non-evaluable patients or lost to follow-up, 47 patients will be included by arms (**94 patients in total**).<br><br>Taking into account the 11 patients included in the safety run-in phases, 105 patients will be included in the trial.

**Statistical analysis (generality)**	**Safety run-in:**<br>Listing of baseline patient characteristics, treatments and safety data will be provided to IDMC (as well to ANSM) at the time of the analyses including dose of treatments, toxicities and serious adverse events.<br>**Phase II study:**<br>Primary endpoint will be analysed on the modified intent-to-treat population (patients with at least one dose of treatment). All the baseline characteristics will be described on the overall population and by treatment arm. Description of toxicities and other baseline variables will be done using usual statistics: for continuous variables: mean, standard deviation, median, inter-quartile interval and range, and for categorical variables frequencies and percentages.<br><br>Survival analyses will be estimated using Kaplan-Meier method. A detailed Statistical Analysis Plan will be written before the database lock.
**Biological study**	**Only for randomized phase II:**<br>- Blood (plasma) and tumor samples will be collected in all patients in order to allow translational research projects (Centre de Ressource Biologique EPIGENETEC, UMR-S 1147, Paris, France, Headed by Prof. Pierre Laurent-Puig) in order to identify predictive biomarkers of treatment efficacy including at least (for more details see “ancillary studies”): microsatellite instability (tumor DNA and immunohistochemistry), immune response (including PD-L1 and PD-L2) and immune score (immunohistochemistry), circulating tumor DNA (baseline and kinetic), tumor mutation load and gastric molecular sub-groups.<br><br>- Stool samples will be collected prospectively in all patients in order to allow analysis of microbiota (16S rRNA to identification of bacteria composing the intestinal microbiota of patients).
**Number of patients**	Safety Run-in phases : 11 patients ;<br>Randomized phase II study : 94 patients<br>Overall study (safety run-in and phase II) : 105 patients
**Duration of inclusion and length of participation for each patient**	Theoretical rate of inclusion: 4 per month<br>Safety run-in: 5 expert centers selected<br>Safety analysis of the first 11 patients included: – Q1 2019 to Q1 2020<br><u>Phase II study:</u><br>Number of centers: 50 (around 30 active centers)<br>Theoretical start of inclusion: Q1 2020<br>Theoretical end of inclusion: Q1 2022<br>End of the trial (primary and secondary endpoint analysis): Q2 2023

EXAMINATION AND FOLLOW-UP SCHEDULE

	BEFORE TREATMENT	DURING TREATMENT and in case of treatment stop without radiological progression (e.g. toxicity or patient refusal)		AFTER TREATMENT END (for radiological progression)
	During the 14 days preceding the start of treatment	Before each course of treatment	Every 8 weeks (at each evaluation)	Every 2-3 months up to death
**Clinical and biological informed consent**	X			
**Biopsies or tumor block, fixed in paraffin**	**X****			
**CLINICAL EXAMINATION**				
Weight, height, body surface area, BP, pulse, temperature	X	X	X	
ECOG Performance statut	X	X	X	
Evaluation of toxicities NCI-CTC Version 4.0 (Appendix 8)		X	X (and 30 days after end of treatment)	X (until 12 months after the end of treatment)
QLQ-C30 and STO-22 questionnaires (Appendix 3,4) [p]	X		X	
**BIOLOGICAL ASSESSMENT**				
Laboratory assessment	X***	X*****	X***	
Hepatitis B,C and HIV test	X			
Pregnancy test	X	X******		
CAE and CA 19.9 markers	X		X	
DPD status*	X****			
**PARACLINICAL REVIEWS**				
Thoraco-abdominal-pelvic CT scan or MRI	X****		X*******	X
ECG	X****			
Determination of MSI/MSS status or tumoral block available	X			
**BIOLOGICAL STUDY**				
Blood samples (2 tubes/sample) [p]	X		X********	
Stools [p]	X (5 days before first course)		X (only at W8 – 5 days before the evaluation)	
**FUTURE LINES**				
Start and end dates of treatment and the type of treatment of the subsequent lines will be completed in the CRF				X

[p]: phase II only items are indicated and highlighted in red

*: DPD status must be determined only for patients with unknown DPD status and who have not received 5FU previously, and also for patients who have previously received 5FU with serious adverse event (not necessary for patients who have previously received 5FU with no significant adverse event)

** : The investigator need to ensure that tumor tissues are available and sent after the patient randomization

***: CBC, platelets, liver panel (bilirubin (total and conjugated), ALT, AST, ALP, GGT, LDH), serum creatinine, creatinine clearance (MDRD - Appendix 5), TSH, blood protein, albumin, prealbumin, CRP, coagulation (PT, PTT), serum electrolytes (sodium, potassium, calcium, magnesium), lipase, glucose, urea, urinalysis (urine strip – check of the protein level, if more than 2 crosses then check the proteinurea on 24h).

****: Within 3 weeks prior to randomization

*****: CBC, platelets, urea, liver panel (bilirubin (total and conjugated), ALT, AST, ALP, GGT), bilirubin (total and conjugated), serum electrolytes (sodium, potassium, calcium, magnesium), serum creatinine, creatinine clearance (MDRD - Appendix 5).

******: For women with childbearing potential a pregnancy test will be performed each month during the treatment duration

*******: Use same technique in each imaging examination as that of the initial evaluation. **Send an anonymized copy of images in CD-ROM format to FFCD, 7 bd Jeanne d'Arc, BP 87900, 21079 DIJON cedex (centralized review for the secondary end point and the ancillary study)**

********: Blood sample at 4 weeks **for ancillary studies**

1. STUDY OBJECTIVE

1.1. **Safety run-in phases** objectives

Validate the good tolerability of treatments combination in 2 steps:

- 1st step: treated 5 patients with FOLFIRI (irinotecan at 180 mg/m²) + durvalumab
- 2nd step: randomized 6 patients between FOLFIRI (irinotecan at 180 mg/m²) + durvalumab versus FOLFIRI (**irinotecan at 150 mg/m²**) + durvalumab + tremelimumab (3 patients per arm)

Patients will be treated in 5 expert centers with a huge experience in the use of immune checkpoints inhibitors. Inclusions will be stopped at each step for safety analyses.

1.2. Primary objective of phase II only Percentage of patients alive and without progression at 4 months of FOLFIRI plus durvalumab versus FOLFIRI plus durvalumab plus tremelimumab in patients with advanced-stage gastric or gastro-oesophageal junction adenocarcinoma and who progressed after a first line chemotherapy (based on RECIST 1.1 rating scale evaluated by the investigator).

1.3. Secondary objectives for phase II study

- Percentage of patients alive and without progression at 4 months according to centralized review
- Overall survival (OS)
- Time to strategy failure
- Safety profile
- Quality of life (QoL)
- Time to progression (TTP), the progression-free survival (median PFS), the best objective response rate (BRR) and disease control rate (DCR) according to the investigator and centralized review (according RECIST V1.1 and iRECIST criteria)
- Efficacy endpoints (OS, PFS, TTP, BRR and DCR) according to the expression PD-L1 and others biomarkers (see biological study)

Phase II only: Ancillary biological studies (optional)
Blood and tumor samples will be collected in all patients in order to allow translational research projects (Centre de Ressource Biologique EPIGENETEC, UMR-S 1147, Paris, France, Headed by Prof. Pierre Laurent-Puig):
- Biomarkers analysis (for more details see "ancillary studies" – Appendix 2): microsatellite instability, immune response/immune score, circulating tumor DNA, tumor mutation load, gastric molecular sub-groups, expression and/or amplification of PD-L1 and PD-L2).
- Stool samples will be collected prospectively in all patients in order to allow analysis of microbiota (16S rRNA to identification of bacteria composing the intestinal microbiota of patients).

2. PATIENT SELECTION ON REGISTRATION/RANDOMIZATION

Inclusion and non-inclusion criteria are the same for the safety run-in phases and the randomized phase II trial.

2.1. Inclusion criteria

- Age ≥ 18 years.
- Body weight > 30kg.
- Histologically proven advanced-stage unresectable adenocarcinoma of the stomach or the GEJ (Siewert II or III).
- Known MSS/MSI status or tumor tissue available (frozen or paraffin-embedded, primary tumors or metastases) in order to allow determination of MSS/MSI status. The investigator needs to ensure that tumor tissues will be sent after patient randomization.

- Failure to platinium-based 1st line therapy with or without trastuzumab or early recurrent disease after surgery with neo-adjuvant and/or adjuvant platinium-based chemotherapy (within 6 months of the end of chemotherapy) or progression during neo-adjuvant and/or adjuvant platinium-based chemotherapy.
- Eligible for a second-line treatment with irinotecan and 5-FU.
- Measurable or non-measurable lesion according to the Response Evaluation Criteria in Solid Tumors (RECIST 1.1).
- Eastern Cooperative Oncology Group (ECOG) performance status 0-1.
- Adequate organ function: ANC ≥ 1.5 x 10^9/L, haemoglobin ≥ 9 g/dL, platelets ≥ 100 x 10^9/L, AST/ALT ≤ 3 x ULN (≤ 5 x ULN in case of liver metastase(s)), GGT ≤ 3 x ULN (≤ 5 x ULN in case of liver metastase(s)), bilirubin ≤ 1.5 x ULN, creatinin clearance > 40 mL/min (MDRD).
  Evidence of post-menopausal status or negative urinary or serum pregnancy test for female pre-menopausal patients.
- Man and woman who childbearing potential agrees to use two methods (one for the patient and one for the partner) of medically acceptable forms of contraception during the study and for 6 months after the last treatment intake.
- Patient is able to understand, sign, and date the written informed consent form at the screening visit prior to any protocol-specific procedures performed.

2.2. Non-inclusion criteria

- Concurrent enrolment in another clinical study – unless it is an observational study or during the follow-up period of an interventional study.
- Receipt of the last dose of anticancer therapy ≤ 2 weeks prior to the first dose of study drug.
- Radiotherapy within 4 weeks prior to the first dose of treatment.
- History of chronic inflammatory bowel disease (IBD).
- Current or prior bowel obstruction within 28 days before the first dose of study drugs.
- Any unresolved significant toxicity NCI CTCAE v4.0 ≥ grade 2 from previous anticancer therapy.
- Concurrent use of hormonal therapy for non–cancer-related conditions is acceptable
- Major surgical procedure (e.g. exploratory laparoscopy is not considered as a major surgical procedure) within 28 days prior to the first dose of treatment.
- Prior allogeneic bone marrow transplantation or prior solid organ transplantation.
- Active or prior documented autoimmune or inflammatory disorders (patients with alopecia, vitiligo, controlled hypo or hyperthyroidism, any chronic skin condition not requiring immunosuppressant therapy are eligible). Patients without active disease in the last 5 years may be included.
- Uncontrolled intercurrent illness, including but not limited to, ongoing or active infection, symptomatic congestive heart failure, uncontrolled hypertension, unstable angina pectoris, cardiac arrhythmia, interstitial lung disease, serious chronic gastrointestinal conditions associated with diarrhea, or psychiatric illness/social situations that would limit compliance with study requirement, substantially increase risk of incurring AEs or compromise the ability of the patient to give written informed consent.
- Severe cardiac disorders within 6 months.
- Severe liver dysfunction
- History of idiopathic pulmonary fibrosis, drug-induced pneumonitis, organizing pneumonia, or evidence of active pneumonitis on screening chest CT-scan.
- History of leptomeningeal carcinomatosis.
- Positive test for HIV, active hepatitis B or hepatitis C, active tuberculosis.
- History of active primary immunodeficiency
- Current or prior use of immunosuppressive medication within 14 days before the first dose of study drugs (excepted: intranasal, inhaled, topical steroids or local steroid injection –at physiologic dose does not exceed 10 mg/day of prednisone or its equivalent – steroids as premedication for hypersensitivity reactions).
- Receipt of live attenuated vaccine within 30 days prior to the first dose of treatment
- Known allergy or hypersensitivity to any of the study drugs or any of the study drug excipients. In order to check all the contraindications of each drugs, please refer to the updated versions of the SmPCs presented in Appendix 9.
- Current or prior use of St. John's Wort within 14 days before the first dose of study drugs (St. John's Wort is not allowed during participation in the trial).
- Treatment with sorivudine or analogs (brivudine).

- Treatment with phenytoin or analogs.
- Prior treatment with irinotecan, anti-PD1, anti PD-L1, anti-CLTA4 or other immunotherapy for cancer treatment regardless of treatment arm assignment.
- Known Uridine Diphosphate Glucuronyltransferase (UGT1A1) or Dihydropyrimidine Dehydrogenase (DPD) enzyme deficiencies.
- Active infection requiring intravenous antibiotics at the time of Day 1 of Cycle 1.
- Other malignancy within 5 years prior to study enrolment, except for localized cancer *in situ*, basal or squamous cell skin cancer.
- Pregnant or breastfeeding female patient.

3. INCLUSION ASSESSMENT

The inclusion assessment must be conducted during the 14 days before registration/randomization. This does not apply to morphological examinations and ECG, which may be conducted during the 3 weeks before registration/randomization.

Quality-of-life questionnaires – **PHASE II ONLY**
- QLQ-C30 version 3.0 – QLQ-STO22 questionnaires: to be completed by the patient before randomization (same day or within 14 days before randomization but in any case at least before the first course of treatment)

Clinical examination:
- Measurement of weight, height and body surface area
- ECOG performance status (Appendix 5)
- Vital signs: BP, pulse, temperature

Laboratory assessment at least 14 days before registration/randomization, comprising:
- CBC, platelets, TP
- Liver panel comprising GGT, ALP, AST, ALT, total and conjugated bilirubin, and LDH
- Creatinine and creatinine clearance (MDRD) - (Appendix 5)
- TSH (T3,T4), blood protein, albumin, prealbumin and CRP
- Coagulation (PT, PTT)
- Lipase, glucose
- Serum electrolytes (sodium, potassium, calcium, magnesium), urea
- Hepatitis B, C and HIV test
- CEA, CA19-9 markers
- Pregnancy test if women of childbearing age
- Urine Strip (proteinurea)
- DPD status must be determined according to the center practice **only for patients with unknown DPD status and who have not received 5FU previously, and also for patients who have previously received 5FU with serious adverse event and suspected DPD deficit (not necessary for patients who have previously received 5FU with no significant adverse event)**

Morphological examinations and ECG **within 3 weeks prior to registration/randomization**:
- Thoracic-abdominal-pelvic CT-scan (TAP CT-scan or abdominal MRI + thoracic CT-scan without injection if injected CT-scan contraindicated)
- ECG

For Phase II only:

Send an anonymous copy of CD ROM to the FFCD, 7 bd Jeanne d’Arc, BP 87900, 21079 DIJON CEDEX (centralized review for secondary criteria)

The investigator needs to ensure that tumor blocks (or tissues) are available and sent after the patient randomization (see Chapter 9 for logistics).
Sending the pre-filled letter to your anatomopathologist and the anatomopatologist fax to the FFCD the sample sheet at the +33 (0)3 80 38 18 41.
FFCD sends a max letter for sending tumor blocks to Centre de Ressources Biologiques EPIGENETEC (Unité UMR-S 1147, 45, rue des Saints-Pères - 75006 PARIS, France)

<u>**For the biological ancillary study**</u>

- Cell- free DNA tubes of blood are taken:
  - before the first treatment course
  - at 4 weeks (D28)
  - at progression
- Stool sample
  - 1 sample – in the 5 days prior the 1st course of treatment (W0)
  - 1 sample – in the 5 days prior the 3rd course of treatment (W8)

The rational and logistics of this study are described in Appendix 2 and Chapter 9.

4. REGISTRATION/RANDOMIZATION

For the first step of the safety run-in a registration of patient will be performed. The randomization procedure will be performed only for the 2nd step of the safety run-in phase and for the Phase II study.

For both registration and randomization, the process remains the same and is described hereinafter.

After the consent form signature and the validation of the initial baseline assessments results, eligible patients will be registered/randomized at the FFCD data center, **CRGA (Centre de Randomisation – Gestion – Analyse)**.

The investigator will fax the completed and signed registration/randomization form to the FFCD data center:

Monday to Friday from 9 am to 6 pm
Fax: + 33 (0)3 80 38 18 41/Tel: + 33 (0)3 80 66 80 13

A registration/randomization confirmation will be sent back to the investigator and to the pharmacist with the patient registration number and the arm allocated by the randomization.
After registration/randomization, treatment should begin as soon as possible and within a maximum period of 10 days. A case report form will be sent automatically after each registration/randomization and at the center's opening.

Stratification
The randomization will be done using minimization technique according to the ratio 1:1 and the following factors will be considered for the stratification:
- Center
- Duration of disease control to previous first-line chemotherapy (no disease control vs < 3 months vs ≥ 3 months)

Immunotherapy patient card
The investigator can give an "immunotherapy patient card" once the patient registration/randomization performed. This card will inform that the patient is being treated by immunotherapy and will follow the patient throughout the clinical study. This is useful especially if the patient must be treated urgently in another center.

5. STUDY DESIGN

5.1. Safety run-in

1st step:

5 patients will be included first according to the following treatment regimen:

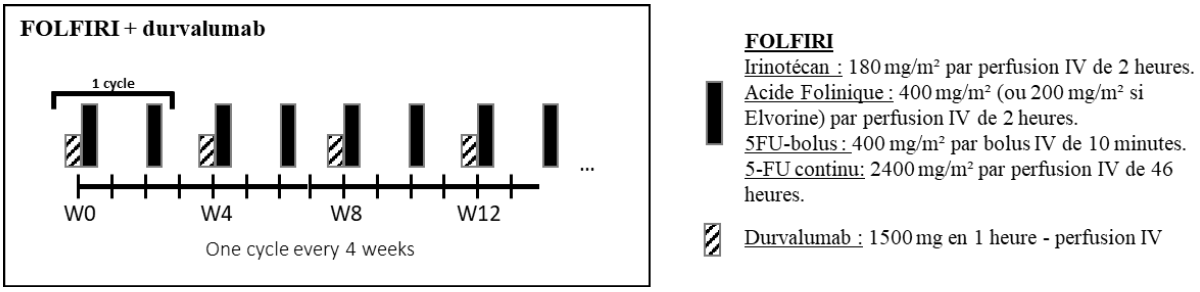

2nd step:

6 patients (3 patients per arm) will be randomized according to the following treatment regimen:

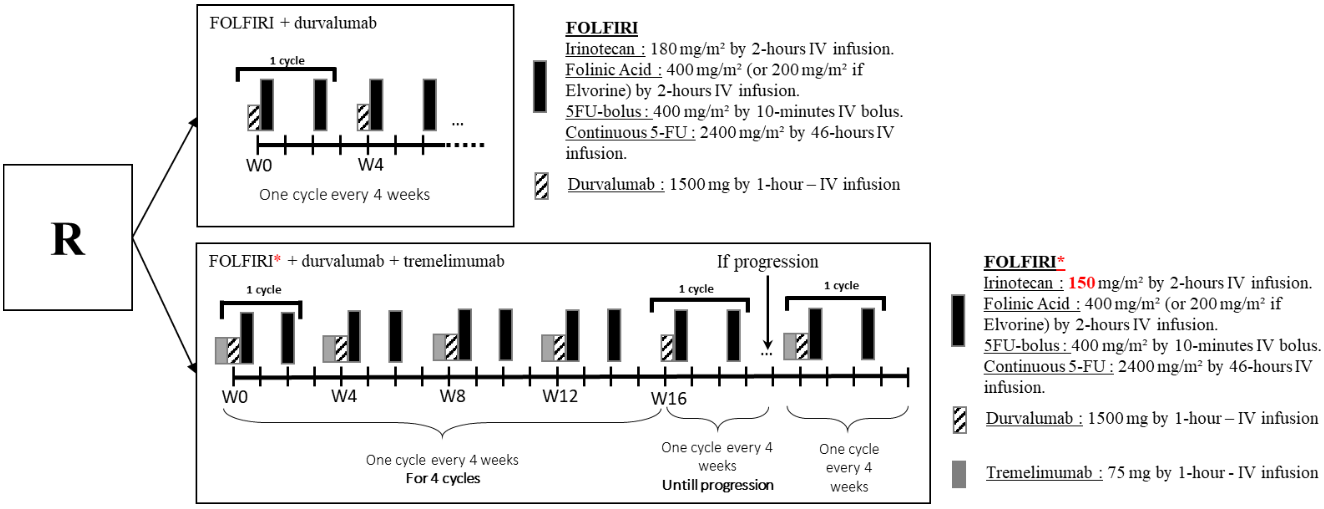

Tremelimumab is administered for 4 cycles (4 months) and then patient will continue to receive FOLFIRI plus durvalumab. In case of progression on FOLFIRI plus durvalumab and disease control, tremelimumab can be re-introduced at investigator discretion for 4 courses. Only one re-introduction is authorized.

Patient must have 2 weeks of washout period of first-line treatment before treatment in the trial (4 weeks if investigational product in first-line setting, previous immunotherapy is not allowed). Treatment will be repeated every 4 weeks until disease progression, unacceptable toxicity or patient's refusal.

5.2. Phase II study

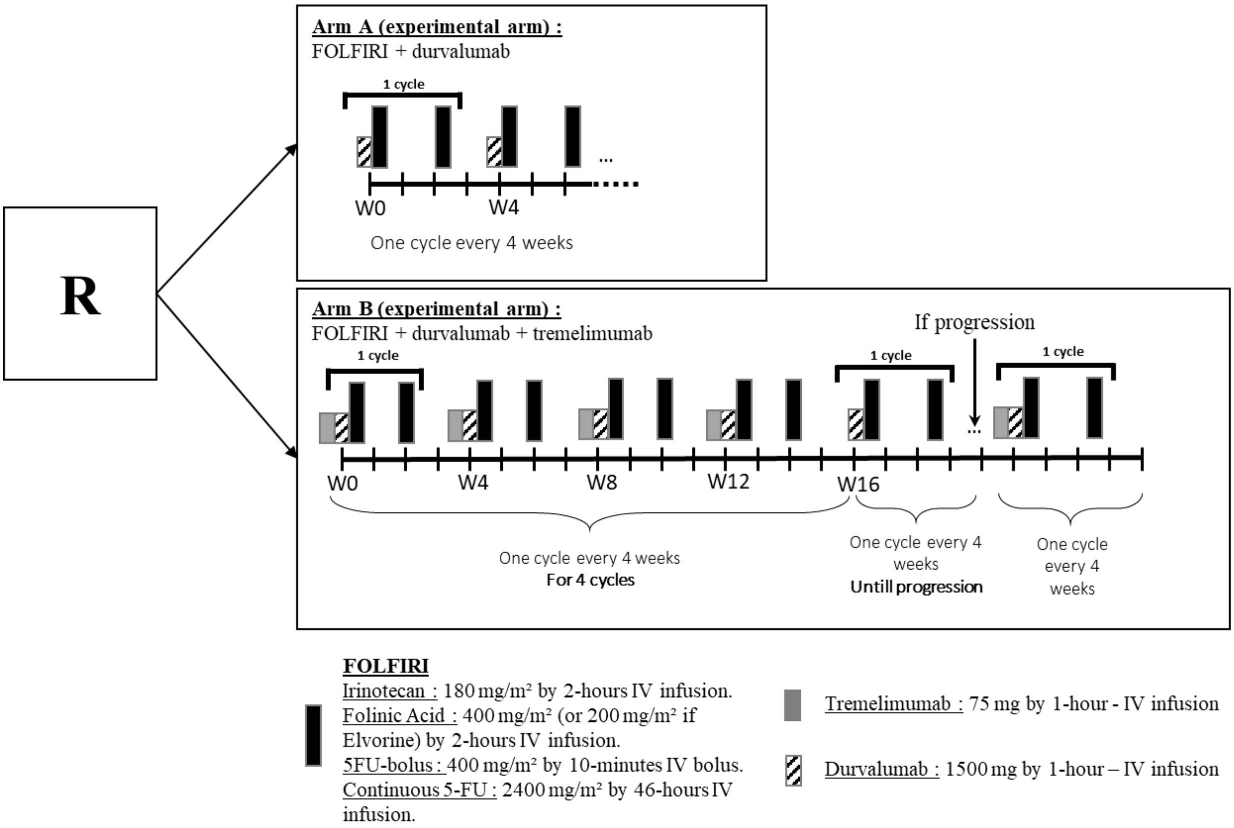

In arm B: Tremelimumab is administered for 4 cycles (4 months) and then patient will continue to receive FOLFIRI plus durvalumab. In case of progression on FOLFIRI plus durvalumab and disease control, tremelimumab can be re-introduced at investigator discretion for 4 courses. Only one re-introduction is authorized.

Patient must have 2 weeks of washout period of first-line treatment before treatment in the trial (4 weeks if investigational product in first-line setting, previous immunotherapy is not allowed). Treatment will be repeated every 4 weeks until disease progression, unacceptable toxicity or patient's refusal.

6. TREATMENTS

Both durvalumab and tremelimumab will be provided by FFCD whereas 5-FU, folinic acid and irinotecan will be sampled on commercial stock.

6.1. Description, packaging and labeling of durvalumab

Durvalumab will be supplied by FFCD as a 500-mg vial solution for infusion after dilution.
The solution contains 50 mg/mL durvalumab, 26 mM histidine/histidine hydrochloride, 275 mM trehalose dihydrate, and 0.02% weight/volume (w/v) polysorbate 80; it has a pH of 6.0. The nominal fill volume is 10.0 mL.
Investigational product vials are stored at 2°C to 8°C and must not be frozen. Drug product should be kept in secondary packaging until use to prevent excessive light exposure.

The dose of durvalumab for administration must be prepared by the Investigator's or site's designated investigational product manager using aseptic technique. Total time from needle puncture of the durvalumab vial to the start of administration should not exceed:

- 24 hours at 2°C to 8°C
- 4 hours at room temperature

Infusion solution must be allowed to equilibrate to room temperature prior to commencement of administration.
A dose of 1500 mg (for patients >30kg in weight) will be administered using an IV bag containing 0.9% (w/v)

saline or 5% (w/v) dextrose, with a final durvalumab concentration ranging from 1 to 15 mg/mL, and delivered through an IV administration set with a 0.2- or 0.22-µm in-line filter. Add 30.0 mL of durvalumab (*i.e.* 1500 mg of durvalumab) to the IV bag. The IV bag size should be selected such that the final concentration is within 1 to 15 mg/mL. Mix the bag by gently inverting to ensure homogeneity of the dose in the bag.

If weight falls to ≤ 30 kg weight-based dosing at 20 mg/kg will be administered using an IV bag containing 0.9% (w/v) saline or 5% (w/v) dextrose, with a final durvalumab concentration ranging from 1 to 15 mg/mL, and delivered through an IV administration set with a 0.2- or 0.22-µm in-line filter.

Standard infusion time is 1 hour. In the event that there are interruptions during infusion, the total allowed infusion time should not exceed 8 hours at room temperature.
Do not co-administer other drugs through the same infusion line.

The IV line will be flushed with a volume of IV diluent equal to the priming volume of the infusion set used after the contents of the IV bag are fully administered, or complete the infusion according to institutional policy to ensure the full dose is administered and document if the line was not flushed.
If either preparation time or infusion time exceeds the time limits a new dose must be prepared from new vials. Durvalumab does not contain preservatives, and any unused portion must be discarded.

6.2. Description, packaging and labeling of tremelimumab

Tremelimumab will be supplied by FFCD as a 400-mg vial solution for infusion after dilution.
The solution contains 20 mg/mL tremelimumab, 20 mM histidine/histidine hydrochloride, 222 mM trehalose dihydrate, 0.27 mM disodium edetate dihydrate, and 0.02% weight/volume (w/v) polysorbate 80; it has a pH of 5.5.
The nominal fill volume is 20.0 mL. Investigational product vials are stored at 2°C to 8°C and must not be frozen. Drug product should be kept in secondary container until use to prevent excessive light exposure.

The dose of tremelimumab for administration must be prepared by the Investigator's or site's designated investigational product manager using aseptic technique. Total time from needle puncture of the tremelimumab vial to the start of administration should not exceed:

- 24 hours at 2°C to 8°C
- 4 hours at room temperature

Infusion solution must be allowed to equilibrate to room temperature prior to commencement of administration.

A dose of 75 mg (for patients >30kg in weight) will be administered using an IV bag containing 0.9% (w/v) saline or 5% (w/v) dextrose, with a final tremelimumab concentration ranging from 0.10 to 10 mg/mL, and delivered through an IV administration set with a 0.2- or 0.22-µm in-line filter. Add 3.8 mL (ie,75 mg of tremelimumab, with the dose volume rounded to the nearest tenth mL) to the IV bag. The IV bag size should be selected such that the final concentration is within 0.10 to 10 mg/mL. Mix the bag by gently inverting to ensure homogeneity of the dose in the bag.
If weight falls to ≤ 30 kg, weight-based dosing at 1 mg/kg will be administered using an IV bag containing 0.9% (w/v) saline or 5% (w/v) dextrose, with a final tremelimumab concentration ranging from 0.10 to 10 mg/mL, and delivered through an IV administration set with a 0.2- or 0.22-µm in-line filter.

Standard infusion time is 1 hour. In the event that there are interruptions during infusion, the total allowed infusion time should not exceed 8 hours at room temperature.
Do not co-administer other drugs through the same infusion line.
The IV line will be flushed with a volume of IV diluent equal to the priming volume of the infusion set used after the contents of the IV bag are fully administered, or complete the infusion according to institutional policy to ensure the full dose is administered and document if the line was not flushed.
If either preparation time or infusion time exceeds the time limits a new dose must be prepared from new vials. Tremelimumab does not contain preservatives, and any unused portion must be discarded.

6.3. FOLFIRI + durvalumab (1st and 2nd steps of the safety run-in phase and Arm A)

No prophylactic treatment is necessary for durvalumab.

Prophylactic treatment for FOLFIRI is administered according the standard centre clinical practice.

Primary prophylaxis with G-CSF is not necessary but is allowed, notably if febrile neutropenia occurred during first-line therapy. Secondary prophylaxis with G-CSF is left up to the investigator's judgment and according to hematological toxicity of chemotherapy and the patient's clinical characteristics.

Recommendation on dose capping: Centers will perform dose capping according to their habits. It might be recommended to do not cap the dose at 2m² if the patient presents an important muscle mass. However, if the patient presents an important fat mass, a 2m² capping might be considered.

- **Durvalumab**: 1500 mg by 1-hour IV infusion – 1 course every 4 weeks (= 1 cycle)
- **FOLFIRI** – one course every 2 weeks:
    - Irinotecan: 180 mg/m² by 2-hours IV infusion
    - Folinic acid: 400 mg/m² (or 200 mg/m² if Elvorine) by 2-hours IV infusion
    - 5-FU bolus: 400 mg/m² by 10-minutes IV bolus
    - Continuous 5-FU: 2400 mg/m² by 46-hours IV infusion

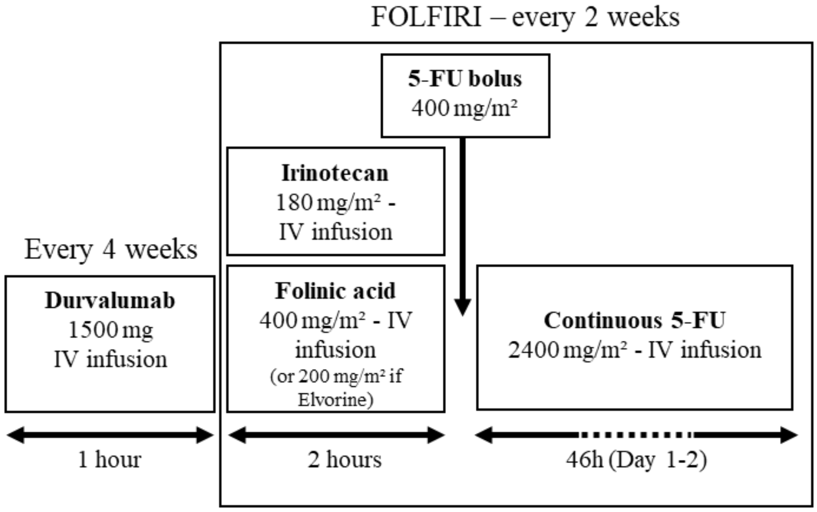

A minimum of 30 minutes should be observed between durvalumab and FOLFIRI administration.

6.4. FOLFIRI + durvalumab + tremelimumab (2nd step of the safety run-in phase and Arm B)

No prophylactic treatment is necessary for durvalumab and tremelimumab.
Prophylactic treatment for FOLFIRI is administered according the standard center clinical practice.
Primary prophylaxis with G-CSF is not necessary but is allowed, notably if febrile neutropenia occurred during first-line therapy. Secondary prophylaxis with G-CSF is left up to the investigator's judgement and according to haematological toxicity of chemotherapy and the patient's clinical characteristics.

Recommendation on irinotecan, folinic acid, 5-FU bolus and continuous 5-FU dose capping. Centers will perform dose capping according to their habits. It might be recommended to do not cap the dose at a body surface area of 2 m² if the patient presents an important muscle mass. However, if the patient presents an important fat mass, a 2 m² capping might be considered.

6.4.1. For the 2nd step of the safety run-in phase

- **Tremelimumab**: 75 mg by 1-hour IV infusion – 1 cycle (*i.e.* every 4 weeks) for only 4 cycles

- **Durvalumab**: 1500 mg by 1-hour IV infusion – 1 cycle (*i.e.* every 4 weeks)
- **FOLFIRI** – one course every 2 weeks:
  - Irinotecan: **150 mg/m²** by 2-hours IV infusion
  - Folinic acid: 400 mg/m² (or 200 mg/m² if Elvorine) by 2-hours IV infusion
  - 5-FU bolus: 400 mg/m² by 10-minutes IV bolus
  - Continuous 5-FU: 2400 mg/m² by 46-hours IV infusion

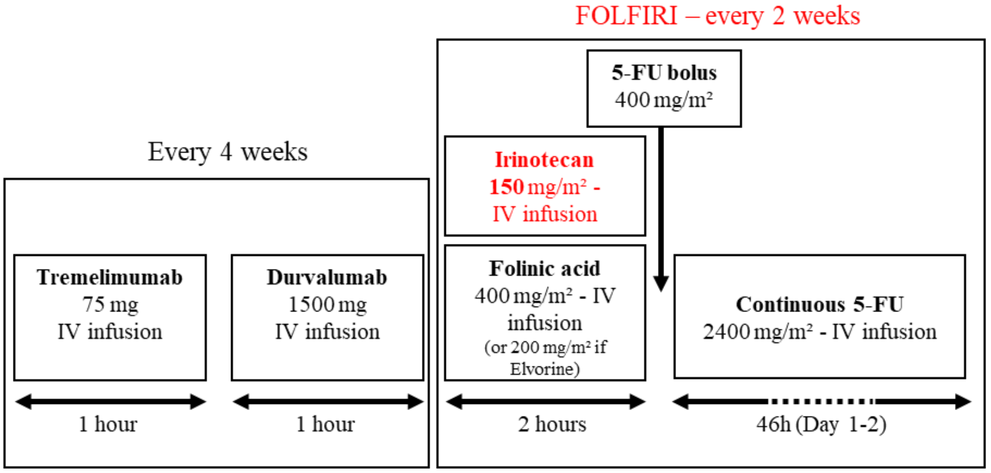

A minimum of 30 minutes should be observed between durvalumab administration and FOLFIRI.

Tremelimumab will be administered first; the durvalumab infusion will start approximately 1 hour (maximum 2 hours) after the end of the tremelimumab infusion. Standard infusion time for each is 1 hour. In the event that there are interruptions during infusion, the total allowed time should not exceed 8 hours at room temperature per infusion. If there are no clinically significant concerns after the first cycle, then, at the discretion of the investigator, all other cycles of durvalumab can be given immediately after the tremelimumab infusion has finished.

6.4.2. For the phase II study

- **Tremelimumab**: 75 mg by 1-hour IV infusion – 1 cycle (*i.e.* every 4 weeks) for only 4 cycles
- **Durvalumab**: 1500 mg by 1-hour IV infusion – 1 cycle (*i.e.* every 4 weeks)
- **FOLFIRI** – one course every 2 weeks:
  - Irinotecan: 180 mg/m² by 2-hour IV infusion
  - Folinic acid: 400 mg/m² (or 200 mg/m² if Elvorine) by 2-hours IV infusion
  - 5-FU bolus: 400 mg/m² by 10-minutes IV bolus
  - Continuous 5-FU: 2400 mg/m² by 46-hours IV infusion

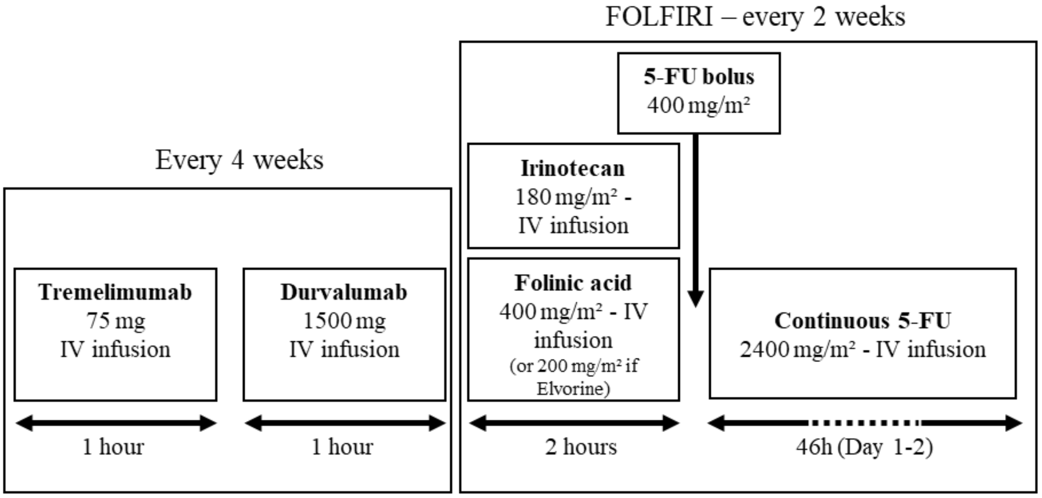

A minimum of 30 minutes should be observed between durvalumab administration and FOLFIRI.

Tremelimumab will be administered first; the durvalumab infusion will start approximately 1 hour (maximum 2 hours) after the end of the tremelimumab infusion. Standard infusion time for each is 1 hour. In the event that there are interruptions during infusion, the total allowed time should not exceed 8 hours at room temperature per infusion. If there are no clinically significant concerns after the first cycle, then, at the discretion of the investigator, all other cycles of durvalumab can be given immediately after the tremelimumab infusion has finished.

7. DOSE ADJUSTMENT BASED ON TOXICITY

The toxicities requiring dose adjustments will all be evaluated according to the scale NCI-CTCAE v4.0 (Appendix 8). Dose adjustments based on toxicity are the same for the 1st and 2nd step of the safety run-in phases and randomized phase II study.

7.1. Criteria that must be met before each subsequent cycle (D1 of each cycle)

G-CSF primary prophylaxis in both arms will be at the investigator discretion according to the previous hematological toxicities and the clinical characteristics of the patient.

Definition of febrile neutropenia: fever > 38.5 °C in medullary hypoplasia period (ANC < 500/mm^3)

Criteria that must to be met before each subsequent course of treatment for chemotherapy:

- ANC ≥ 1500 /mm^3
- Platelet count ≥ 100 000/mm^3
- Digestive toxicities ≤ grade 1

As long as these criteria have not been met, symptomatic treatment should be optimised and a CBC will be conducted every 7 days until obtaining the required levels. The treatment can then be carried out.

Concerning administration of durvalumab ± tremelimumab, patients should not experienced severe immune-related adverse event (see paragraph 7.4).

Criteria for stopping treatment:
Treatment may be discontinued if the investigator considers that it is necessary, in case of major toxicity which no longer makes it possible to continue treatment, in case of a serious or unexpected event requiring discontinuation of treatment, disease progression, withdrawal of consent, refusal of patient or in case of pregnancy. If treatment is delayed more than 4 courses (56 days), the study treatment will be discontinued, but patient will continue to be followed in the setting of the protocol.

7.2. Dose adjustment based on toxicities observed during the rest period

Dosage adjustments are needed depending on the maximum grade of toxicity observed between courses of treatment. The treatments will only begin when the criteria required before implementation of any new treatment is obtained (see paragraph 7.1).

The occurrence of grade 4 toxicity (excluding hematologic toxicities or other manageable toxicity) shall require the permanent discontinuation of the study treatments unless the investigator considers that there is an interest for the patient to continue with the rest of the treatment when the alleged responsibility of the toxicity observed is not deducted. The recourse treatments will be at the discretion of the investigator. In all cases, the patient will continue to be monitored as part of the protocol according to the protocol schedule.

If one drug is stopped due to toxicity, other drugs will continue to be administered.

Regimen allowed			
5-FU/LV	Irinotecan	Durvalumab	Tremelimumab
**X**			
**X**		**X**	
**X**		**X**	**X**
**X**	**X**		
**X**	**X**	**X**	
		**X**	
**Regimen not allowed**			
5-FU/LV	Irinotecan	Durvalumab	Tremelimumab
	**X**		
	**X**	**X**	**X**
	**X**	**X**	
	**X**		**X**
		**X**	**X**
**X**			**X**
		**X**	**X**
**X**	**X**		**X**
			**X**

7.3. Dose adjustment based on infusion-related reaction

In the event of a ≤ grade 2 infusion-related reaction, the infusion rate of study drug may be decreased by 50% or interrupted until resolution of the event and re-initiated at 50% of the initial rate until completion of the infusion. For patients with a ≤ grade 2 infusion-related reaction, subsequent infusions may be administered at 50% of the initial rate. Acetaminophen and/or an antihistamine (e.g., diphenhydramine) or equivalent medications per institutional standard may be administered at the discretion of the investigator. If the infusion-related reaction is ≥ grade 3 or higher in severity, study drug will be discontinued. For management of patients who experience an infusion reaction, please refer to the toxicity and management guidelines in Appendix 8.

As with any antibody, allergic reactions to dose administration are possible. Appropriate drugs and medical equipment to treat acute anaphylactic reactions must be immediately available, and study personnel must be trained to recognize and treat anaphylaxis. The study site must have immediate access to emergency resuscitation teams and equipment in addition to the ability to admit patients to an intensive care unit if necessary.

7.4. Dose adjustment of FOLFIRI

Please refer to the references to the updated SmPCs for the products used for issues of patient management, particularly with respect to contraindications, warnings and precautions for use, dose adjustment in the event of toxicity, monitoring of patients, duration of contraception and medicinal products that are forbidden or to be used with precautions. The links to updated versions of the SmPCs are provided in Appendix 9 of this protocol.

Dose adjustment of FOLFIRI according to the higher hematological toxicity occurring the day of the course. In case of grade 4 or febrile neutropenia, the course must be reported until grade ≤1.

Grade toxicity<br>**(NCI-CTCAE v4)**	5-FU	Irinotecan
Neutropenia,		

thrombocytopenia<br>Grade 2[a]	- Bolus reduction of 50%	- No modification
Grade 3[a]	- Bolus suppression, reduction of 25% of continuous 5-FU	- No modification
Grade 4[b]	- Bolus suppression, reduction of 25% of continuous 5-FU	- Reduction of 25%
Febrile neutropenia[b]	- Bolus suppression, reduction of 25% of continuous 5-FU	- Reduction of 25%

[a] Discuss about G-CSF prescription if persistence of neutropenia < 1500/mm^3 after 1-week postponement
[b] Discuss about G-CSF prescription if Grade 4 neutropenia or febrile neutropenia

Dose adjustment of FOLFIRI according to maximal toxicity in the intercourse

Grade toxicity (NCI-CTCAE v4)	5-FU	Irinotecan
Neutropenia, thrombocytopenia<br>Grade 2	- No modification	- No modification
Grade 3	- No modification	- No modification
Grade 4[b]	- Bolus suppression	- No modification
Febrile neutropenia[b]	- Bolus suppression, reduction of 25% of continuous 5-FU	- Reduction of 25%

[a] Discuss about G-CSF prescription if Grade 4 neutropenia or febrile neutropenia

Other toxicities according to maximal toxicity in the intercourse

Grade toxicity (NCI-CTCAE v4)	5-FU	Irinotecan
Diarrhea despite maximum symptomatic treatment<br>Grade 2	- Bolus reduction of 50%	- Reduction of 25%
Grade 3	- Bolus suppression, reduction of 50% of continuous 5-FU	- Reduction of 25%
Grade 4	- Discuss discontinuation of chemotherapy or only irinotecan if recurrence despite dose reduction	- Discuss discontinuation of chemotherapy if recurrence despite dose reduction
Mucositis<br><br>Grade 2	- Bolus reduction of 50%	- No modification
Grade 3	-Bolus suppression, reduction of 25% of continuous 5-FU	- No modification
Grade 4	- Discuss discontinuation of chemotherapy or only irinotecan if recurrence despite dose reduction	- Discuss discontinuation of chemotherapy if recurrence despite dose reduction
Vomiting<br><br>Grade 3	- Reduction of 25% of continuous 5-FU	- Reduction of 25%
Grade 4	- Discuss discontinuation of chemotherapy or only irinotecan if recurrence despite dose reduction	- Discuss discontinuation of chemotherapy if recurrence despite dose reduction

Hand-foot syndrom<br>Grade 2<br><br>Grade 3	- Reduction of 25% of continuous 5-FU<br><br>- Bolus reduction of 50%, reduction of 50% of continuous 5-FU	- No modification<br><br>- No modification
Non hematological toxicities<br><br>Grade 3<br><br>Grade 4	 <br>- Bolus reduction of 25% and 25% of continuous 5-FU<br><br>- Discuss discontinuation of chemotherapy or only irinotecan if recurrence despite dose reduction	 <br>- Reduction de 25%<br><br>- Discuss discontinuation of chemotherapy if recurrence despite dose reduction

7.5. Management of Immune-mediated adverse event (imAE)

Guidelines for the management of immune-mediated reactions, infusion-related reactions, and non-immune-mediated reactions for durvalumab are provided in the durvalumab/tremelimumab Toxicity Management Guidelines (TMGs).

Patients should be thoroughly evaluated and appropriate efforts should be made to rule out neoplasic, infectious, metabolic, toxin or other etiologic causes of the imAE. Serologic, immunologic and histologic (biopsy) data, as appropriate, should be used to support an imAE diagnosis. In the absence of a clear alternative etiology, events should be considered potentially immune related.

All toxicities will be graded according to NCI CTCAE, Version 4.

The investigators must make every effort to differentiate chemotherapy-related side effects from those due to immunotherapy. Diarrhea/digestive toxicities could be related to 5-FU, irinotecan, durvalumab and/or tremelimumab. Then urgent GI consult and imaging and/or colonoscopy is required, as appropriate, since treatment will be different according to the drug involved in gastrointestinal toxicity. The characteristics of side effects can differentiate those due to immunotherapy from those due to chemotherapy. Diarrhea/digestive toxicities due to chemotherapy (5-FU and/or irinotecan) usually appear a few days after chemotherapy, with quick improvement with anti-diarrhea treatment.

By contrast, diarrhea/digestive toxicities due to immunotherapy usually appear weeks after immunotherapy initiation, with low efficacy of anti-diarrhea treatment. In most cases a colonoscopy with biopsies is needed to differentiate both and used appropriate treatment (immunotherapy stop and at least prednisone 1 to 2 mg/kg/day PO or IV equivalent). Most others toxicities are specific of immunotherapy and never related to chemotherapy (pulmonary, endocrine, hepatic imAEs…).

Main toxicities are listed hereinafter. Please refers also to the Toxicity Management guidelines provided appendix 8 of the protocol, if patients have other imAEs. Each updated version of the Toxicity Management Guidelines will be also provided by the sponsor.

**Gastrointestinal imAEs**		
**Severity of Diarrhea/Colitis (NCI-CTCAE v4)**	**Management**	**Follow-up**
**Grade 1**<br>Diarrhea: < 4 over baseline per day<br>Colitis: asymptomatic	No dose modifications.<br>Consider symptomatic (*e.g.* loperamide).	Monitor closely for worsening symptoms.

**Grade 2**<br>Diarrhea: 4 to 6 over baseline per day<br>Colitis: abdominal pain; mucus or blood in stool	Hold Durvalumab or Durvalumab/Tremelimumab regimen until resolution to Grade ≤1<br>• If toxicity worsens, then treat as Grade 3 or Grade 4.<br>If toxicity improves to Grade ≤1, then Durvalumab or Durvalumab/Tremelimumab regimen can be resumed after completion of steroid taper.	Consider symptomatic treatment, including hydration, electrolyte replacement, dietary changes (e.g., American Dietetic Association colitis diet), and loperamide and/or budesonide.<br>Promptly start prednisone 1 to 2 mg/kg/day PO or IV equivalent.<br>If event is not responsive within 3 to 5 days or worsens despite prednisone at 1 to 2 mg/kg/day PO or IV equivalent, GI consult should be obtained for consideration of further workup, such as imaging and/or colonoscopy, to confirm colitis and rule out perforation, and prompt treatment with IV methylprednisolone 2 to 4 mg/kg/day started.<br>If still no improvement within 3 to 5 days despite 2 to 4 mg/kg IV methylprednisolone, promptly start immunosuppressives such as infliximab at 5 mg/kg once every 2 weeks[a]. **Caution:** it is important to rule out bowel perforation and refer to infliximab label for general guidance before using infliximab.<br>Consider, as necessary, discussing with study physician if no resolution to Grade ≤1 in 3 to 4 days.<br>Once the patient is improving, gradually taper steroids over ≥28 days and consider prophylactic antibiotics, antifungals and anti-pneumocystis jiroveci Pneumonia treatment (refer to current NCCN guidelines for treatment of cancer-related infections [Category 2B recommendation]).[a]
**Grade 3 to 4**<br>Diarrhea (Grade 3): ≥7 over baseline per day<br>Colitis (Grade 3): severe abdominal pain, change in bowel habits, medical intervention indicated, peritoneal signs<br>Grade 4 diarrhea: life threatening consequences	**Grade 3**<br>Permanently discontinue study drug/study regimen for Grade 3 if toxicity does not improve to Grade ≤1 within 14 days; study drug/study regimen can be resumed after completion of steroid taper.<br><br>**Grade 4**<br>Permanently discontinue study drug/study regimen.	Promptly initiate empiric IV methylprednisolone 2 to 4 mg/kg/day or equivalent.<br>Monitor stool frequency and volume and maintain hydration.<br>Urgent GI consult and imaging and/or colonoscopy as appropriate.<br>If still no improvement within 3 to 5 days of IV methylprednisolone 2 to 4 mg/kg/day or equivalent, promptly start further immunosuppressives (e.g., infliximab at 5 mg/kg once every 2 weeks). **Caution**: Ensure GI consult to rule out bowel perforation and refer to infliximab label for general guidance before using infliximab.<br>Once the patient is improving, gradually taper steroids over ≥28 days and consider prophylactic antibiotics, antifungals, and anti-PJP treatment (refer to current NCCN guidelines for treatment of cancer-related infections [Category 2B recommendation]).[a]

[a]ASCO Educational Book 2015 "Managing Immune Checkpoint Blocking Antibody Side Effects" by Michael Postow MD.

Dermatologic imAEs		
**Grade of Rash (NCI-CTCAE v4)**	**Management**	**Follow-up**
**Any Grade**<br>(refer to NCI CTCAE v 4 for definition of severity/grade depending on type of skin rash)	**General Guidance**	**For Any Grade :**<br>− Monitor for signs and symptoms of dermatitis (rash and pruritus).<br>IF THERE IS ANY BULLOUS FORMATION, THE STUDY PHYSICIAN SHOULD BE CONTACTED **AND STUDY DRUG DISCONTINUED.**
**Grade 1**<br>Covering ≤ 10% body surface area	No dose modifications.	**For Grade 1:**<br>Consider symptomatic treatment, including oral antipruritics (e.g., diphenhydramine or hydroxyzine) and topical therapy (e.g., urea cream).

**Grade 2**<br>Covering 10-30 % body surface area	For persistent (>1 to 2 weeks) Grade 2 events, hold scheduled study drug/study regimen until resolution to Grade ≤1 or baseline.<br>• If toxicity worsens, then treat as Grade 3.<br>If toxicity improves to Grade ≤1 or baseline, then resume drug/study regimen after completion of steroid taper.	**For Grade 2:**<br>– Obtain dermatology consult.<br>– Consider symptomatic treatment, including oral antipruritics (e.g., diphenhydramine or hydroxyzine) and topical therapy (e.g., urea cream).<br>– Consider moderate-strength topical steroid.<br>– If no improvement of rash/skin lesions occurs within 3 to 5 days or is worsening despite symptomatic treatment and/or use of moderate strength topical steroid, consider, as necessary, discussing with study physician and promptly start systemic steroids such as prednisone 1 to 2 mg/kg/day PO or IV equivalent.<br>Consider skin biopsy if the event is persistent for >1 to 2 weeks or recurs.
**Grade 3 or 4**<br>Covering > 30% body surface area; life threatening consequences	**For Grade 3:**<br>Hold study drug/study regimen until resolution to Grade ≤1 or baseline.<br>If temporarily holding the study drug/study regimen does not provide improvement of the Grade 3 skin rash to Grade ≤1 or baseline within 30 days, then permanently discontinue study drug/study regimen.<br><br>**For Grade 4:**<br>Permanently discontinue study drug/study regimen.	**For Grade 3 or 4:**<br>– Dermatology advise .<br>– Promptly initiate empiric IV methylprednisolone 1 to 4 mg/kg/day or equivalent.<br>– Consider hospitalization.<br>– Monitor extent of rash [Rule of Nines].<br>– Consider skin biopsy (preferably more than 1) as clinically feasible.<br>– Once the patient is improving, gradually taper steroids over ≥28 days and consider prophylactic antibiotics, antifungals, and anti-PJP treatment (refer to current NCCN guidelines for treatment of cancer-related infections [Category 2B recommendation]).[a]<br>Consider, as necessary, discussing with study physician.

[a]ASCO Educational Book 2015 "Managing Immune Checkpoint Blocking Antibody Side Effects" by Michael Postow MD.

**Pulmonary imAEs**		
**Grade of Pneumonitis (NCI-CTCAE v4)**	**Management**	**Follow-up**
**Grade 1**<br>Radiographic changes only	No dose modifications required. However, consider holding study drug/study regimen dose as clinically appropriate and during diagnostic work-up for other etiologies.	Monitor and closely follow up in 2 to 4 days for clinical symptoms, pulse oximetry (resting and exertion), and laboratory work-up and then as clinically indicated.<br>Consider Pulmonary and Infectious disease consult.
**Grade 2**<br>Mild to moderate new symptoms	Hold study drug/study regimen dose until Grade 2 resolution to Grade ≤1.<br>• If toxicity worsens, then treat as Grade 3 or Grade 4.<br>If toxicity improves to Grade ≤1, then the decision to reinitiate study drug/study regimen will be based upon treating physician's clinical judgment and after completion of steroid taper.	Monitor symptoms daily and consider hospitalization.<br>Promptly start systemic steroids (e.g., prednisone 1 to 2 mg/kg/day PO or IV equivalent).<br>Reimage as clinically indicated.<br>If no improvement within 3 to 5 days, additional workup should be considered and prompt treatment with IV methylprednisolone 2 to 4 mg/kg/day started<br>If still no improvement within 3 to 5 days despite IV methylprednisolone at 2 to 4 mg/kg/day, promptly start immunosuppressive therapy such as TNF inhibitors (e.g., infliximab at 5 mg/kg every 2 weeks).<br>Caution: It is important to rule out sepsis and refer to infliximab label for general guidance before using infliximab.<br>Once the patient is improving, gradually taper steroids over ≥28 days and consider prophylactic antibiotics, antifungals, or anti-PJP treatment (refer to current

		NCCN guidelines for treatment of cancer-related infections [Category 2B recommendation])[a]<br>Consider pulmonary and infectious disease consult.<br>Consider, as necessary, discussing with study physician.
**Grade 3 to 4**<br>Severe new symptoms;<br>New/worsening hypoxia;<br>life-threatening	Permanently discontinue study drug/study regimen.	Promptly initiate empiric IV methylprednisolone 1 to 4 mg/kg/day or equivalent.<br>Obtain Pulmonary and Infectious disease consult; consider, as necessary, discussing with study physician.<br>Hospitalize the patient.<br>Supportive care (e.g., oxygen).<br>If no improvement within 3 to 5 days, additional workup should be considered and prompt treatment with additional immunosuppressive therapy such as TNF inhibitors (e.g., infliximab at 5 mg/kg every 2 weeks' dose) started. Caution: rule out sepsis and refer to infliximab label for general guidance before using infliximab.<br>Once the patient is improving, gradually taper steroids over ≥28 days and consider prophylactic antibiotics, antifungals, and, in particular, anti-PJP treatment (refer to current NCCN guidelines for treatment of cancer-related infections [Category 2B recommendation]).[a]

[a]ASCO Educational Book 2015 "Managing Immune Checkpoint Blocking Antibody Side Effects" by Michael Postow MD.

**Renal imAEs**		
**Grade of Liver Test Elevation (NCI-CTCAE v4)**	**Management**	**Follow-up**
**Grade 1**<br>Creatinine increased > ULN to 1.5 x ULN	No dose modifications.	Monitor serum creatinine weekly and any accompanying symptoms.<br>• If creatinine returns to baseline, resume its regular monitoring per study protocol.<br>• If creatinine worsens, depending on the severity, treat as Grade 2, 3, or 4.<br>Consider symptomatic treatment, including hydration, electrolyte replacement, and diuretics.
**Grade 2**<br>Creatinine increased > 1.5 and ≤ 3 x ULN	Hold study drug/study regimen until resolution to Grade ≤1 or baseline.<br>• If toxicity worsens, then treat as Grade 3 or 4.<br>If toxicity improves to Grade ≤1 or baseline, then resume study drug/study regimen after completion of steroid taper.	Consider symptomatic treatment, including hydration, electrolyte replacement, and diuretics.<br>Carefully monitor serum creatinine every 2 to 3 days and as clinically warranted.<br>Consult nephrologist and consider renal biopsy if clinically indicated.<br>If event is persistent (>3 to 5 days) or worsens, promptly start prednisone 1 to 2 mg/kg/day PO or IV equivalent.<br>If event is not responsive within 3 to 5 days or worsens despite prednisone at 1 to 2 mg/kg/day PO or IV equivalent, additional workup should be considered and prompt treatment with IV methylprednisolone at 2 to 4 mg/kg/day started.<br>Once the patient is improving, gradually taper steroids over ≥28 days and consider prophylactic antibiotics, antifungals, and anti-PJP treatment (refer to current NCCN guidelines for treatment of cancer-related infections [Category 2B recommendation]).[a]<br>When event returns to baseline, resume study drug/study regimen and routine serum creatinine monitoring per study protocol.
**Grade 3 to 4**<br>Creatinine increased > 6 x ULN	Permanently discontinue study drug/study regimen.	Carefully monitor serum creatinine on daily basis.<br>Consult nephrologist and consider renal biopsy if clinically indicated.<br>Promptly start prednisone 1 to 2 mg/kg/day PO or IV equivalent.

		If event is not responsive within 3 to 5 days or worsens despite prednisone at 1 to 2 mg/kg/day PO or IV equivalent, additional workup should be considered and prompt treatment with IV methylprednisolone 2 to 4 mg/kg/day started.<br>Once the patient is improving, gradually taper steroids over ≥28 days and consider prophylactic antibiotics, antifungals, and anti-PJP treatment (refer to current NCCN guidelines for treatment of cancer-related infections [Category 2B recommendation])[a]

[a]ASCO Educational Book 2015 "Managing Immune Checkpoint Blocking Antibody Side Effects" by Michael Postow MD.

**Hepatic imAEs**		
**Grade of Liver Test Elevation (NCI-CTCAE v4)**	**Management**	**Follow-up**
**Grade 1**<br>Grade 1 AST or ALT > ULN to 3.0 x ULN and/or total bilirubin > ULN to 1.5 x ULN	No dose modifications.<br>If it worsens, then treat as Grade 2 event.	Continue liver function tests monitoring per protocol.
**Grade 2**<br>AST or ALT > 3.0 to ≤ 5 x ULN and/or total bilirubin > 1.5 to ≤ 3 x ULN	Hold study drug/study regimen dose until Grade 2 resolution to Grade ≤1.<br>If toxicity worsens, then treat as Grade 3 or Grade 4.<br>If toxicity improves to Grade ≤1 or baseline, resume study drug/study regimen after completion of steroid taper.	Regular and frequent checking of LFTs (e.g., twice a week) until elevations of these are improving or resolved.<br>If no resolution to Grade ≤1 in 1 to 2 days, consider, as necessary, discussing with study physician.<br>If event is persistent (>3 to 5 days) or worsens, promptly start prednisone 1 to 2 mg/kg/day PO or IV equivalent.<br>If still no improvement within 3 to 5 days despite 1 to 2 mg/kg/day of prednisone PO or IV equivalent, consider additional work up and start prompt treatment with IV methylprednisolone 2 to 4 mg/kg/day.<br>If still no improvement within 3 to 5 days despite 2 to 4 mg/kg/day of IV methylprednisolone, promptly start immunosuppressives (i.e., mycophenolate mofetil).[a] Discuss with study physician if mycophenolate mofetil is not available. **Infliximab should NOT be used.**<br>Once the patient is improving, gradually taper steroids over ≥28 days and consider prophylactic antibiotics, antifungals, and anti-PJP treatment (refer to current NCCN guidelines for treatment of cancer-related infections [Category 2B recommendation]).[a]
**Grade 3 to 4**<br>AST or ALT > 5 x ULN and/or total bilirubin > 3 x ULN	For elevations in transaminases ≤8 × ULN, or elevations in bilirubin ≤5 × ULN:<br>Hold study drug/study regimen dose until resolution to Grade ≤1 or baseline<br>Resume study drug/study regimen if elevations downgrade to Grade ≤1 or baseline within 14 days and after completion of steroid taper.<br>Permanently discontinue study drug/study regimen if the elevations do not downgrade to Grade ≤1 or baseline within 14 days<br>For elevations in transaminases >8 × ULN or elevations in bilirubin >5 × ULN, discontinue study drug/study regimen.<br>Permanently discontinue study drug/study regimen for any case meeting Hy's law criteria (AST and/or ALT >3 × ULN + bilirubin >2 × ULN without initial findings of cholestasis	Promptly initiate empiric IV methylprednisolone at 1 to 4 mg/kg/day or equivalent.<br>If still no improvement within 3 to 5 days despite 1 to 4 mg/kg/day methylprednisolone IV or equivalent, promptly start treatment with immunosuppressive therapy (i.e., mycophenolate mofetil). Discuss with study physician if mycophenolate is not available.<br>**Infliximab should NOT be used.**<br>Perform hepatology consult, abdominal workup, and imaging as appropriate.<br>Once the patient is improving, gradually taper steroids over ≥28 days and consider prophylactic antibiotics, antifungals, and anti-PJP treatment (refer to current NCCN guidelines for treatment of cancer-related infections [Category 2B recommendation]).[a]

	(i.e., elevated ALP) and in the absence of any alternative cause.[b]<br><br>**For Grade 4:**<br>Permanently discontinue study drug/study regimen.	

[a]ASCO Educational Book 2015 "Managing Immune Checkpoint Blocking Antibody Side Effects" by Michael Postow MD.

[b]FDA Liver Guidance Document 2009 Guidance for Industry: Drug Induced Liver Injury – Premarketing Clinical Evaluation.

Cardiac imAEs		
**Myocarditis**	**Management**	**Follow-up**
**Grade 1:** asymptomatic with laboratory (e.g., BNP) or cardiac imaging abnormalities	No dose modifications required unless clinical suspicion is high, in which case hold study drug/study regimen dose during diagnostic work-up for other etiologies. If study drug/study regimen is held, resume after complete resolution to Grade 0.	Monitor and closely follow up in once or twice a week for clinical symptoms, BNP, cardiac enzymes, ECG, ECHO, pulse oximetry (resting and exertion), and laboratory work-up as clinically indicated.<br>Consider using steroids if clinical suspicion is high.
**Grade 2:** Symptoms with mild to moderate activity or exertion<br>**Grade 3:** Severe with symptoms at rest or with minimal activity or exertion; intervention indicated<br>**Grade 4:** Life-threatening consequences; urgent intervention indicated (e.g., continuous IV therapy or mechanical hemodynamic support)	**If Grade 2** -- Hold study drug/study regimen dose until resolution to Grade 0. If toxicity rapidly improves to Grade 0, then the decision to reinitiate study drug/study regimen will be based upon treating physician's clinical judgment and after completion of steroid taper. If toxicity does not rapidly improve, permanently discontinue study drug/study regimen.<br><br>**If Grade 3-4**, permanently discontinue study drug/study regimen.	Monitor symptoms daily, hospitalize.<br>Promptly start IV methylprednisolone 2 to 4 mg/kg/day or equivalent after Cardiology consultation has determined whether and when to complete diagnostic procedures including a cardiac biopsy.<br>Supportive care (e.g., oxygen).<br>If no improvement within 3 to 5 days despite IV methylprednisolone at 2 to 4 mg/kg/day, promptly start immunosuppressive therapy such as TNF inhibitors (e.g., infliximab at 5 mg/kg every 2 weeks). Caution: It is important to rule out sepsis and refer to infliximab label for general guidance before using infliximab.<br>Once the patient is improving, gradually taper steroids over ≥28 days and consider prophylactic antibiotics, antifungals, or anti-PJP treatment (refer to current NCCN guidelines for treatment of cancer-related infections [Category 2B recommendation]).[a]

[a]ASCO Educational Book 2015 "Managing Immune Checkpoint Blocking Antibody Side Effects" by Michael Postow MD.

Endocrine imAEs		
**Endocrine Disorder**	**Management**	**Follow-up**
Grade 1	No dose modifications.	Monitor patient with appropriate endocrine function tests.<br>For suspected hypophysitis/hypopituitarism, consider consultation of an endocrinologist to guide assessment of early-morning ACTH, cortisol, TSH and free T4; also consider gonadotropins, sex hormones, and prolactin levels, as well as cosyntropin stimulation test (though it may not be useful in diagnosing early secondary adrenal insufficiency).<br>If TSH < 0.5 × LLN, or TSH >2 × ULN, or consistently out of range in 2 subsequent measurements, include free T4 at subsequent cycles as clinically indicated and consider consultation of an endocrinologist.
Grade 2	For Grade 2 endocrinopathy other than hypothyroidism and Type 1 diabetes mellitus, hold study drug/study regimen dose until patient is clinically stable.<br>If toxicity worsens, then treat as Grade 3 or Grade 4.	**For Grade 2 (including those with symptomatic endocrinopathy):**<br>Consult endocrinologist to guide evaluation of endocrine function and, as indicated by suspected endocrinopathy and as clinically indicated, consider pituitary scan.<br>For all patients with abnormal endocrine work up, except those with isolated hypothyroidism or Type 1 DM, and as guided by an endocrinologist, consider short-term corticosteroids (e.g., 1 to 2 mg/kg/day

	Study drug/study regimen can be resumed once event stabilizes and after completion of steroid taper.<br>Patients with endocrinopathies who may require prolonged or continued steroid replacement (e.g., adrenal insufficiency) can be retreated with study drug/study regimen on the following conditions:<br>1. The event stabilizes and is controlled.<br>2. The patient is clinically stable as per investigator or treating physician's clinical judgement.<br>3. Doses of prednisone are ≤10 mg/day or equivalent.	methylprednisolone or IV equivalent) and prompt initiation of treatment with relevant hormone replacement (e.g., hydrocortisone, sex hormones).<br>Isolated hypothyroidism may be treated with replacement therapy, without study drug/study regimen interruption, and without corticosteroids.<br>Isolated Type 1 diabetes mellitus (DM) may be treated with appropriate diabetic therapy, without study drug/study regimen interruption, and without corticosteroids.<br>Once patients on steroids are improving, gradually taper immunosuppressive steroids (as appropriate and with guidance of endocrinologist) over ≥28 days and consider prophylactic antibiotics, antifungals, and anti-PJP treatment (refer to current NCCN guidelines for treatment of cancer-related infections [Category 2B recommendation]).[a]<br>For patients with normal endocrine workup (laboratory assessment or MRI scans), repeat laboratory assessments/MRI as clinically indicated.
Grade 3 or 4	For Grade 3 or 4 endocrinopathy other than hypothyroidism and Type 1 diabetes mellitus, hold study drug/study regimen dose until endocrinopathy symptom(s) are controlled.<br>Study drug/study regimen can be resumed once event stabilizes and after completion of steroid taper.<br>Patients with endocrinopathies who may require prolonged or continued steroid replacement (e.g., adrenal insufficiency) can be retreated with study drug/study regimen on the following conditions:<br>1. The event stabilizes and is controlled.<br>2. The patient is clinically stable as per investigator or treating physician's clinical judgement.<br>3. Doses of prednisone are ≤10 mg/day or equivalent.	Consult endocrinologist to guide evaluation of endocrine function and, as indicated by suspected endocrinopathy and as clinically indicated, consider pituitary scan.<br>Hospitalization recommended.<br>For all patients with abnormal endocrine work up, except those with isolated hypothyroidism or Type 1 DM, and as guided by an endocrinologist, promptly initiate empiric IV methylprednisolone 1 to 2 mg/kg/day or equivalent, as well as relevant hormone replacement (e.g., hydrocortisone, sex hormones).<br>For adrenal crisis, severe dehydration, hypotension, or shock, immediately initiate IV corticosteroids with mineralocorticoid activity.<br>Isolated hypothyroidism may be treated with replacement therapy, without study drug/study regimen interruption, and without corticosteroids.<br>Isolated Type 1 diabetes mellitus may be treated with appropriate diabetic therapy, without study drug/study regimen interruption, and without corticosteroids.<br>Once patients on steroids are improving, gradually taper immunosuppressive steroids (as appropriate and with guidance of endocrinologist) over ≥28 days and consider prophylactic antibiotics, antifungals, and anti-PJP treatment (refer to current NCCN guidelines for treatment of cancer-related infections [Category 2B recommendation]).[a]

[a]ASCO Educational Book 2015 "Managing Immune Checkpoint Blocking Antibody Side Effects" by Michael Postow MD.

**Neurotoxicity imAEs**		
**Neurotoxicity**	**Management**	**Follow-up**
Grade 1	No dose modifications.	Patients should be evaluated to rule out any alternative etiology (e.g., disease progression, infections, metabolic syndromes, or medications).<br>Monitor patient for general symptoms (headache, nausea, vertigo, behavior change, or weakness).<br>Consider appropriate diagnostic testing (e.g., electromyogram and nerve conduction investigations).<br>Perform symptomatic treatment with neurological consult as appropriate.
Grade 2	For acute motor neuropathies or neurotoxicity, hold study drug/study regimen dose until resolution to Grade ≤1.	Consider, as necessary, discussing with the study physician.<br>Obtain neurology consult.

	For sensory neuropathy/neuropathic pain, consider holding study drug/study regimen dose until resolution to Grade ≤1.<br>If toxicity worsens, then treat as Grade 3 or 4.<br>Study drug/study regimen can be resumed once event improves to Grade ≤1 and after completion of steroid taper.	Sensory neuropathy/neuropathic pain may be managed by appropriate medications (e.g., gabapentin or duloxetine).<br>Promptly start systemic steroids prednisone 1 to 2 mg/kg/day PO or IV equivalent.<br>If no improvement within 3 to 5 days despite 1 to 2 mg/kg/day prednisone PO or IV equivalent, consider additional workup and promptly treat with additional immunosuppressive therapy (e.g., IV immunoglobulin).
Grade 3 or 4	**For Grade 3:**<br>Hold study drug/study regimen dose until resolution to Grade ≤1.<br>Permanently discontinue study drug/study regimen if Grade 3 imAE does not resolve to Grade ≤1 within 30 days.<br><br>**For Grade 4:**<br>Permanently discontinue study drug/study regimen.	Consider, as necessary, discussing with study physician.<br>Obtain neurology consult.<br>Consider hospitalization.<br>Promptly initiate empiric IV methylprednisolone 1 to 2 mg/kg/day or equivalent.<br>If no improvement within 3 to 5 days despite IV corticosteroids, consider additional workup and promptly treat with additional immunosuppressants (e.g., IV immunoglobulin).<br>Once stable, gradually taper steroids over ≥28 days.
**Peripheral neuromotor syndromes**<br>(such as Guillain-Barre and myasthenia gravis)	**Management**	**Follow-up**
Grade 1 (Asymptomatic)	No dose modifications.	Consider, as necessary, discussing with the study physician.<br>Care should be taken to monitor patients for sentinel symptoms of a potential decompensation<br>Obtain a neurology consult.
Grade 2 (Moderate symptoms; limiting instrumental activities of daily living [ADLs])	Hold study drug/study regimen dose until resolution to Grade ≤1.<br>Permanently discontinue study drug/study regimen if it does not resolve to Grade ≤1 within 30 days or if there are signs of respiratory insufficiency or autonomic instability.	Consider, as necessary, discussing with the study physician.<br>Care should be taken to monitor patients for sentinel symptoms of a potential decompensation as described above.<br>Obtain a neurology consult<br>Sensory neuropathy/neuropathic pain may be managed by appropriate medications (e.g., gabapentin or duloxetine).<br>*MYASTHENIA GRAVIS:*<br>Steroids may be successfully used to treat myasthenia gravis. It is important to consider that steroid therapy (especially with high doses) may result in transient worsening of myasthenia and should typically be administered in a monitored setting under supervision of a neurologist.<br>Patients unable to tolerate steroids may be candidates for treatment with plasmapheresis or IV immunoglobulin (IG). Such decisions are best made in consultation with a neurologist, taking into account the unique needs of each patient.<br>If myasthenia gravis-like neurotoxicity is present, consider starting acetylcholinesterase (AChE) inhibitor therapy in addition to steroids. Such therapy, if successful, can also serve to reinforce the diagnosis.<br>*GUILLAIN-BARRE:*<br>It is important to consider here that the use of steroids as the primary treatment of Guillain-Barre is not typically considered effective.<br>Patients requiring treatment should be started with IV IG and followed by plasmapheresis if not responsive to IV IG.
**Grade 3 or 4** (Severe symptoms; limiting self care ADL or life-threatening consequences)	**For Grade 3:**<br>Hold study drug/study regimen dose until resolution to Grade ≤1.	**For Grade 3 or 4 (severe or life-threatening events):**<br>Consider, as necessary, discussing with study physician.<br>Recommend hospitalization.<br>Monitor symptoms and obtain neurological consult.

	Permanently discontinue study drug/study regimen if Grade 3 imAE does not resolve to Grade ≤1 within 30 days or if there are signs of respiratory insufficiency or autonomic instability.<br><br>**For Grade 4:**<br>Permanently discontinue study drug/study regimen.	*MYASTHENIA GRAVIS:*<br>Steroids may be successfully used to treat myasthenia gravis. They should typically be administered in a monitored setting under supervision of a neurologist.<br>Patients unable to tolerate steroids may be candidates for treatment with plasmapheresis or IV IG.<br>If myasthenia gravis-like neurotoxicity present, consider starting AChE inhibitor therapy in addition to steroids. Such therapy, if successful, can also serve to reinforce the diagnosis.<br>*GUILLAIN-BARRE:*<br>It is important to consider here that the use of steroids as the primary treatment of Guillain-Barre is not typically considered effective.<br>Patients requiring treatment should be started with IV IG and followed by plasmapheresis if not responsive to IV IG.

Others imAEs

**Myositis/Polymyositis ("Poly/myositis")**	**Management**	**Follow-up**
**Grade 1** (mild pain)	No dose modifications.	Monitor and closely follow up in 2 to 4 days for clinical symptoms and initiate evaluation as clinically indicated.<br>Consider neurology consult.<br>Consider, as necessary, discussing with the study physician.
**Grade 2**<br>(moderate pain associated with weakness; pain limiting instrumental activities of daily living [ADLs])	Hold study drug/study regimen dose until resolution to Grade ≤1.<br>Permanently discontinue study drug/study regimen if it does not resolve to Grade ≤1 within 30 days or if there are signs of respiratory insufficiency.	Monitor symptoms daily and consider hospitalization.<br>Obtain Neurology consult, and initiate evaluation.<br>Consider, as necessary, discussing with the study physician.<br>If clinical course is rapidly progressive (particularly if difficulty breathing and/or trouble swallowing), promptly start IV methylprednisolone 2 to 4 mg/kg/day systemic steroids <u>along with receiving input</u> from neurology consultant<br>If clinical course is *not* rapidly progressive, start systemic steroids (e.g., prednisone 1 to 2 mg/kg/day PO or IV equivalent); if no improvement within 3 to 5 days, continue additional work up and start treatment with IV methylprednisolone 2 to 4 mg/kg/day.<br>If after start of IV methylprednisolone at 2 to 4 mg/kg/day there is no improvement within 3 to 5 days, consider start of immunosuppressive therapy such as TNF inhibitors (e.g., infliximab at 5 mg/kg every 2 weeks). Caution: It is important to rule out sepsis and refer to infliximab label for general guidance before using infliximab.<br>Once the patient is improving, gradually taper steroids over ≥28 days and consider prophylactic antibiotics, antifungals, or anti-*Pneumocystis jirovecii* pneumonia treatment (refer to current NCCN guidelines for treatment of cancer-related infections [Category 2B recommendation]).[a]
**Grade 3 or 4**<br>(pain associated with severe weakness; limiting self-care ADLs)	**For Grade 3:**<br>Hold study drug/study regimen dose until resolution to Grade ≤1.<br>Permanently discontinue study drug/study regimen if Grade 3 imAE does not resolve to Grade ≤1 within 30 days or if there are signs of respiratory insufficiency.	Monitor symptoms closely; recommend hospitalization.<br>Obtain neurology consult, and complete full evaluation.<br>Consider, as necessary, discussing with the study physician.<br>Promptly start IV methylprednisolone 2 to 4 mg/kg/day systemic steroids <u>along with receiving input</u> from neurology consultant.

	**For Grade 4:**<br>Permanently discontinue study drug/study regimen.	If after start of IV methylprednisolone at 2 to 4 mg/kg/day there is no improvement within 3 to 5 days, consider start of immunosuppressive therapy such as TNF inhibitors (e.g., infliximab at 5 mg/kg every 2 weeks). Caution: It is important to rule out sepsis and refer to infliximab label for general guidance before using infliximab.<br>Consider whether patient may require IV IG, plasmapheresis.<br>Once the patient is improving, gradually taper steroids over ≥28 days and consider prophylactic antibiotics, antifungals, or anti-*Pneumocystis jirovecii* pneumonia treatment (refer to current NCCN guidelines for treatment of cancer-related infections [Category 2B recommendation]).[a]

[a]ASCO Educational Book 2015 "Managing Immune Checkpoint Blocking Antibody Side Effects" by Michael Postow MD.

7.6. Partial treatments stop

The treatment regimen must include at the beginning FOLFIRI plus durvalumab +/- tremelimumab, depending on treatment arm, but if severe toxicities occur some drugs can be stopped while the other drugs continued.

**Regimen allowed**			
5-FU/LV	Irinotecan	Durvalumab	Tremelimumab
**X**			
**X**		**X**	
**X**		**X**	**X**
**X**	**X**		
**X**	**X**	**X**	
		**X**	
**Regimen not allowed**			
5-FU/LV	Irinotecan	Durvalumab	Tremelimumab
	**X**		
	**X**	**X**	**X**
	**X**	**X**	
	**X**		**X**
		**X**	**X**
**X**			**X**
		**X**	**X**
**X**	**X**		**X**
			**X**

Main toxicities that will induce a partial treatment stop are:
- Immune-mediated adverse event grade 3-4 may need durvalumab +/- tremelimumab stop: FOLFIRI can be continued alone.
- Severe digestive toxicities due to chemotherapy may need irinotecan +/- 5FU stop: 5FU plus durvalumab +/- tremelimumab can be continued or durvalumab (with or without tremelimumab) alone.

7.7. Premedications, concomitant treatments and contraindicated treatments

7.7.1. Neutropenia

Treatments considered to be necessary for the patient's well-being can be administered at the investigator's discretion (anti-emetic, anti-diarrheic etc.).

The indication for primary prophylaxis with G-CSF will be at the discretion of the investigator, hematological toxicities during first-line chemotherapy and according to the patient's clinical characteristics.

In case of severe neutropenia, *i.e.* grade 3-4, patients are at high risk of febrile neutropenia and infection especially in the case of concomitant diarrhea. If these symptoms appear, dosage adjustments are planned in the next course of treatment, and the prescription of hematopoietic growth factors should be considered.

7.7.2. Contraindicated treatments (see SmPCs and IB of each protocol's molecular entities)

Durvalumab: Appendix 10 to see prohibited medications

Tremelimumab: Appendix 10 to see prohibited medications

5FU: yellow fever vaccine, attenuated live vaccine, prophylactic phenytoin. When combined with warfarin more frequent monitoring of INR

Irinotecan: drugs with St. John's wort, yellow fever vaccine.

8. LOGISTICS OF THE BIOLOGICAL STUDY (FOR PHASE II ONLY)

For patients who signed the biological informed consent, the details of the biological study (circulating DNA, stool and tumor sample) is in Appendix 2.

Samples needed

- 2 blood "cell-free DNA" tubes will be sampled

- 2 blood tubes just before the 1st treatment course
- 2 blood tubes just before the 3rd course
- 2 at progression (before the 1st course of L3)

Blood samples will be used for extracting the DNA from the plasma (circulating tumor DNA).

Sending tubes**,** via the box supplied at opening of the center:

Centre de Ressources Biologiques EPIGENETEC
Unité UMR-S 1147
45, rue des Saints-Pères - 75006 PARIS (France)
Headed by Pr. Pierre LAURENT-PUIG

Only use the box containing the UPS dispatch note **addressed to the unit INSERM UMR-S 1147**
After sending this box, the box needed at inclusion of the next patient, or for the next sample, will be sent by CRB EPIGENETEC.

In case of questions or logistic problems, contact CRB EPIGENETEC, Claire MULOT at +33 (0)1 42 86 38 61, claire.mulot@parisdescartes.fr or FFCD at +33 (0)3 80 39 34 86

- Tumor block fixed in paraffin:

Sending the pre-filled letter to your anatomopathologist:

- The anatomopatologist has to fax to the FFCD the sample sheet at the +33 (0)3 80 38 18 41

- FFCD sends a max letter for sending tumor blocks

Tumor block will be sent to:

Centre de Ressources Biologiques EPIGENETEC
Unité UMR-S 1147
45, rue des Saints-Pères - 75006 PARIS (France)
Headed by Pr. Pierre LAURENT-PUIG

In case of questions or logistic problems, contact CRB EPIGENETEC, Claire MULOT at +33 (0)1 42 86 38 61, claire.mulot@parisdescartes.fr or FFCD at +33 (0)3 80 39 34 86

- Stool sample

A document explaining in detail the sampling and shipping procedure will be provided to the patient at inclusion in the study and specific and validated material will be provided to the patient for the stool sampling and shipment.

Stool samples will be harvested:

- 1 stool sample at W0 (in the 5 days preceding the first course of treatment)
- 1 stool sample at W8 (in the 5 days preceding the first evaluation of treatment efficacy)

Sending stools, via the box supplied at opening of the center:

Laboratoire d'analyse des microbiotes,
Microbiote intestinal et immunité
Pr Harry Sokol
INSERM U1157 / UMR CNRS 7203
Université Pierre et Marie Curie
27 rue de Chaligny,
75012 Paris, France

Once received in the laboratory responsible for the analysis, the stool will be aliquoted and stored at -80°C until processing.

9. PATIENT MONITORING

9.1. During treatment

9.1.1. Before each administration of treatment

Clinical examination

- Vital signs: BP, pulse, temperature
- Weight, height and body surface area
- ECOG performance status
- Safety evaluation (toxicity precedent cycle according to NCI-CT v4.0)

Laboratory assessment:

- CBC, platelets
- Serum electrolytes (sodium, potassium, calcium, magnesium), TSH*, urea, creatinine and creatinine clearance (MDRD formula)
- Lipase, glucose
- Liver panel comprising GGT, ALP, AST, ALT, total and conjugated bilirubin
- Pregnancy test each month for women with childbearing potential

* Measures of free T3, T4 will be done if there is an abnormal TSH level or if there is a clinical suspicion of an AE related to the endocrine system.

9.1.2. Evaluation every 8 weeks until radiological progression

Patients will be evaluated **every 8 weeks** (regardless of the number of cycles received) for:

Clinical examination
- Vital signs: BP, pulse, temperature
- Weight, body Area
- ECOG performance status
- Safety evaluation (toxicity precedent cycle according to NCI-CT v4.0)
- QLQ-C30 v3.0 and STO-22 questionnaires (phase II only)

Laboratory assessment:
- CBC, platelets
- Serum electrolytes (sodium, potassium, calcium, magnesium), TSH*, urea, creatinine and creatinine clearance (MDRD formula)
- Lipase, glucose
- Liver panel comprising GGT, ALP, AST, ALT, total and conjugated bilirubin, LDH
- Blood protein, albumin and prealbumin
- CEA, CA 19.9
- Urinalysis (protein level)

* Measures of free T3, T4 will be done if there is an abnormal TSH level or if there is a clinical suspicion of an AE related to the endocrine system.

Morphological assessment:
- Thoracic-abdominal-pelvic CT (or thoracic CT and abdominal-pelvic MRI if IV contrast-enhanced CT is contraindicated) measuring tumor targets according to RECIST criteria (version 1.1, Appendix 6). Use same technique in each imaging examination as that of the initial evaluation.

<u>Phase II only:</u> For radiological progression assessments, at the physician's discretion, it is possible to continue treatment and perform a new CT-scan 4 to 8 weeks later in order to confirm progression. Send an anonymized copy of images in CD-ROM format to FFCD, 7 bd Jeanne d'Arc, BP 87900, 21079 DIJON cedex (centralized review for the secondary end point and the ancillary study)

9.2. After treatment discontinuation

9.2.1. Within 30 days for evaluating the toxicity of the last treatment

Laboratory assessment:
- CBC, platelets,
- Serum electrolytes (sodium, potassium, magnesium, calcium), TSH*, serum creatinine, creatinine clearance (MDRD)
- Lipase, glucose
- Liver panel comprising GGT, PAL, ASAT, ALAT, bilirubin (total and conjugated), LDH
- Serum albumin
- Evaluation of toxicities from the preceding cycle

* Measures of free T3, T4 will be done if there is an abnormal TSH level or if there is a clinical suspicion of an AE related to the endocrine system.

9.2.2. After premature discontinuation of treatment other than for radiological progression*

Patients will be monitored in the same way **every 8 weeks** until radiological progression:

Clinical exam (identical as 10.2.1 paragraph)
Laboratory test assessment (identical as 10.2.1 paragraph)
Evaluation of persistent toxicities (including neuropathy) up to disease progression
CT-scan: TAP (or MRI scan of abdomen-pelvis + CT-scan of thorax) with measurement of tumour targets according to RECIST v1.1 criteria

Phase II only: Send an anonymized copy of images in CD-ROM format to FFCD, 7 bd Jeanne d'Arc, BP 87900, 21079 DIJON cedex (centralized review for the secondary end point and the ancillary study)

* Clinical progression, toxicity, withdrawal of consent, patient refusal, medical decision, pregnancy or suspected pregnancy

9.2.3. After treatment discontinuation because of radiological progression

Patients will be monitored every 2 to 3 months during 1 year after treatment end or until death:
- Evaluation of persistent toxicities until 12 months after the end of treatment
- TAP-CT scan (or MRI)

10. SUBSEQUENT TREATMENTS

The 1st line treatments and subsequent treatments will be collected in the CRF. For each line, the following information will be collected:
- Type of chemotherapy,
- Number of cycles,
- Start date of chemotherapy of first chemotherapy cycle,
- End date of last chemotherapy cycle,
- Progression date.

11. MANAGEMENT OF SERIOUS ADVERSE EVENTS

Parameters for assessing safety
Safety will be assessed by evaluating the clinical and biological health status of patients during visits and by recording events that occur between visits. Toxicities will be evaluated using the NCI-CTCAE toxicity scale (version 4.0) (Appendix 8).
In case of emergency, the patient, the patient's family or the patient's physician must call the investigator to make it known that an event has occurred.

Definitions
Adverse event (AE)
An AE is an untoward medical occurrence in a person enrolled in a clinical trial, whether this occurrence is related or not to the trial itself or to the study product.
All AEs will be recorded in the CRF in the pages provided.

Serious adverse event (SAE)
An SAE is any event that meets at least one of the following criteria:
- results in death
- is life-threatening
- results in hospitalization or prolongs hospitalization
- causes permanent disability or serious temporary incapacity
- causes a congenital anomaly, fetal malformation or an abortion

- is medically significant

The terms disability and incapacity mean any temporary or permanent physical or mental disability that is clinically significant and that impacts the physical activity and/or quality of life of the patient.

A significant medical event is any clinical event or laboratory result considered to be serious by the investigator that does not meet the seriousness criteria defined above. It may put the patient at risk and require medical intervention to prevent an outcome such as one of the criteria for seriousness previously mentioned. Examples include overdose, second cancers, pregnancy and new facts that may be considered to be medically significant.

The following AE, observed with durvalumab ± tremelimumab, represent a specific interest and must be declared as SAE:

- Grade ≥ 3 diarrhea and colitis, intestinal perforation
- Pneumonitis and ILD
- Grade ≥ 3 hepatitis and transaminase increase
- Grade ≥ 3 endocrinopathies (*i.e.* events of hypophysitis/hypopituitarism, adrenal insufficiency, hyper- and hypothyroidism and type I diabetes mellitus)
- Grade ≥ 3 rash and dermatitis
- Grade ≥ 3 nephritis and blood creatinine increases
- Grade ≥ 3 pancreatitis and serum lipase and amylase increases
- Myocarditis
- Myositis / Polymyositis
- Peripheral or central neurotoxicity / neuromuscular toxicity (*e.g.* Guillain-Barré, and myasthenia gravis)
- Other inflammatory responses that are rare / less frequent with a potential immune-mediated aetiology include, pericarditis, sarcoidosis, uveitis and other events involving the eye, haematological events.

Pregnancy is a non-inclusion criterion in this trial and a reliable method of birth control must be used during the treatment and 6 months after. However, if a pregnancy occurs after a female patient’s enrollment, this patient must discontinue the trial. The sponsor should be noticed of this pregnancy with the SAE report form (no seriousness criteria must be filled). The patient will be followed until the end of the pregnancy and the outcome of the pregnancy must be reported to the sponsor. If a pregnancy occurs in partner of male patient enrolled on the trial, the sponsor should be noticed and will try, as possible, to follow the pregnancy.

Adverse effect

Any harmful, undesired reaction to a study drug regardless of the dose administered or to any investigational element. It is serious if it meets at least one of the seriousness criteria.

Unexpected SAE

An unexpected SAE is an event that is not mentioned in, or that differs in nature, intensity or outcome from, the product's reference document or SmPC.

New fact

A new fact may be an unexpected frequency of an expected SAE or an SAE related to the trial procedure, insufficient efficacy in life-threatening diseases, or clinical data.

Severity (or intensity)

Severity must not be confused with seriousness, which serves as a guide defining reporting obligations.
The severity of an event will be assessed according to the extract of the CTCAE classification (version 4.0) (Appendix 8). The severity of adverse events not listed in this classification will be assessed using the following terms:

- Mild (grade 1): does not affect the patient's routine daily activities
- Moderate (grade 2): hinders the patient's routine daily activities
- Severe (grade 3): stops the patient's routine daily activities
- Very severe (grade 4): requires resuscitative action/endangers the patient's life
- Death (grade 5)

Causal relationship
Related: an event is said to be "related" when a causal relationship between the event and the product being studied may reasonably be suspected
Unrelated: an event is said to be "unrelated" when a causal relationship between the event and the product being studied cannot reasonably be suspected
Doubtful: the causal relationship is said to be "doubtful" if there are doubts about the causal relationship between the event and the product being studied. The relationship cannot be positively ruled out or confirmed.

Sponsor's responsibilities
As soon as the sponsor receives the SAE report made by the investigator, he has to assess the causal relationship between the SAE and the study product(s).
If the investigator and/or sponsor considered the SAE as related to one of the study products, it is therefore a serious adverse effect, and the sponsor must determine whether the effect is expected or unexpected.
If it is an unexpected serious adverse effect or a new fact, the sponsor drafts an initial report which is sent to the ANSM, IRB and EMA (via EudraVigilance) without delay (in the event of death or of a life-threatening situation) or within 15 days (in other cases).
If it is an expected serious adverse effect, it is compiled for the purpose of drafting the annual safety reports.

Events that must not be considered serious
Disease progression must not be considered as a SAE.
Events that may be related to progression but may also have been caused by the treatment still need to be reported, for example thromboembolic events, hemorrhage, or perforation.
Because of the seriousness of the disease in this study, certain conditions defined as SAEs will be excluded from the SAE reporting procedure. These comprise:

- Hospitalization or surgery that are specifically connected with treating the disease. However, hospitalization or the prolonging of hospitalization due to one of the study products must be reported as SAEs.
- Hospitalization to simplify the study treatments or procedures.

The reference documents in this trial will be:
For 5-FU: FLUOROURACILE EBEWE® SmPC
For Folinic acid: ELVORINE® SmPC
For Irinotecan: CAMPTO® SmPC
For Durvalumab: section 5.4 of Investigator's Brochure v12 (November 2017)
For Tremelimumab: section 5.4 of Investigator's Brochure v8 (November 2017)

The versions of the SmPCs that we will use to define expectedness or unexpectedness will be those in effect at the time of analysis.

Procedure
The investigator reports all SAEs to the sponsor, whether expected or unexpected, and whether related to the trial or not, that occur during the study or within 90 days of the last administration of treatment.
Any late SAEs (occurring after this 90-day period) that are reasonably related to the study drugs or to the trial must be reported regardless of when they occur.
The report is filed by faxing the "Serious Adverse Event Report" form (Appendix 11), dated, signed and documented as soon as possible, within 24 working hours of the event being observed to the **FFCD data center on +33 (0)3 80 38 18 41.**

The investigator must follow the patient until the event resolves or stabilizes or until the patient dies. It may sometimes require a follow-up of the patient after the trial discontinuation.

The investigator sends additional information to the sponsor using the SAE report form, ticking the "follow-up" box and increasing the number of the report to highlight that it is a follow-up report and not an initial report. These follow-up reports must be sent within 24 hours of receiving the information. The investigator also sends the last follow-up report when the SAE has resolved or stabilized.
The investigator handles requests for additional information to document the initial observation.

12. STATISTICAL ANALYSIS

12.1. Safety analyses (safety run-in phase)

For safety run-in phases, patients will be treated in 5 expert centers with a huge experience in the use of immune checkpoints inhibitors.

1st step: In order to check the good tolerability of FOLFIRI plus durvalumab combination, 5 patients will be treated by FOLFIRI (irinotecan 180mg/m²) plus durvalumab (1500 mg) in 5 expert centers. The inclusion will be stopped at 5 patients. When the 5th patient will have received 2 cycles of treatment, the safety analysis will be done with all the safety data available at this date. The review will be done by an Independent Data Monitoring Committee (IDMC).

The decision of IDMC and the data available will be sent to ANSM. We will wait the ANSM approval to re-open the inclusion of patients.

2nd step: 3 patients per arm will be randomized to receive either FOLFIRI (irinotecan 180 mg/m²) plus durvalumab (1500 mg) or FOLFIRI (**irinotecan 150 mg/m²**) plus durvalumab (1500 mg) plus tremelimumab (75 mg). These 6 patients will be treated in the same 5 expert centers. When the 6th patient will have received 2 cycles of treatment, the safety analysis will be done with all the safety data available at this date (for the 11 patients included in these safety run-in phases). The review will be done by an Independent Data Monitoring Committee (IDMC).

The decision of IDMC and the data available will be sent to ANSM. We will wait the ANSM approval to open the phase II trial.

12.2. Endpoints for phase II study

12.2.1. Primary efficacy endpoint

The primary endpoint is the percentage of patients alive and without radiological progression (according to RECIST 1.1) at 4 months after randomization according to investigator.

12.2.2. Secondary endpoints

The secondary endpoints are:

Progression free survival (PFS) median:
Is defined as the time between date of randomization and date of the first radiological progression (according to RECIST 1.1) or death (from any cause), whichever occurs first. Patients alive without progression will be censored at date of last news.

Overall Survival (OS):
Is defined as the time between date of randomization and date of death (from any cause). Patients alive will be censored at date of last news.

Time to progression (TTP):
Is defined as the time between date of randomization and the date of first radiological progression (according to RECIST v1.1). Patients without progression will be censored at date of last news or date of death. The death will not be considered as an event.

Best Objective Response rate (BRR):
Is defined as complete or partial response at the best response evaluation during the treatment according to RECIST v1.1.

Disease control rate (DCR) at each timepoint:

Is defined as complete or partial response or stable disease at the best response evaluation according to RECIST v1.1.

Time to strategy failure:
Is defined as the time between randomization date and date of death (from any cause) or the date of first radiological progression in the FOLFIRI + durvalumab arm or date of the second radiological progression after re-introduction of tremelimumab in the FOLFIRI plus durvalumab plus tremelimumab arm or date of definitive discontinuation.
In case a treatment is stopped for toxicity reason but re-introduced later for progression, then this progression will not be considered for this endpoint.

Safety profile
Toxicities will be graded according to the NCI-CTCAE v4.0 classifications.

Quality of life (QoL)
Is evaluated using EORTC QLQ-C30 and the STO22 questionnaires.

Centralized evaluation of PD-L1 expression
All efficacy endpoints (OS, PFS, TTP, BRR and DCR) will be evaluated according to the expression of PD-L1.

Centralized radiological assessments of RECIST v1.1 response and iRECIST response according Seymour *et al.* criteria (22). For exploration, secondary endpoints (OS, PFS, TTP, BRR and DCR) will be analysed according to this centralized review.

12.3. Sample size justification, statistical hypothesis

There is no statistical hypothesis for the safety run-in phase. A total of 11 patients will be included in the 2 steps of the safety run-in phase before the randomized phase II study will begin.

Median PFS with FOLFIRI as second-line chemotherapy in gastric and GEJ adenocarcinoma is between 2 to 4 months (10, 13, 23-25). We expect at least a 5 months median PFS with FOLFIRI + durvalumab ± tremelimumab which is clinically significant.

<u>The hypotheses for the randomized phase II are:</u>
- H_0: 50% of patients alive and without progression at 4 months is not acceptable.
- H_1: 70% of patients alive and without progression at 4 months is expected.

With a risk α (one-sided) of 5%, a power of 85% and according to the binomial exact method (A'Hern) (26), 44 evaluable patients (i.e. patients randomized and with at least one dose of products taken) are needed by arm. Assuming 5% of non-evaluable or lost to follow-up patients, **47 patients will be included by arm (94 patients in total).**

Taking into account the 11 patients included in the safety run-in phase, 105 patients will be included in the trial.

<u>Rules for selection to be applied to both experimental arms (on the 44 evaluable patients):</u>
if 28 or more patients are alive without progression at 4 months then the arm will be considered as efficient.

In case both arms will conclude to efficacy, safety data will be analyzed (both Adverse events and Serious Adverse events) in order to see if one has a better safety profile. One or both arms could be compared to a control arm (FOLFIRI) in a phase III study.

12.4. Statistical analysis for phase II study

A detailed Statistical Analysis Plan (SAP) will be written before the database lock.

12.4.1. Population definition

Intent-to-treat (ITT) population: all randomized patients, whatever their eligibility and whatever treatment they have received. Patients will be analyzed in the treatment arm allocated at randomization.

Modified Intent-to-treat (mITT) population: ITT patients who have taken at least one dose of products (whatever the dose is and whatever the treatment is).

Safety population (SP): ITT patients who have received at least one dose of treatment. The patients will be analysed in the treatment arm really received.

12.4.2. Endpoint evaluation

Evaluation of baseline characteristics (ITT)
All baseline characteristics will be described using descriptive statistics and will be presented by treatment arm and on the overall population.
The continuous variables are described by the usual statistics as mean (with standard deviation), median (with interval Inter-quartiles) and min-max. The categorical variables will be described using patient numbers and percentages.

Evaluation of the primary endpoint (mITT)
For the primary endpoint, frequencies and percentage will be given for each treatment arms. A one-sided 95% confidence interval of each percentage will also be given.

Evaluation of secondary endpoints (ITT)
For Survival analyses (OS, PFS, TTP and Time to strategy failure), the Kaplan-Meier method (27) will be used to estimate median and curves will be plotted. The median and the rates at different times will be described with their 95% confidence interval.
The median follow-up time will be calculated using the "reverse Kaplan-Meier" method (28).

Evaluation of safety (SP)
A safety analysis will be performed on the first 10 patients included in the study. Patient enrolment will be stopped and toxicities data on these 10 first patients will be harvested.
Adverse events (toxicity events) will be described for each treatment arm, by number of patients by preferred term (PT) within each SOCs (System Organ Classes). Each toxicity will be analyzed with on the maximum grade according to the NCI-CTC v4 during the treatment phase. They will also be described by grouping the maximum grade 1-2 versus 3-4-5.
A Serious Adverse Event (SAE) report will be provided by pharmacovigilance.

Evaluation of quality of life (ITT)
Quality of life will be evaluated using the EORTC QLQ-C30 + STO-22 questionnaires.
The scores will be described at baseline by treatment arms.
The time to definitive deterioration of the global health score will be calculated: it is defined as the time between the date of randomization and the date of first deterioration by more than five points on the global health scale in comparison with the score at baseline (without any subsequent improvement). Patients alive or who died without deterioration will be censored at date of last news.

13. STUDY COMMITTEES

13.1. Independent data monitoring committee

An independent data monitoring committee (IDMC) will be established that comprises at least two digestive oncologists, a statistician or methodologist, and an expert in pharmacovigilance. IDMC members will be selected by the Sponsor and will be independent from the study.
The IDMC will be convened at the end of each step of safety run-in phase as described in the protocol. Then, throughout the duration of the study the IDMC will meet at least once a year, or more often if the sponsor deems it is necessary in light of pharmacovigilance signal. The IDMC may also be convened at any time during the trial whenever the sponsor considers there to be a need to do so.
The committee will issue decisions on all safety data sent to the sponsor by the centers (AEs and/or SAEs). It will evaluate all patients included in the trial in the 2 months preceding the date of its meeting.
The IDMC can recommend that the clinical study be stopped early if there is strong evidence that the investigational medicinal products are harming patients. The committee can make recommendations regarding modification of the study if there is strong evidence that such change would substantially contribute to the well-being of patients.

13.2. Steering committee

A steering committee will be set up. The chairperson of the steering committee will be the study coordinator. This committee will also comprise the co-coordinators, the FFCD study project manager, an FFCD statistician, and the chairman of the biological research committee. Its functions will include, among other things, issuing decisions on the management of the research, such as amendments or, if needed, early trial closure. The steering committee will meet as often as required throughout the study. It will make the necessary decisions concerning substantial protocol amendments, trial closure or trial extension.

13.3. Medical review

A medical review committee will be set up to improve the quality of clinical data collected. If there is a discrepancy between the data provided by the investigator and those provided by the medical review committee, data management will seek clarification from the investigator.

13.4. Biological research committee

A biological research committee will be established whose role is to answer questions about sample-taking and storage as well as the organization of sample analysis. The committee will meet regularly and report its proposals to the steering committee. This committee will comprise, among others, the study coordinator and a biologist. The committee chairman will be Pr Pierre Laurent-Puig.

14. BACKGROUND INFORMATION AND RATIONALE FOR THE TRIAL

Gastric adenocarcinoma is the fourth most frequent cancer and the second leading cause of cancer mortality (1). Advanced gastric adenocarcinoma has a poor prognosis with short overall survival (ranging from 10% to 15% at 5-years) even after surgical complete resection and despite the progress in therapeutic approaches. Most of the patients have metastatic, locally advanced or recurrent unresectable disease. So, systemic treatment remains an important issue especially since chemotherapy improves survival and quality of life (compared to best supportive care alone). First-line chemotherapy depends on HER2 status, which also influenced overall survival (14 months for HER2 positive versus 10 months for HER2 negative tumors). In HER2 negative tumors standard first-line regimen is a doublet of fluoropyrimidine (5-fluorouracil or capecitabine) plus a platinum salt (cisplatin or oxaliplatin) (2). 5-fluorouracil (5-FU) and capecitabine as also cisplatin and oxaliplatin have similar efficacy but different toxicities (3, 4).

In patients whose tumor overexpresses the HER2 receptor adding trastuzumab to fluoropyrimidine/cisplatin regimen increased overall survival compared to chemotherapy alone (5). In HER2 negative tumors the addition of docetaxel to cisplatin/fluoropyrimidine regimen increased overall survival (6) but its use remains limited in clinical practice because of its high toxicity. Preliminary results demonstrated a high efficacy with less toxicities of docetaxel-oxaliplatin-fluoropyrimidine combination, also called TFOX/FLOT regimen (7). Indeed, in France a large phase III trial comparing TFOX versus FOLFOX in first-line treatment of patients with advanced gastric or gastro-oesophageal junction adenocarcinoma is ongoing (GASTFOX, trial NCT03006432). Primary endpoint is progression-free survival (PFS) and 506 patients are planned between 2017 and 2020 (actually at the date of January 30, 2018, 65 patients are included).

Second-line chemotherapy improves overall survival (OS) as compared to best supportive care alone in patients with an acceptable general condition (performance status 0-2). Indeed, with docetaxel monotherapy there was a significant difference in overall survival for the chemotherapy arm with a median of 5.2 versus 3.6 months in best supportive care alone arm (HR=0.67, p=0.01) (8). Weekly paclitaxel monotherapy is also used because of its good efficacy-toxicity ratio (9). Irinotecan monotherapy also significantly improves overall survival compared to supportive care alone in a phase III study (4.0 versus 2.4 months; HR=0.48, 95%CI 0.25-0.92; p=0.012) (10). Recently ramucirumab monotherapy demonstrated its efficacy on overall survival in a randomized, placebo-controlled second-line metastatic study (11). In a randomized phase 3 trial ramucirumab also showed its efficacy in combination with paclitaxel versus paclitaxel monotherapy with a median overall survival of 9.6 versus 7.4 months, respectively (p=0.017; HR=0.81) (12). However, the “amelioration du service medical rendu” (ASMR) assessed by the French “Haute Autorité de Santé” (HAS) consider an insufficient benefit to a reimbursement of ramucirumab in France. The HAS gave a moderate ASMR opinion (ASMR IV).

Docetaxel is more and more frequently used in first-line chemotherapy then in this setting taxane (alone or combined with others drugs) cannot be used as second-line regimen. Indeed, based on a phase III trial FOLFIRI (5-FU plus irinotecan) is one most used regimen in second-line in European countries, especially in France (13). FFCD 0307 trial, a phase III comparing FOLFIRI-ECX (epirubicin-cisplatin-capecitabine) to the reverse sequence (ECX-FOLFIRI), showed that both sequences are possible.

Human tumors tend to activate the immune system regulatory checkpoints as a means of escaping immunosurveillance. For instance, interaction between PD1 (Program Death 1) and PD-L1 (Program Death 1 ligand) will lead the activated T cell to a state of anergy. PD-L1 is up regulated on a wide range of cancers. Anti-PD1 and anti-PD-L1 monoclonal antibodies (mAbs), called immune checkpoint inhibitors (ICIs), have consequently been designed to restore T cell activity. Others ICIs are investigated, notably cytotoxic T-lymphocyte-associated protein 4 (CTLA-4) inhibitors. CTLA-4 transmits an inhibitory signal to T cells to prevent early excessive T cell activation. CTLA4 blockade may stimulate a more robust antitumor response by sustaining activation and proliferation of T lymphocytes and may overcome immune suppression mediated by regulatory T cells. ICIs have been recently tested in many cancers with promising results, especially in tumors with microsatellite instability (MSI) and/or PD-L1 overexpression.

Preliminary results in metastatic gastric cancer with anti-PD1 mAbs are highly promising. In a trial with pembrolizumab, only PD-L1 positive tumors were eligible to the treatment with a cut off at 1% (14). Thirty-nine patients were enrolled and 67% had received at least two prior chemotherapy regimens. The overall response rate was 22%. The median PFS and OS were 1.9 months and 11.4 months, respectively. KEYNOTE-059 Phase 2 multicohort study with pembrolizumab monotherapy in advanced gastric cancer treatment has been presented at ASCO 2017 meeting (15). Among 259 patients included in the trial response rate was 11.6%. OS was 5.6 months. Response rates were 15.5% in PDL1+ tumors versus 6.4% in PDL1- tumors and 57.1% in MSI tumors versus 9% in MSS tumors. Up until now, overlap between microsatellite instability and PD-L1 expression is unknown in gastric cancer. An anti-PD-L1 mAb (avelumab) was evaluated in a phase Ib expansion study (n=20, Japanese patients), with 15% of objective response rate and 11.9 weeks for progression-free survival. A second cohort with avelumab included 55 patients for maintenance therapy after first-line chemotherapy, with 7.3% of objective response rate and 14 weeks of PFS (16). Phase I/II CheckMate-032 evaluated nivolumab (anti-PD-1) ± ipilimumab

(anti-CTLA4) at different doses in advanced gastric cancer (17). The overall response rate was between 8% to 24% and the median OS between 4.8 to 6.9 months according to treatment arm.

Others anti-PD1/anti-PD-L1/anti-CTLA4 mAbs are also currently under investigation in gastric cancer alone or in combination with chemotherapy. Nevertheless, up until now there is no published data concerning ICI plus chemotherapy in gastric cancer. Finally, immunogenic cell death induced by chemotherapy may enhance efficacy of ICIs (18). Durvalumab (MEDI4736) is a human monoclonal antibody directed against PD-L1 in development for the treatment of many cancers (19). A phase I study included 16 patients with advanced gastric cancer and the objective response rate was 25% (20). Tremelimumab is a fully human monoclonal antibody against CTLA-4. Durvalumab plus tremelimumab combination showed a manageable tolerability profile, with antitumour activity irrespective of PD-L1 status in non-small cell lung cancer (NSCLC) (21). Durvalumab alone or combined with tremelimumab is evaluated in phase III studies in NSCLC (e.g NEPTUNE and MYSTIC), small cell lung cancer (CASPIAN), hepatocellular carcinoma (HIMALAYA), bladder cancer (DANUBE) and head and neck cancer (EAGLE and KESTREL).

Concerning safety of anti-PD1 plus anti-CTLA4 combination, in the randomized phase I/II CheckMate-032 study, that included 160 patients, there was no unexpected toxicity signal. Grade 3 and 4 treatment-related adverse events were 17%, 47%, and 27%, respectively (17). These rates of grade 3 and 4 treatment-related adverse events are those usually found with the anti-PD1 plus anti-CTLA4 combination in other tumors, observed approximately in 40% of patients. Up until now, there is no published data concerning combination of ICIs plus irinotecan. Nevertheless, in all trial combining chemotherapy plus anti-PD1 and/or anti-CTLA4 chemotherapy drugs were used at full-dose (5FU, oxaliplatin, cisplatin…). An Italian trial just started and combined full-dose FOLFOXIRI (5-FU 3200 mg/m^2 plus irinotecan 165 mg/m^2 and oxaliplatin 85 mg/m^2) with bevacizumab (5 mg/kg) and atezolizumab (anti-PD-L1, 840 mg) in metastatic colorectal cancers as first-line treatment. FOLFOXIRI is a triplet chemotherapy more "toxic" than FOLFIRI doublet chemotherapy and this trial is a randomized phase II (FOLFOXIRI plus bevacizumab and atezolizumab versus FOLFOXIRI plus bevacizumab). There is, however, a preliminary safety phase in 6 patients, once they have all received at least 2 cycles of treatment, the latter being administered at full dose (AtezoTRIBE trial, NCT03721653).

The present randomized multicentric non-comparative phase II study aimed to assess the rate of patients alive and without progression at 4 months with advanced gastric or gastro-oesophageal junction (GEJ) adenocarcinoma, pre-treated with fluoropyrimidine + platinum +/- taxane, with two arms Folfiri plus durvalumab versus Folfiri plus durvalumab plus tremelimumab. Indeed, most patients in the French multicentric first-line GASTFOX trial (506 patients planned between 2017 and 2020) can be included in the second-line setting in the DURIGAST trial. Due to the lack of data concerning Folfiri plus durvalumab plus tremelimumab combination, a safety run-in phase will be performed at the beginning of the DURIGAST trial.

15. REFERENCES

1. Jemal A, Bray F, Center MM, Ferlay J, Ward E, Forman D. Global cancer statistics. CA Cancer J Clin 2011;61:69-90.
2. Kang YK, Kang WK, Shin DB, Chen J, Xiong J, Wang J et al. Capecitabine/cisplatin versus 5-fluorouracil/cisplatin as first-line therapy in patients with advanced gastric cancer: a randomised phase III noninferiority trial. Ann Oncol 2009;20:666-73.
3. Cunningham D, Starling N, Rao S, Iveson T, Nicolson M, Coxon F et al; Upper Gastrointestinal Clinical Studies Group of the National Cancer Research Institute of the United Kingdom. Capecitabine and oxaliplatin for advanced esophagogastric cancer. N Engl J Med 2008;358:36-46.
4. Al-Batran SE, Hartmann JT, Probst S, Schmalenberg H, Hollerbach S, Hofheinz R et al. Phase III trial in metastatic gastroesophageal adenocarcinoma with fluorouracil, leucovorin plus either oxaliplatin or cisplatin. J Clin Oncol 2008;26:1435-42.

5. Bang YJ, Van Cutsem E, Feyereislova A, Chung HC, Shen L, Sawaki A et al. Trastuzumab in combination with chemotherapy versus chemotherapy alone for treatment of HER2-positive advanced gastric or gastro-oesophageal junction cancer (ToGA) : a phase 3, openlabel, randomised controlled trial. Lancet 2010;376:687-97.
6. Van Cutsem E, Moiseyenko VM, Tjulandin S, Majlis A, Constenla M, Boni C et al; V325 Study Group. Phase III study of docetaxel and cisplatin plus fluorouracil compared with cisplatin and fluorouracil as first-line therapy for advanced gastric cancer. J Clin Oncol 2006;24:4991-7.
7. Van Cutsem E, Boni C, Tabernero J, Massuti B, Middleton G, Dane F et al. Docetaxel plus oxaliplatin with or without fluorouracil or capecitabine in metastatic or locally recurrent gastric cancer. Ann Oncol 2015;26:149-56.
8. Ford HE, Marshall A, Bridgewater JA, Janowitz T, Coxon FY, Wadsley J et al. Docetaxel versus active symptom control for refractory oesophagogastric adenocarcinoma (COUGAR-02): an openlabel, phase 3 randomised controlled trial. Lancet Oncol 2014;15:78-86.
9. Hironaka S, Ueda S, Yasui H, Nishina T, Tsuda M, Tsumura T et al. Randomized, open-label, phase III study comparing irinotecan with paclitaxel in patients with advanced gastric cancer without severe peritoneal metastasis after failure of prior combination chemotherapy using fluoropyrimidine plus platinum: WJOG 4007 trial. J Clin Oncol 2013;31:4438-44.
10. Thuss-Patience PC, Kretzschmar A, Bichev D, Deist T, Hinke A, Breithaupt K et al. Survival advantage for irinotecan versus best supportive care as second-line chemotherapy in gastric cancer- A randomised phase III study of the Arbeitsgemeinschaft internistische onkologie (AIO). Eur J Cancer 2011;47:2306-14.
11. Fuchs CS, Tomasek J, Yang CJ, Dumitru F, Passalacqua R, Goswami C et al. Ramucirumab monotherapy for previously treated advanced gastric or gastro-oesophageal junction adenocarcinoma (REGARD): an international, randomised, multicentre, placebo-controlled, phase 3 trial. Lancet 2014;383:31-9.
12. Wilke H, K Muro, Van Cutsem E, Oh SC, Bodoky G, Shimada Y et al. Ramucirumab plus paclitaxel versus placebo plus paclitaxel I patients with previously treated advanced gastric or gastro-oesophageal junction adenocarcinoma (RAINBOW): a double-blind, randomized phase 3 trial. Lancet Oncol 2014;15:1224-35.
13. Guimbaud R, Louvet C, Ries P, Ychou M, Maillard E, André T et al. Prospective, randomized, multicenter, phase III study of fluorouracil, leucovorin, and irinotecan versus epirubicin, cisplatin, and capecitabine in advanced gastric adenocarcinoma: a French intergroup (Fédération Francophone de Cancérologie Digestive, Fédération Nationale des Centres de Lutte Contre le Cancer and Groupe Coopérateur Multidisciplinaire en Oncologie) study. J Clin Oncol 2014;32:3520-6.
14. Muro K, Chung HC, Shankaran V, Geva R, Catenacci D, Gupta S et al. Pembrolizumab for patients with PD-L1-positive advanced gastric cancer (KEYNOTE-012): a multicentre, open-label, phase 1b trial. Lancet Oncol 2016;17:717-26.
15. Fuchs CS, Doi T, Woo-Jun Jang R, Muro K, Satoh T, Machado M et al. KEYNOTE-059 cohort 1: Efficacy and safety of pembrolizumab (pembro) monotherapy in patients with previously treated advanced gastric cancer. J Clin Oncol 2017;35 (suppl; abstr 4003).
16. Yamada Y, Nishina T, Iwasa S, Shitara K, Muro K, Esaki T et al. A phase I dose expansion trial of avelumab (MSB0010718C), an anti-PD-L1 antibody, in Japanese patients with advanced gastric cancer. J Clin Oncol 2015;33 (suppl; abstr 4047).
17. Janjigian YY, Ott PA, Calvo E, Kim JW, Ascierto PA, Sharma P et al. Nivolumab ± ipilimumab in pts with advanced (adv)/metastatic chemotherapy-refractory (CTx-R) gastric (G), esophageal (E), or gastroesophageal junction (GEJ) cancer: CheckMate 032 study. J Clin Oncol 2017;35 (suppl; abstr 4014).
18. Massard C, Gordon MS, Sharma S, Rafii S, Wainberg ZA, Luke J et al. Safety and Efficacy of Durvalumab (MEDI4736), an Anti-Programmed Cell Death Ligand-1 Immune Checkpoint Inhibitor, in Patients With Advanced Urothelial Bladder Cancer. J Clin Oncol 2016;34:3119-25.
19. Zitvogel L, Kepp O, Senovilla L, Menger L, Chaput N, Kroemer G. Immunogenic tumor cell death for optimal anticancer therapy: the calreticulin exposure pathway. Clin Cancer Res 2010;16:3100-4.
20. Segal NH, Antonia SJ, Brahmer JR, Maio M, Blake-Haskins A, Li X et al. Preliminary data from a multi-arm expansion study of MEDI4736, an anti-PD-L1 antibody. J Clin Oncol 2014;32 (suppl; abstr 3002).
21. Antonia S, Goldberg SB, Balmanoukian A, Chaft JE, Sanborn RE, Gupta A, et al. Safety and antitumour activity of durvalumab plus tremelimumab in non-small cell lung cancer: a multicentre, phase 1b study. Lancet Oncol. 2016;17(3):299-308.

22. Seymour L, Bogaerts J, Perrone A, Ford R, Schwartz LH, Mandrekar S et al; RECIST working group. iRECIST: guidelines for response criteria for use in trials testing immunotherapeutics. Lancet Oncol 2017;18:e143-e152.
23. Samalin E, Afchain P, Thézenas S, Abbas F, Romano O, Guimbaud R et al. Efficacy of irinotecan in combination with 5-fluorouracil (FOLFIRI) for metastatic gastric or gastroesophageal junction adenocarcinomas (MGA) treatment. Clin Res Hepatol Gastroenterol 2011;35:48-54.
24. Maugeri-Saccà M, Pizzuti L, Sergi D, Barba M, Belli F, Fattoruso S et al. FOLFIRI as a second-line therapy in patients with docetaxel-pretreated gastric cancer: a historical cohort. J Exp Clin Cancer Res 2013;32:67.
25. Kim SH, Lee GW, Go SI, Cho SH, Kim HJ, Kim HG et al. A phase II study of irinotecan, continuous 5-fluorouracil, and leucovorin (FOLFIRI) combination chemotherapy for patients with recurrent or metastatic gastric cancer previously treated with a fluoropyrimidine-based regimen. Am J Clin Oncol 2010;33:572-6.
26. A'Hern R. P., Sample size tables for exact single-stage phase II designs. Statistics in medicine 2001; 20:859 - 866
27. Kaplan EL and Meier P. Nonparametric Estimation from Incomplete Observation. Journal of the American Statistical Association 1958;53:457-481
28. Schemper M and Smith TL. "A Note on Quantifying Follow-up in Studies of Failure Time". Control Clin Trials 1996;17:343-6.

16.ADMINISTRATIVE CONSIDERATIONS

TRIAL SPONSOR

The trial sponsor is the FFCD. The trial is registered under the EudraCT number 2018-002014-13.

REMINDER CONCERNING APPLICABLE REGULATIONS

This trial will be conducted in accordance with current French law, with the ethical principles of the Helsinki Declaration of 1964 and its subsequent revisions, with Good Clinical Practice of the International Conference on Harmonization (ICH–E6, 17/07/96), with European directive 2001/20/EC on the conduct of clinical trials, with the Huriet Act as amended (20/12/88) on the protection of persons participating in biomedical research, and with the provisions laid down by the CNIL, the French data protection agency (Act no. 94-548 of 01/07/94 completing Act no. 78-17 of 06/01/78).

PUBLIC LIABILITY INSURANCE

An insurance policy was taken out by the sponsor on 29/11/2018 under the number 137.681, in accordance with article L 1121-10 of the French Public Health Code (Appendix 13).

APPLICATION FOR APPROVAL FROM THE IRB AND ANSM

This protocol received approval from IRB CPP NORD OUEST II on 16/04/2019 (Appendix 14).
This protocol received authorization from the ANSM on 27/11/2018 (Appendix 15).

COLLECTING PATIENT CONSENT

The investigator undertakes to provide the patient with information and to collect written clinical and biological consent from the patient (using the information sheets and consent forms in appendices 1 and 2) before registering the patient in the study. A copy of these consent forms must be retained by the investigator for 15 years so that they may be shown to the regulatory authorities in the event of inspection. The originals must be given back to the patient.

In accordance with the recommendations of the French cancer action plan (measure 5.1), this document was submitted to the Patient Advisory Board for Clinical Research of the French League Against Cancer.

NOTIFICATION OF HOSPITAL SENIOR MANAGEMENT AND CLINICAL TRIAL AGREEMENT

Before the trial is launched, the sponsor will inform the senior management of the hospitals of the utility for the investigator of taking part in this trial.
A clinical trial agreement (which includes no additional costs) will be drawn up between the administrator of the investigator center and the sponsor.

IMMUNOTHERAPY PATIENT CARD

The investigator can give an “immunotherapy patient card” once the patient randomization performed. This card will inform that the patient is being treated by immunotherapy and will follow the patient throughout the clinical study. This is useful especially if the patient must be treated urgently in another center.

DATA ARCHIVING

Files will remain confidential and may be consulted only under the authority of the physicians treating the patients.
The sponsor and health authorities will have direct access to these documents in the event of an inspection.
The investigator will retain the study documents for 15 years after the end of the trial.

COMPUTERIZED ARCHIVING

In accordance with the provisions of French Data Protection Act no. 78-17 of January 6, 1978, as amended by the Act of August 9, 2004, trial data will be recorded in a computerized database of the Randomization, Management and Analysis Center of the FFCD, with the exception of items relating to patient identity.

DATA PROCESSING

The Randomization, Management and Analysis Center of the FFCD will be responsible for managing and analyzing data.

MONITORING, QUALITY ASSURANCE AND INSPECTIONS BY THE AUTHORITIES

The investigator hereby accepts that the files of patients enrolled may be consulted by any person appointed by the sponsor and/or by the health authorities to carry out an audit. Visits to inspect the files on site, which are scheduled with the investigator's agreement, may be made during or after the trial inclusion period.
This protocol will be monitored by traveling CRAs of the FFCD.

17.RULES FOR PUBLICATION

They will comply with those laid down by the PRODIGE research group (Appendix 12).

18.APPENDICES

APPENDIX 1: CLINICAL AND BIOLOGICAL INFORMED CONSENT

APPENDIX 2: BIOLOGICAL STUDIES

An ancillary study of biological samples (blood), tumour samples (primary tumours) and stools has been set up in order to look for factors predictive of treatment response and prognostic factors.

The principal objective is to generate hypotheses enabling to determine future biomarkers predictive of response to immune checkpoint inhibitors, in particular levels of circulating tumour DNA, immunohistochemistry (IHC) on the tumour (PD-L1, PD-L2, CD8 and others immune markers), mutational load, gastric molecular sub-group and intestinal microbiota.

Molecular and IHC analyses planned have been defined based on current knowledge and may change over time depending on new findings. This will be decided by the DURIGAST biological study steering committee.

1/ RATIONALE

Circulating tumour DNA

Up until now, molecular analysis of tumour cells was performed using samples of cancerous tissue. Improvement in molecular biology techniques now makes it possible to detect, to extract and to analyse circulating DNA. Circulating free DNA exists in healthy subjects at concentrations of approximately 0 to 100 ng/ml (1). In cancer patients, concentrations range from 0 to 5,000 ng/ml. Existence of free DNA in the blood of cancer patients that is a carrier of specific alterations, in fact has been demonstrated in many studies. This tumour DNA, which presents the same molecular signature as the tumour, is released by tumour cells when they enter into apoptosis or undergo necrosis (1,2). It is possible to detect cancer-specific mutations in the peripheral blood of patients with metastatic cancer. This then specifically involves circulating tumour DNA (ctDNA). The kinetic follow-up study of ctDNA may make it possible to monitor the evolution of the tumour, determine the efficacy of treatment and detect possible recurrences, as well as the emergence of tumour subclones of resistance to targeted treatments (3,4). Furthermore, preliminary data suggest that ctDNA may predict non-response to treatment even before radiological progression. To date, there are no data on the dynamic evolution of ctDNA in patients treated with an immune checkpoint inhibitor in gastric cancer. Therefore, ctDNA represents a marker of tumour burden which is easily detectable and relevant to analyse in the DURIGAST trial.

Factors predictive of response to immune checkpoint inhibitors

Currently, molecular biology technical laboratories have high output sequencing techniques enabling to analyse a mutational panel prognostic and/or predictive of response to treatment, as example *RAS* and *BRAF* mutations for metastatic colorectal cancer. In the DURIGAST study, blocks of tumour tissue collected enable analysis of a large mutational panel determined based on advances in knowledge on the carcinogenesis of gastric cancer (MSI, HER2, PIK3CA…) as well as gastric molecular sub-group. Gastric cancers were divided into four subtypes: tumours positive for Epstein–Barr virus (PIK3CA mutations, extreme DNA hypermethylation, and amplification of JAK2, PD-L1 and PD-L2); microsatellite unstable tumours (elevated mutation rates); genomically stable tumours (enriched for the diffuse histological variant); and tumours with chromosomal instability (aneuploidy and focal amplification of receptor tyrosine kinases).

The expression of PD-1 or of PD-L1 has been studied to predict efficacy of immune checkpoint inhibitors (ICI), but remains controversial with thresholds for positivity that have not been precisely determined. Even if expression of PD-L1 seems to be correlated with clinical efficacy, objective responses have been observed in PD-L1 negative tumours. Furthermore, a uniform definition of a PD-L1 positive tumour is needed, in fact the threshold for a positive response ranges between 1 and 50% depending on studies and the expression of PD-L1 can be analysed either in tumour cells or in immune cell infiltration of tumours (5). The predictive value of expression of PD-L1 and of other biomarkers remains to be evaluated in gastric cancer treated with ICI, in particular with durvalumab and tremelimumab. Furthermore, recently other markers (PD-1, PD-L2) or T-lymphocyte populations (CD8, CD4, CD3, FoxP3) separately or in the setting of a combined immunoscore may influence response to ICI. Different immune scores and immune markers will be evaluated in the DURIGAST study in order to compare them alone or in combination with each other.

Recent studies suggest that mutational load, which is related to the number of potentially immunogenic tumour antigens, affects the efficacy of ICI (6). Therefore, a high mutational load is an essential prior condition for efficacy of ICI. New technics of molecular biology now make it possible to determine precisely this mutational load and represent a potentially major biomarker of efficacy of ICI and will be performed in the DURIGAST trial.

Rational for Microbiome Analysis

Some studies suggest that the gut microbiota might be involved in the efficacy and toxicity of chemotherapies and immunotherapies (7-9). A study lead in Gustave Roussy hospital showed intestinal microbiota

modulates the anticancer immune effects of cyclophosphamide. Indeed, cyclophosphamide alters the microbiota composition and induces translocation of a type of Gram+ bacteria into secondary lymphoid organs. There, these bacteria stimulate immune response by generation of pTh17 antitumoral cells. Experience on mice's microbiota without these bacteria showed a reduction of pTh17 generation and resistance to cyclophosphamide, whereas transfer of these bacteria into mices lead to cyclophosphamide efficacy.

Besides, nivolumab and ipilimumab can increase immune response and can also induce immune-related adverse effects as diarrheas or enterocolitis. The inflammatory mechanism of these effects suggests an important role of the gut microbiota (10).

The main objective of this part of the study is to evaluate the influence of gut microbiota composition on toxicity and efficacy of Folfiri plus durvalumab ± tremelimumab in patients included in this clinical trial. For this purpose, fecal microbiota composition will be analyzed before initiating the treatment (W0) and at week 8 (W8, first evaluation) and will be correlated to treatment toxicity and efficacy.

A document explaining in detail the sampling and shipping procedure will be provided to the patient at inclusion in the study and specific and validated material will be provided to the patient for the stool sampling and shipment. The patient will be contacted (phone, email, text message) few days before the planned sampling to remind him what should be done. The sampling day (W0 and W8), the patient will have to fill a simple clinical information file that will be sent with the stool sample. Once received in the laboratory responsible (Laboratory for microbiota analysis, Gut Microbiota and Immunity lab (Pr Harry Sokol), INSERM U1157 / UMR CNRS 7203, Université Pierre et Marie Curie, 27 rue de Chaligny, 75012 Paris, France) for the analysis, the stool will be aliquoted and stored at -80°C until processing.

The fecal microbiota composition will be assessed by sequencing the small-subunit (16S) ribosomal RNA gene of bacterial communities. Indeed, all the bacteria have the 16S rRNA gene, which is characterized by enough sequence conservation allowing accurate alignment and enough variation allowing phylogenetic analyses. The recent advances and decrease cost of deep sequencing offers the possibility to characterize the complete gut microbiota, in our case using MiSeq Illumina technology.

Following DNA extraction with a validated method (11) and sequencing, sequences will be analyzed as described previously (12, 13). Briefly, the sequences will be processed in a data curation pipeline, which remove sequences from the analysis if they are less than 200 nucleotides or greater than 600 nucleotides, have a low read quality score, contain ambiguous characters, or have a non-exact barcode match. Remaining sequences will be assigned to samples based on barcode matches, and barcode and primer sequences will be then trimmed. Chimeric sequences will be identified and removed, and reads classified using Greengenes ribosomal RNA database. Sequences will be also aligned and clustered (Qiime, http://qiime.org/). Microbiota data will be analyzed by a combination of supervised and unsupervised modeling strategies. Briefly, this will include: implementation of ecology-derived diversity and similarity indices to derive correlation models with studied genotype or clinical status. Linear discriminant analysis effect size (LEfSe) and multivariate analysis by linear models (MaAsLin) will be used to identify components of the microbiota associated with clinical status. Hierarchical clustering and Principal Component Analysis will be also performed. All the comparisons between groups of interest will be performed at Phylum, class, order, family, genus and species level.

The analysis will particularly focus on: (1) looking for microbiota factors associated with treatment toxicity and (2) efficacy.

2/ PRACTICAL MODALITIES

Necessary samples

For patients who signed the biological study informed consent form, the following samples will be collected:

- Blood samples: test tubes will be used for extraction of DNA from plasma (circulating tumour DNA) and will be sent to EPIGENETEC.

- For measurement of ctDNA: 2 test tubes of cell free DNA blood will be collected before the 1st course of CT, the 3rd course and at disease progression (before the 1st course of L3).

- Tumour block fixed in paraffin: tumour block will be used to detect several molecular markers and will be sent to EPIGENTEC.

- Stool sample: stool samples will be used for 16S rRNA detection of microbiota bacteria and will be sent to the Laboratory for microbiota analysis, Gut Microbiota and Immunity lab (Pr Harry Sokol), INSERM U1157 / UMR CNRS 7203, Université Pierre et Marie Curie, 27 rue de Chaligny, 75012 Paris, France.

Conduct of analyses :
1/ **Blood samples**:
- Measurement of ctDNA:
    * total concentration of circulating DNA
    * ctDNA concentration (Garrigou et al. Clin Chem 2016, Pecuchet Clin Chem 2016)

2/ A block of tumour tissue embedded in paraffin:
- Extraction of DNA for:
    * microsatellite instability
    * gastric molecular subgroup
    * tumour mutation load
- Construction of the *Tissue Micro Array* block for immunohistochemistry:
    * expression of immune checkpoints (PD-L1, PD-L2)
    * immune response/immune scores

3/ **Stool samples**:
- Extraction of 16S rRNA:
    * identification of bacteria composing the intestinal microbiota of patients

Bibliography:

1. Schwarzenbach H, Hoon DS, Pantel K. Cell-free nucleic acids as biomarkers in cancer patients. Nat Rev Cancer. 2011;11:426-37
2. Stroun M, Lyautey J, Lederrey C, et al. About the possible origin and mechanism of circulating DNA apoptosis and active DNA release. Clin Chim Acta. 2001;313:139-42
3. Misale S, Yaeger R, Hobor S, et al. Emergence of KRAS mutations and acquired resistance to anti-EGFR therapy in colorectal cancer. Nature. 2012;486:532-6
4. Diaz LA Jr, Williams RT, Wu J, et al. The molecular evolution of acquired resistance to targeted EGFR blockade in colorectal cancers. Nature. 2012;486:537-40.
5. Granier C, Roussel H, de Guillebon E, et al. Biomarkers from the tumor microenvironment to predict clinical response to checkpoint inhibitors. J OncoPathology 2014.
6. Vogelstein B, Papadopoulos N, Velculescu VE et al. Cancer genome landscapes. Science 2013;339:1546-58.
7. Viaud S, Saccheri F, Mignot G, et al. The intestinal microbiota modulates the anticancer immune effects of cyclophosphamide. Science 2013;342:971-6.
8. Gopalakrishnan V, Spencer CN, Nezi L, et al. Gut microbiome modulates response to anti-PD-1 immunotherapy in melanoma patients. Science 2018;359:97-103.
9. Routy B, Le Chatelier E, Derosa L, et al. Gut microbiome influences efficacy of PD-1-based immunotherapy against epithelial tumors. Science 2018;359:91-97.
10. Carbonnel F, Soularue E, Coutzac C, et al. Inflammatory bowel disease and cancer response due to anti-CTLA-4: is it in the flora? Semin Immunopathol 2017;39:327-31.
11. Qin J, Li R, Raes J, et al. A human gut microbial gene catalogue established by metagenomic sequencing. Nature 2010;464:59-65.
12. Lamas B, Richard ML, Leducq V, et al. CARD9 impacts colitis by altering gut microbiota metabolism of tryptophan into aryl hydrocarbon receptor ligands. Nat Med 2016;22:598-605.
13. Sokol H, Leducq V, Aschard H, et al. Fungal microbiota dysbiosis in IBD. Gut. 2017;66:1039-1048

APPENDIX 3: QUALITY OF LIFE – QLQ-C30

EORTC QLQ-C30 (version 3.0) – PRODIGE 59 - DURIGAST

We are interested in some things about you and your health. Please answer all of the questions yourself by **circling the number** that best applies to you. There are no "right" or "wrong" answers. The information that you provide will remain strictly confidential.

Please fill in: **The first three letters of your surname:** ααα

The first two letters of your first name: αα

Your date of birth: βδβδβχχδ

Today's date: βδβδβχχδ

	Not at all	A little	Quite a bit	Very much
1. Do you have any trouble doing strenuous activities, like carrying a heavy shopping bag or suitcase?	1	2	3	4
2. Do you have any trouble taking a long walk?	1	2	3	4
3. Do you have any trouble taking a short walk outside the house?	1	2	3	4
4. Do you need to stay in bed or in a chair during the day?	1	2	3	4
5 Do you need help with eating, dressing, washing yourself or using the toilet?	1	2	3	4

During the past week:

	Not at all	A little	Quite a bit	Very much
6. Were you limited in doing either your work or other daily activities?	1	2	3	4
7. Were you limited in pursuing your hobbies or other leisure time activities?	1	2	3	4
8. Were you short of breath?	1	2	3	4
9. Have you had pain?	1	2	3	4
10. Did you need to rest?	1	2	3	4
11. Have you had trouble sleeping?	1	2	3	4
12. Have you felt weak?	1	2	3	4
13. Have you lacked appetite?	1	2	3	4
14. Have you felt nauseated?	1	2	3	4
15. Have you vomited?	1	2	3	4
16. Have you been constipated?	1	2	3	4
17. Have you had diarrhea?	1	2	3	4
18. Were you tired?	1	2	3	4
19. Did pain interfere with your daily activities?	1	2	3	4
20. Have you had difficulty in concentrating on things, like reading a newspaper or watching television?	1	2	3	4
21. Did you feel tense?	1	2	3	4
22. Did you worry?	1	2	3	4
23. Did you feel irritable?	1	2	3	4
24. Did you feel depressed?	1	2	3	4
25. Have you had difficulty remembering things?	1	2	3	4
26. Has your physical condition or medical treatment interfered with the family life?	1	2	3	4
27. Has your physical condition or medical treatment interfered with your social activities?	1	2	3	4
28. Has your physical condition or medical treatment caused you financial difficulties?	1	2	3	4

For the following questions, please circle the number between 1 and 7 that best applies to you

29. How would you rate your overall health during the last week?

1	2	3	4	5	6	7
Very poor						Excellent

30. How would you rate your overall quality of life during the last week?

1	2	3	4	5	6	7
Very poor						Excellent

© Copyright 1999 EORTC Quality of Life Study Group, version 3.0. All rights reserved. English version

The data collected will be analyzed by computer. In accordance with the law "Informatique et Liberté" of January 6, 1978 amended by law 2004-801 of August 6, 2004 relating to the automated processing of health data, you may exercise a right of access and modification through your investigator.

APPENDIX 4: QUALITY OF LIFE - STO-22

Patients sometimes report that they have the following symptoms or problems. Please indicate the extent to which you have experienced these symptoms or problems during the past week. Please answer by circling the number that best applies to you.

During the past week:

	Not al all	A little	Quite a bit	Very Much
31. Have you had problems eating solid foods?	1	2	3	4
32. Have you had problems eating liquidised or soft foods?	1	2	3	4
33. Have you had problems drinking liquids?	1	2	3	4
34. Have you had discomfort when eating?	1	2	3	4
35. Have you had pain in your stomach area?	1	2	3	4
36. Have you had discomfort in your stomach area?	1	2	3	4
37. Did you have a bloated feeling in your abdomen?	1	2	3	4
38. Have you had trouble with acid or bile coming into your mouth?	1	2	3	4
39. Have you had acid indigestion or heartburn?	1	2	3	4
40. Have you had trouble with belching?	1	2	3	4
41. Have you felt full up too quickly after beginning to eat?	1	2	3	4
42. Have you had trouble enjoying your meals?	1	2	3	4
43. Has it taken you a long time to complete your meals?	1	2	3	4
44. Have you had a dry mouth?	1	2	3	4
45. Did food and drink taste different from usual?	1	2	3	4
46. Have you had trouble with eating in front of other people ?	1	2	3	4
47. Have you been thinking about your illness?	1	2	3	4
48. Have you worried about your weight being too low?	1	2	3	4
49. Have you felt physically less attractive as a result of your disease or treatment?	1	2	3	4
50. Have you worried about your health in the future?	1	2	3	4
51. Have you lost any hair?	1	2	3	4
52. Answer this question only if you lost any hair:<br>If so, were you upset by the loss of your hair?	1	2	3	4

©QLQ-TO22 Copyright 1999 EORTC Group on Quality of life. All rights reserved. ENGLISH

The data collected will be analyzed by computer. In accordance with the law "Informatique et Liberté" of January 6, 1978 amended by law 2004-801 of August 6, 2004 relating to the automated processing of health data, you may exercise a right of access and modification through your investigator.

APPENDIX 5: ECOG PERFORMANCE STATUS – CALCULATION OF CLEARANCE

<u>**ECOG Performance Status**</u>

0= Fully active, able to carry on all predisease performance without restriction
1= Restricted in physically strenuous activity but ambulatory and able to carry out work of a light or sedentary nature
2= Ambulatory and capable of all selfcare but unable to carry out any work activities. Up and about more than 50% of waking hours
3= Capable of only limited selfcare, confined to bed or chair more than 50% of waking hours
4= Completely disabled. Cannot carry on any selfcare. Totally confined to bed or chair
5= Dead

<u>**CLEARANCE**</u>

MDRD (*Modification of the Diet in Renal Disease*) formula (Levey, 2000):

186.3 × (creatinine (in mmol/L)/88.4) × 1154 age-0203 (x 0.742 if female x 1.21 if black skin)

APPENDIX 6 : RECIST CRITERIA, VERSION 1.1

"New response evaluation criteria in solid tumours: Revised RECIST guideline (version 1.1)" E.A. Eisenhauer, P. Therasse, J. Bogaerts, L.H. Schwartz, D. Sargent, R. Ford, J. Dancey, S. Arbuck, S. Gwyther, M. Mooney, L. Rubinstein, L. Shankar, L. Dodd, R. Kaplan, D. Lacombe, J. Verweij;
Eur J Cancer, 4 5 (2 0 0 9) 2 2 8 –2 4 7.

Lesions on inclusion:
Lesions and lymph nodes are classed individually as being measurable or non-measurable.

Measurable disease
A lesion is measurable if it can be accurately measured in at least one dimension (longest diameter in the plane of measurement is to be reported).
To be measurable, lesions must have a minimum size of
≥ 10 mm on CT (CT scan slice thickness no greater than 5 mm)
≥ 10 mm on clinical examination (measured using a caliper); lesions that cannot be accurately measured with calipers should be classed as non-measurable
20 mm on chest X-ray
For a malignant lymph node to be considered pathological and measurable, its short axis must measure ≥ 15 mm (the short axis is the axis perpendicular to the largest dimension of the lymph node). Only the length of this short axis is reported on inclusion and during follow-up.

Non-measurable disease
All other lesions, including small lesions (longest diameter < 10 mm on CT or lymph nodes with ≥ 10 mm and < 15 mm short axis) as well as truly non-measurable lesions: leptomeningeal disease, ascites, pleural or pericardial effusion, inflammatory breast disease, lymphangitic involvement of skin or lung, abdominal masses identified by physical examination but unconfirmed by imaging techniques, and cystic lesions.
NB: bone lesions, simple cystic lesions and lesions that have received prior local treatment require special consideration (see comments below).

Target lesions
Target lesions are selected from the measurable lesions presented by the patient on entry into the study. **A maximum of five target lesions are selected, with no more than two target lesions per organ.** Target lesions will be selected so as to be representative of all involved organs. The largest lesions (in the longest dimension) that may be repeatedly and reproducibly measured throughout the trial using the initial examination method are to be chosen. Lymph nodes may be considered as target lesions if their short axis as measured on CT is ≥ 15 mm.

The sum of the diameters of these target lesions (the longest diameter for lesions, and short axis for lymph nodes) is what is followed throughout the trial for assessing response or progression.

Non-target lesions
All other lesions are identified as non-target lesions and are also recorded on inclusion. They are not measured but they are followed throughout the trial.

Criteria for response to treatment:
Target lesions:

Complete response (CR) Disappearance of all lesions, and all lymph nodes (whether target or non-target) must have reduction in *short axis* to < 10 mm.

Note: lymph nodes selected as target lesions must always be measured (in the same anatomical plane as the baseline examination), even if they decrease in size during the study to a short axis of < 10 mm. Therefore, when lymph nodes are used as target lesions, the sum of the lesions' dimensions is not necessarily zero even if there is CR since a normal lymph node is defined as having a short axis < 10 mm. To qualify for CR, every lymph node must achieve a short axis of < 10 mm.

Partial response (PR) At least a 30% decrease in the sum of diameters of target lesions, taking as reference the baseline sum diameters.

Progressive disease (PD) At least a 20% increase in the sum of diameters of target lesions, taking as reference the smallest sum, or nadir, in the study (this includes the baseline sum if that is the smallest in the study). In addition to the relative increase of 20%, the sum must also demonstrate an absolute increase of at least 5 mm.
Note: the appearance of one or more new lesions is also considered progression.
However, if there is progression compared with the nadir *and* response compared with the baseline examination, this is considered progression.

Stable disease (SD) Neither PR, CR or PD.

<u>Non-target lesions</u>

CR Disappearance of all non-target lesions and normalization of tumor markers. All lymph nodes must have reached a short axis of < 10 mm.
Non-CR/SD Persistence of one or more non-target lesions and/or tumor marker levels above the normal limits.
PD **<u>Unequivocal</u>** increase in size of existing non-target lesions or appearance of one or more new lesions.

Overall response:

Target lesions	Non-target lesions	New lesion		Overall response
CR	CR	No	=	**CR**
CR	Non-CR/Non-PD	No	=	**PR**
CR	Not assessed	No	=	**PR**
PR	Non-PD or not all assessed	No	=	**PR**
SD	Non-PD or not all assessed	No	=	**SD**
Not all assessed	Non-PD	No	=	**Unassessable**
PD	Any	Yes or no	=	**PD**
Any	PD	Yes or no	=	**PD**
Any	Any	Yes	=	**PD**

Comments on lesion measurability on entry
Bone lesions:
Bone scan, PET scan or plain films are not considered adequate imaging techniques to measure bone lesions. However, these techniques can be used to confirm the presence or disappearance of bone lesions.
Lytic bone lesions or mixed lytic-blastic lesions, with identifiable soft tissue components, can be considered as measurable lesions if they can be evaluated by cross-sectional imaging techniques such as CT or MRI and if the soft tissue component meets the definition of measurability described above.
Cystic lesions:
Lesions that meet the criteria for radiographically defined simple cysts are not considered as malignant lesions (be it measurable or non-measurable).
Cystic lesions thought to represent cystic metastases can be considered as measurable lesions if they meet the definition of measurability described above. However, if noncystic lesions are present in the same patient, these are preferred for selection as target lesions.
Lesions with prior local treatment:
Tumor lesions situated in a previously irradiated area, or in an area subjected to other loco-regional therapy, are usually not considered measurable unless there has been demonstrated progression in the lesion since the local therapy. Study protocols should detail the conditions under which such lesions may be considered measurable.

APPENDIX 7: IRECIST CRITERIA OF IMMUNOLOGICAL RESPONSE

iRECIST immunological response criteria

(Bibliographical reference: Seymour and al Lancet Oncol. 2017 Mar;18(3):e143-e152. doi: 10.1016/S1470-2045(17)30074-8. Epub 2017 Mar 2. iRECIST: guidelines for response criteria for use in trials testing immunotherapeutics.)

Identification of lesions at inclusion:

Lesions and lymph nodes will be classified individually as measurable or not measurable.

- Measurable disease

In order for a lesion to be considered measurable, at least one of its dimensions must be able to be measured precisely (the longest dimension, in the plan of measurement, should be recorded).
To be measurable, lesions should present a minimum measurement of:
- ≥ 10 mm in a CT-scan (so that the width of the band of the CT-scan is at most 5 mm)
- ≥ 10 mm by the clinical examination (measurable with a sliding calliper) (lesions which cannot be measured precisely should be classified as non-measurable)
- 20 mm per X-ray of the chest (or of the thorax)
- For a malignant lymph node to be considered as pathological and measurable, the latter should have a smallest axis ≥ 15 mm (the smallest axis being perpendicular to the largest dimension of the node). Only the length of the smallest axis will be recorded both at inclusion, as well as during follow-up.

- Non-measurable disease

All other lesions including small lesions (largest diameter ≤ 10 mm in a CT-scan or lymph nodes whose smallest axis is ≥ 10 mm and < 15 mm) as well as actually non-measurable lesions: leptomeningeal disease, ascites, pleurisy, pericarditis, inflammatory breast disease, pulmonary or cutaneous carcinomatous lymphangitis, abdominal or pelvic masses detected by clinical examination but not confirmed by imaging and cystic lesions.
NB: bone lesions, simple cystic lesions and lesions which previously underwent local treatment require special consideration (see comments below).

Classification of lesions
Lesions and nodes then will be classified as target or non-target lesions:

- Target lesions

Target lesions are selected from among measurable lesions which the patient presents at time of entry in the study. **5 target lesions at most are selected in total with a maximum of 2 target lesions per organ.** Selection of target lesions will be performed in order to be representative of all organs invaded by choosing the largest lesions (in their largest dimension) which, in addition, may be followed throughout the trial with the method used in the initial examination. The largest lesions are not necessarily the best measurable targets to follow. The most representative lesions and moreover the easiest to find from one examination to another are to be preferred, but are not necessarily the largest. Lymph nodes can be considered as target lesions if their smallest axis (measured on CT-scan) is ≥ 15 mm. If possible, avoid necrotic lesions.

It is the sum total of diameters of these target lesions (largest axis for lesions and smallest axis for nodes) which will be followed throughout the trial to evaluate response or progression.

- Non-target lesions

All other lesions are identified as non-target lesions and are also recorded at inclusion. They are not measured, but are followed throughout the trial.

Comments relating to the measurability of lesions at time of admission

- *Bone lesions:*

Imaging by bone scinti-scan, PET-scan and plain X-ray films are not considered as adequate for measurement of bone lesions. However, these techniques can be used to confirm the existence or disappearance of bone lesions.
Lytic or mixed lytic-osteoblastic bone lesions, which contain a soft tissue identifiable component can be considered as measurable lesions in so far as they may be measured by cross-sectional techniques such as CT or MRI scan, and that the soft tissue component satisfies conditions for measurability indicated in the above.

- *Cystic lesions:*

Lesions which correspond to the X-ray diagnosis of a simple cyst are not considered as malignant lesions (neither measurable, nor non-measurable)
Malignant cystic lesions can be taken into account as a measurable lesion insofar as they satisfy criteria for measurability defined in the above. However, if the patient presents with other non-cystic lesions, the latter will be chosen preferably as the target lesion.

- *Lesions previously treated locally:*

Lesions located in a previously radiated area or which received another loco-regional therapy generally are not considered measurable, except for lesions which have progressed since local treatment. The study protocol should detail the specific conditions enabling to consider such lesions as measurable.

Criteria of response to treatment:

Evaluation of target lesions:

Complete response (iCR: immune complete response): Disappearance of all lesions. Furthermore, all lymph nodes (target or non-target), should have reached a dimension < 10 mm in their smallest axis. No confirmation is necessary.
Warning: nodes selected as target lesions should always be measured (dimension of the smallest axis in the anatomical plane used for BASELINE examination), even if they decrease in size during the study and that their smallest axis becomes < 10 mm. From then, when the nodes are used as a target lesion, the "sum total" of dimensions of lesions is not necessarily nil, even in case of a complete response, since a normal lymph node is defined as having its smallest axis < 10 mm. In order to obtain a complete response, each node should have reached a dimension of < 10 mm in its smallest axis.

Partial response (iPR: immune partial response): Decrease by at least 30% in sum total of diameters of target lesions compared to initial sum total of diameters (BASELINE examination).

Non-confirmed progression (iUPD: immune unconfirmed progressive disease): Increase ≥ 20% of sum total of diameters of target lesions compared to the smallest total of diameters observed in the study (NADIR), including baseline visit. In addition to this, relative increase of 20%, this sum total should increase by at least 5 mm.
Comment: unconfirmed progression does not result in discontinuation of treatment.

Confirmed progression (iCPD: immune confirmed progressive disease): An additional increase ≥ 5 mm of sum total of diameters of target lesions

Stabilisation (iSD: immune stability of disease): Neither iPR (or iCR), nor progression.

Evaluation of non-target lesions

Complete response (iCR: immune complete response): Disappearance of all non-target lesions and normalisation of tumour markers. All lymph nodes should have reached a smallest diameter < 10 mm.

Incomplete response – Stabilisation (non-iCR – non-iUPD): Persistence of at least one non-target lesion and/or one tumour marker above normal.

Unconfirmed progression (iUPD: immune unconfirmed progressive disease): an i**ndisputable** increase in size of non-target lesions observed at the first evaluation or after an evaluation which concluded in a complete response or stabilisation of these non-target lesions.
Comment: unconfirmed progression does not result in discontinuation of treatment

Confirmed progression (iCPD: immune confirmed progressive disease):
An additional **indisputable** increase in size of non-target lesions following an evaluation which concluded iUPD for these non-target lesions.

Evaluation of new lesions (NL):

Initial occurrence of one or more new lesions is a progression which should be confirmed 4 to 8 weeks later. The new lesion(s) is/are to be categorised as measurable or not measurable and should be followed independently of target and non-target lesions at baseline. These new lesions are to be categorised:

- As measurable new lesions: at most 5 new lesions (of which maximum 2 per organ) will be measured and followed, but should not be included in the sum total of lesions identified at the baseline examination.
- As non-measurable new lesions: all other new lesions are identified as non-target lesions and are followed in the following evaluations.

An evaluation of these new lesions satisfies rules established by RECIST 1.1 (see following table)

Measurable NL	Non-measurable NL	Occurrence of NL		Overall response NL
CR	CR	No	**=**	**CR**
CR	Non-CR/Non-PD	No	=	PR
CR	Not evaluated	No	=	PR
PR	Non-PD or not all evaluated	No	=	PR
SD	Non-PD or not all evaluated	No	=	SD
Not all evaluated	Non-PD	No	=	Not evaluable (NE)
PD*	Indifferent	Yes or no	=	PD
Indifferent	PD**	Yes or no	=	PD
Indifferent	Indifferent	Yes	=	PD

*For measurable new lesions, progression is observed if an increase of at least 5 mm.
**For non-measurable new lesions, progression is observed if an increase is indisputable.

Overall response:

The overall response is the conjunction of target lesions, non-target lesions, new lesions; each type of lesion should be considered first individually and then in a combined manner for overall response.

Following observation of an unconfirmed progression (iUPD), in the following evaluation (4 to 8 weeks later), the overall response will be:

- Confirmed progression (iCPD), if observation of a further new progression of measurable target lesions and/or of non-target lesions and/or of new lesions
- Re-initialisation of response in other cases (iCR, iPR or iSD)

In this case, a new cycle iUPD and then iCPD will be necessary in order to conclude in failure of therapy because of disease progression. This rule applies for all categories of lesions followed (target lesions, non-target lesions and new lesions).

This concept makes it possible to take into account typical responses in immunotherapy, i.e. responses to postponement which occurred after pseudo-progression.

Allocation of overall response according to iRECIST criteria:

If no non-target lesion is followed, the overall response is based on the result of response of target lesions, new lesions and overall response in previous evaluations.

Evaluation at time t			Overall response in previous evaluation (t-1)	Overall response at time t
**Target lesion Response**	**Non-target lesion response**	**Overall response of New lesions**		
iCR	iCR	Absence or CR	Indifferent	iCR
iCR	iCR	PR or NE	Indifferent	iPR
iCR	iCR	SD	Indifferent	iSD
iCR	Neither iCR – nor iUPD	Absence or CR	Indifferent	iPR
iCR	Neither iCR – nor iUPD	PR or NE	Indifferent	iPR
iCR	Neither iCR – nor iUPD	SD	Indifferent	iSD
iPR	Neither iCR – nor iUPD	Absence or CR or PR or NE	Indifferent	iPR
iPR	Neither iCR – nor iUPD	SD	Indifferent	iSD
iSD	Neither iCR – nor iUPD	Absence or non-PD	Indifferent	iSD
iUPD	Neither iCR – nor iUPD	Absence or non-PD	Neither iUPD/iCPD	iUPD
Neither iUPD/iCPD	iUPD	Absence or non-PD	Neither iUPD/iCPD	iUPD
iUPD	Neither iUPD/iCPD	Absence	iUPD – observed solely for target lesions	iUPD
Neither iUPD/iCPD	iUPD	Absence	iUPD observed solely for non-target lesions	iUPD
iUPD	iUPD	Absence	Neither iUPD/iCPD	iUPD
iUPD	iUPD	Absence	iUPD – observed for target lesions and non-target lesions	iUPD
Non-iCPD	Non-iCPD	1st occurrence	Neither iUPD/iCPD	iUPD
indifferent	indifferent	1st occurrence	iUPD – observed solely for target lesions and/or non-target lesions	iCPD
iUPD	indifferent	indifferent	iUPD - observed for non-target lesions and/or occurrence of new lesions	iCPD
indifferent	iUPD	indifferent	iUPD - observed for target lesions and/or occurrence of new lesions	iCPD
indifferent	indifferent	PD	Indifferent	iCPD
iCPD	indifferent	indifferent	Indifferent	iCPD
indifferent	iCPD	indifferent	Indifferent	iCPD

APPENDIX 8: ASSESSMENT OF TOXICITIES

ASSESSMENT OF TOXICITIES NCI-CTC V4.0

http://evs.nci.nih.gov/ftp1/CTCAE/About.html

and then click « Files Data » on « CTCAE 4.03 2010-06-14.xls »

TOXICITY MANAGEMENT GUIDELINES

This document describing the management of toxicity related to immunotherapy is provided hereinafter. Each new updated version will be provided by the sponsor to the center.

Dosing Modification and Toxicity Management Guidelines for Immune-Mediated, Infusion-Related, and Non-Immune–Mediated Reactions (MEDI4736 Monotherapy or Combination Therapy With Tremelimumab or Tremelimumab Monotherapy) 1 November 2017 Version

General Considerations

Dose Modifications	Toxicity Management
Drug administration modifications of study drug/study regimen will be made to manage potential immune-related AEs based on severity of treatment-emergent toxicities graded per NCI CTCAE v4.03.<br>In addition to the criteria for permanent discontinuation of study drug/study regimen based on CTC grade/severity (table below), permanently discontinue study drug/study regimen for the following conditions:<br>• Inability to reduce corticosteroid to a dose of ≤10 mg of prednisone per day (or equivalent) **within 12 weeks** after last dose of study drug/study regimen<br>• Recurrence of a previously experienced Grade 3 treatment-related AE following resumption of dosing<br>**Grade 1** No dose modification<br>**Grade 2** Hold study drug/study regimen dose until Grade 2 resolution to Grade ≤1.<br>If toxicity worsens, then treat as Grade 3 or Grade 4.<br>Study drug/study regimen can be resumed once event stabilizes to Grade ≤1 after completion of steroid taper.<br>Patients with endocrinopathies who may require prolonged or continued steroid replacement can be retreated with study drug/study regimen on the following conditions:<br>1. The event stabilizes and is controlled.<br>2. The patient is clinically stable as per Investigator or treating physician's clinical judgement.<br>3. Doses of prednisone are at ≤10 mg/day or equivalent.<br>**Grade 3** Depending on the individual toxicity, study drug/study regimen may be permanently discontinued. Please refer to guidelines below.<br>**Grade 4** Permanently discontinue study drug/study regimen.<br>Note: For Grade ≥3 asymptomatic amylase or lipase levels, hold study drug/study regimen, and if complete work up shows no evidence of pancreatitis, study drug/study regimen may be continued or resumed.	It is recommended that management of immune-mediated adverse events (imAEs) follows the guidelines presented in this table:<br>– It is possible that events with an inflammatory or immune mediated mechanism could occur in nearly all organs, some of them not noted specifically in these guidelines.<br>– Whether specific immune-mediated events (and/or laboratory indicators of such events) are noted in these guidelines or not, patients should be thoroughly evaluated to rule out any alternative etiology (e.g., disease progression, concomitant medications, and infections) to a possible immune-mediated event. In the absence of a clear alternative etiology, all such events should be managed as if they were immune related. General recommendations follow.<br>– Symptomatic and topical therapy should be considered for low-grade (Grade 1 or 2, unless otherwise specified) events.<br>– For persistent (>3 to 5 days) low-grade (Grade 2) or severe (Grade ≥3) events, promptly start prednisone 1 to 2 mg/kg/day PO or IV equivalent.<br>– Some events with high likelihood for morbidity and/or mortality – e.g., myo-carditis, or other similar events even if they are not currently noted in the guidelines – should progress rapidly to high dose IV corticosteroids (methylprednisolone at 2 to 4 mg/kg/day) even if the event is Grade 2, and if clinical suspicion is high and/or there has been clinical confirmation. Consider, as necessary, discussing with the study physician, and promptly pursue specialist consultation.<br>– If symptoms recur or worsen during corticosteroid tapering (28 days of taper), increase the corticosteroid dose (prednisone dose [e.g., up to 2 to 4 mg/kg/day PO or IV equivalent]) until stabilization or improvement of symptoms, then resume corticosteroid tapering at a slower rate (>28 days of taper).<br>– More potent immunosuppressives such as TNF inhibitors (e.g., infliximab) (also refer to the individual sections of the imAEs for specific type of immunosuppressive) should be considered for events not responding to systemic steroids. Progression to use of more potent immunosuppressives should proceed more rapidly in events with high likelihood for morbidity and/or mortality – e.g., myocarditis, or other similar events even if they are not currently noted in the guidelines – when these events are not responding to systemic steroids.<br>– With long-term steroid and other immunosuppressive use, consider need for *Pneumocystis jirovecii* pneumonia (PJP, formerly known as *Pneumocystis*

Dosing Modification and Toxicity Management Guidelines for Immune-Mediated, Infusion-Related, and Non-Immune–Mediated Reactions (MEDI4736 Monotherapy or Combination Therapy With Tremelimumab or Tremelimumab Monotherapy) 1 November 2017 Version

General Considerations

Dose Modifications	Toxicity Management
Note: Study drug/study regimen should be permanently discontinued in Grade 3 events with high likelihood for morbidity and/or mortality – e.g., myocarditis, or other similar events even if they are not currently noted in the guidelines. Similarly, consider whether study drug/study regimen should be permanently discontinued in Grade 2 events with high likelihood for morbidity and/or mortality – e.g., myocarditis, or other similar events even if they are not currently noted in the guidelines – when they do not rapidly improve to Grade <1 upon treatment with systemic steroids and following full taper<br>Note: There are some exceptions to permanent discontinuation of study drug for Grade 4 events (i.e., hyperthyroidism, hypothyroidism, Type 1 diabetes mellitus).	*carinii* pneumonia) prophylaxis, gastrointestinal protection, and glucose monitoring.<br>– Discontinuation of study drug/study regimen is not mandated for Grade 3/Grade 4 inflammatory reactions attributed to local tumor response (e.g., inflammatory reaction at sites of metastatic disease and lymph nodes). Continuation of study drug/study regimen in this situation should be based upon a benefit-risk analysis for that patient.

AE Adverse event; CTC Common Toxicity Criteria; CTCAE Common Terminology Criteria for Adverse Events; imAE immune-mediated adverse event; IV intravenous; NCI National Cancer Institute; PO By mouth.

Pediatric Considerations

Dose Modifications	Toxicity Management
The criteria for permanent discontinuation of study drug/study regimen based on CTC grade/severity is the same for pediatric patients as it is for adult patients, as well as to permanently discontinue study drug/study regimen if unable to reduce corticosteroid ≤ a dose equivalent to that required for corticosteroid replacement therapy **within 12 weeks** after last dose of study drug/study regimen	– All recommendations for specialist consultation should occur with a pediatric specialist in the specialty recommended.<br>– The recommendations for dosing of steroids (i.e., mg/kg/day) and for IV IG and plasmapheresis that are provided for adult patients should also be used for pediatric patients.<br>– The infliximab 5 mg/kg IV dose recommended for adults is the same as recommended for pediatric patients ≥ 6 years old. For dosing in children younger than 6 years old, consult with a pediatric specialist.<br>– For pediatric dosing of mycophenolate mofetil, consult with a pediatric specialist.<br>– With long-term steroid and other immunosuppressive use, consider need for PJP prophylaxis, gastrointestinal protection, and glucose monitoring.

Specific Immune-Mediated Reactions

Adverse Events	Severity Grade of the Event (NCI CTCAE version 4.03)	Dose Modifications	Toxicity Management
**Pneumonitis/Interstitial Lung Disease (ILD)**	**Any Grade**	**General Guidance**	**For Any Grade:**<br>– Monitor patients for signs and symptoms of pneumonitis or ILD (new onset or worsening shortness of breath or cough). Patients should be evaluated with imaging and pulmonary function tests, including other diagnostic procedures as described below.<br>– Initial work-up may include clinical evaluation, monitoring of oxygenation via pulse oximetry (resting and exertion), laboratory work-up, and high- resolution CT scan.
	**Grade 1**<br>(asymptomatic, clinical or diagnostic observations only; intervention not indicated)	No dose modifications required. However, consider holding study drug/study regimen dose as clinically appropriate and during diagnostic work-up for other etiologies.	**For Grade 1 (radiographic changes only):**<br>– Monitor and closely follow up in 2 to 4 days for clinical symptoms, pulse oximetry (resting and exertion), and laboratory work-up and then as clinically indicated.<br>– Consider Pulmonary and Infectious disease consult.
	**Grade 2**<br>(symptomatic; medical intervention indicated; limiting instrumental ADL)	Hold study drug/study regimen dose until Grade 2 resolution to Grade ≤1.<br>• If toxicity worsens, then treat as Grade 3 or Grade 4.<br>• If toxicity improves to Grade ≤1, then the decision to reinitiate study drug/study regimen will be based upon treating physician's clinical judgment and after completion of steroid taper.	**For Grade 2 (mild to moderate new symptoms):**<br>– Monitor symptoms daily and consider hospitalization.<br>– Promptly start systemic steroids (e.g., prednisone 1 to 2 mg/kg/day PO or IV equivalent).<br>– Reimage as clinically indicated.<br>– If no improvement within 3 to 5 days, additional workup should be considered and prompt treatment with IV methylprednisolone 2 to 4 mg/kg/day started<br>– If still no improvement within 3 to 5 days despite IV methylprednisolone at 2 to 4 mg/kg/day, promptly start immunosuppressive therapy such as TNF inhibitors (e.g., infliximab at 5 mg/kg every 2 weeks). Caution: It is important to rule out sepsis and refer to infliximab label for general guidance before using infliximab.<br>– Once the patient is improving, gradually taper steroids over ≥28 days and consider prophylactic antibiotics, antifungals, or anti-PJP treatment (refer to current NCCN guidelines for treatment of cancer-related infections [Category 2B recommendation])[a]

			– Consider pulmonary and infectious disease consult.<br>– Consider, as necessary, discussing with study physician.
	**Grade 3 or 4**<br>(Grade 3: severe symptoms; limiting self-care ADL; oxygen indicated)<br><br>(Grade 4: life-threatening respiratory compromise; urgent intervention indicated [e.g., tracheostomy or intubation])	Permanently discontinue study drug/study regimen.	**For Grade 3 or 4 (severe or new symptoms, new/worsening hypoxia, life-threatening):**<br>– Promptly initiate empiric IV methylprednisolone 1 to 4 mg/kg/day or equivalent.<br>– Obtain Pulmonary and Infectious disease consult; consider, as necessary, discussing with study physician.<br>– Hospitalize the patient.<br>– Supportive care (e.g., oxygen).<br>– If no improvement within 3 to 5 days, additional workup should be considered and prompt treatment with additional immunosuppressive therapy such as TNF inhibitors (e.g., infliximab at 5 mg/kg every 2 weeks' dose) started. Caution: rule out sepsis and refer to infliximab label for general guidance before using infliximab.<br>– Once the patient is improving, gradually taper steroids over ≥28 days and consider prophylactic antibiotics, antifungals, and, in particular, anti-PJP treatment (refer to current NCCN guidelines for treatment of cancer-related infections [Category 2B recommendation]).[a]
**Diarrhea/Colitis**	**Any Grade**	**General Guidance**	**For Any Grade:**<br>– Monitor for symptoms that may be related to diarrhea/enterocolitis (abdominal pain, cramping, or changes in bowel habits such as increased frequency over baseline or blood in stool) or related to bowel perforation (such as sepsis, peritoneal signs, and ileus).<br>– Patients should be thoroughly evaluated to rule out any alternative etiology (e.g., disease progression, other medications, or infections), including testing for clostridium difficile toxin, etc.<br>– Steroids should be considered in the absence of clear alternative etiology, even for low-grade events, in order to prevent potential progression to higher grade event.<br>– Use analgesics carefully; they can mask symptoms of perforation and peritonitis.
	**Grade 1**<br>(Diarrhea: stool frequency of <4 over baseline per day)	No dose modifications.	**For Grade 1:**<br>– Monitor closely for worsening symptoms.<br>– Consider symptomatic treatment, including hydration, electrolyte replacement, dietary changes (e.g., American

(Colitis: asymptomatic; clinical or diagnostic observations only)		Dietetic Association colitis diet), and loperamide. Use probiotics as per treating physician's clinical judgment.
**Grade 2**<br>(Diarrhea: stool frequency of 4 to 6 over baseline per day)<br>(Colitis: abdominal pain; mucus or blood in stool)	Hold study drug/study regimen until resolution to Grade ≤1<br>• If toxicity worsens, then treat as Grade 3 or Grade 4.<br>• If toxicity improves to Grade ≤1, then study drug/study regimen can be resumed after completion of steroid taper.	**For Grade 2:**<br>– Consider symptomatic treatment, including hydration, electrolyte replacement, dietary changes (e.g., American Dietetic Association colitis diet), and loperamide and/or budesonide.<br>– Promptly start prednisone 1 to 2 mg/kg/day PO or IV equivalent.<br>– If event is not responsive within 3 to 5 days or worsens despite prednisone at 1 to 2 mg/kg/day PO or IV equivalent, GI consult should be obtained for consideration of further workup, such as imaging and/or colonoscopy, to confirm colitis and rule out perforation, and prompt treatment with IV methylprednisolone 2 to 4 mg/kg/day started.<br>– If still no improvement within 3 to 5 days despite 2 to 4 mg/kg IV methylprednisolone, promptly start immunosuppressives such as infliximab at 5 mg/kg once every 2 weeks[a]. **Caution:** it is important to rule out bowel perforation and refer to infliximab label for general guidance before using infliximab.<br>– Consider, as necessary, discussing with study physician if no resolution to Grade ≤1 in 3 to 4 days.<br>– Once the patient is improving, gradually taper steroids over ≥28 days and consider prophylactic antibiotics, antifungals, and anti-PJP treatment (refer to current NCCN guidelines for treatment of cancer-related infections [Category 2B recommendation]).[a]
**Grade 3 or 4**<br>(Grade 3 diarrhea: stool frequency of ≥7 over baseline per day; Grade 4 diarrhea: life threatening consequences)<br>(Grade 3 colitis: severe abdominal pain, change in bowel habits, medi-cal intervention indi-	**Grade 3**<br>Permanently discontinue study drug/study regimen for Grade 3 if toxicity does not improve to Grade ≤1 within 14 days; study drug/study regimen can be resumed after completion of steroid taper.<br>**Grade 4**<br>Permanently discontinue study drug/study regimen.	**For Grade 3 or 4:**<br>– Promptly initiate empiric IV methylprednisolone 2 to 4 mg/kg/day or equivalent.<br>– Monitor stool frequency and volume and maintain hydration.<br>– Urgent GI consult and imaging and/or colonoscopy as appropriate.<br>– If still no improvement within 3 to 5 days of IV methylprednisolone 2 to 4 mg/kg/day or equivalent, promptly start further immunosuppressives (e.g., infliximab at 5 mg/kg once every 2 weeks). **Caution**: Ensure GI consult to rule out bowel perforation and refer to infliximab label for general guidance before using infliximab.<br>– Once the patient is improving, gradually taper steroids over ≥28 days and consider prophylactic antibiotics, antifungals, and

	cated, peritoneal signs; Grade 4 colitis: life-threatening consequences, urgent intervention indicated)		anti-PJP treatment (refer to current NCCN guidelines for treatment of cancer-related infections [Category 2B recommendation]).[a]
**Hepatitis (elevated LFTs)**<br>Infliximab should not be used for management of immune-related hepatitis.<br><br>**PLEASE SEE shaded area immediately below this section to find guidance for management of "Hepatitis (elevated LFTS)" in HCC patients**	**Any Grade**	**General Guidance**	**For Any Grade:**<br>– Monitor and evaluate liver function test: AST, ALT, ALP, and TB.<br>– Evaluate for alternative etiologies (e.g., viral hepatitis, disease progression, concomitant medications).
	**Grade 1**<br>(AST or ALT >ULN and ≤3.0×ULN and/or TB > ULN and ≤1.5×ULN)	• No dose modifications.<br>• If it worsens, then treat as Grade 2 event.	**For Grade 1:**<br>– Continue LFT monitoring per protocol.
	**Grade 2**<br>(AST or ALT >3.0×ULN and ≤5.0×ULN and/or TB >1.5×ULN and ≤3.0×ULN)	• Hold study drug/study regimen dose until Grade 2 resolution to Grade ≤1.<br>• If toxicity worsens, then treat as Grade 3 or Grade 4.<br>• If toxicity improves to Grade ≤1 or baseline, resume study drug/study regimen after completion of steroid taper.	**For Grade 2:**<br>– Regular and frequent checking of LFTs (e.g., every 1 to 2 days) until elevations of these are improving or resolved.<br>– If no resolution to Grade ≤1 in 1 to 2 days, consider, as necessary, discussing with study physician.<br>– If event is persistent (>3 to 5 days) or worsens, promptly start prednisone 1 to 2 mg/kg/day PO or IV equivalent.<br>– If still no improvement within 3 to 5 days despite 1 to 2 mg/kg/day of prednisone PO or IV equivalent, consider additional work up and start prompt treatment with IV methylprednisolone 2 to 4 mg/kg/day.<br>– If still no improvement within 3 to 5 days despite 2 to 4 mg/kg/day of IV methylprednisolone, promptly start immunosuppressives (i.e., mycophenolate mofetil).[a] Discuss with study physician if mycophenolate mofetil is not available. **Infliximab should NOT be used.**<br>– Once the patient is improving, gradually taper steroids over ≥28 days and consider prophylactic antibiotics, antifungals, and anti-PJP treatment (refer to current NCCN guidelines for treatment of cancer-related infections [Category 2B recommendation]).[a]
	**Grade 3 or 4**	**For Grade 3:**	**For Grade 3 or 4:**

	(Grade 3: AST or ALT >5.0×ULN and ≤20.0×ULN and/or TB >3.0×ULN and ≤10.0×ULN)<br><br>(Grade 4: AST or ALT >20×ULN and/or TB >10×ULN)	For elevations in transaminases ≤8 × ULN, or elevations in bilirubin ≤5 × ULN:<br>• Hold study drug/study regimen dose until resolution to Grade ≤1 or baseline<br>• Resume study drug/study regimen if elevations downgrade to Grade ≤1 or baseline within 14 days and after completion of steroid taper.<br>• Permanently discontinue study drug/study regimen if the elevations do not downgrade to Grade ≤1 or baseline within 14 days<br>For elevations in transaminases >8 × ULN or elevations in bilirubin >5 × ULN, discontinue study drug/study regimen.<br>Permanently discontinue study drug/study regimen for any case meeting Hy's law criteria (AST and/or ALT >3 × ULN + bilirubin >2 × ULN without initial findings of cholestasis (i.e., elevated alkaline P04) and in the absence of any alternative cause.[b]<br><br>**For Grade 4:**<br>Permanently discontinue study drug/study regimen.	– Promptly initiate empiric IV methylprednisolone at 1 to 4 mg/kg/day or equivalent.<br>– If still no improvement within 3 to 5 days despite 1 to 4 mg/kg/day methylprednisolone IV or equivalent, promptly start treatment with immunosuppressive therapy (i.e., mycophenolate mofetil). Discuss with study physician if mycophenolate is not available. **Infliximab should NOT be used.**<br>– Perform hepatology consult, abdominal workup, and imaging as appropriate.<br>– Once the patient is improving, gradually taper steroids over ≥28 days and consider prophylactic antibiotics, antifungals, and anti-PJP treatment (refer to current NCCN guidelines for treatment of cancer-related infections [Category 2B recommendation]).[a]
**Hepatitis (elevated LFTs)**	**Any Grade**	**General Guidance**	**For Any Grade:**<br>– Monitor and evaluate liver function test: AST, ALT, ALP, and TB.<br>– Evaluate for alternative etiologies (e.g., viral hepatitis, disease progression, concomitant medications, worsening of liver cirrhosis [e.g., portal vein thrombosis]).<br>– For HBV+ patients: evaluate quantitative HBV viral load, quantitative HBsAg, or HBeAg

Infliximab should not be used for management of immune-related hepatitis.<br><br>**THIS shaded area is guidance *only* for management of "Hepatitis (elevated LFTs)" in HCC patients**<br><br>See instructions at bottom of shaded area if transaminase rise is not isolated but (at any time) occurs in setting of either **increasing bilirubin or signs of DILI/liver decompensation**			– For HCV+ patients: evaluate quantitative HCV viral load<br>– Consider consulting hepatologist/Infectious disease specialist regarding change/implementation in/of antiviral medications for any patient with an elevated HBV viral load >2000 IU/ml<br>– Consider consulting hepatologist/Infectious disease specialist regarding change/implementation in/of antiviral HCV medications if HCV viral load increased by ≥2-fold<br>– For HCV+ with HBcAB+: Evaluate for both HBV and HCV as above
	**Grade 1**<br>(Isolated AST or ALT >ULN and ≤5.0×ULN, whether normal or elevated at baseline)	• No dose modifications.<br>• If ALT/AST elevations represents significant worsening based on investigator assessment, then treat as Grade 2 event.<br>For all grades, see instructions at bottom of shaded area if transaminase rise is not isolated but (at any time) occurs in setting of either **increasing bilirubin or signs of DILI/liver decompensation**	
	**Grade 2**<br>(Isolated AST or ALT >5.0×ULN and ≤8.0×ULN, if normal at baseline)<br><br>(Isolated AST or ALT >2.0×baseline and ≤12.5×ULN, if elevated >ULN at baseline)	• Hold study drug/study regimen dose until Grade 2 resolution to Grade ≤1 or baseline.<br>• If toxicity worsens, then treat as Grade 3 or Grade 4.<br>If toxicity improves to Grade ≤1 or baseline, resume study drug/study regimen after completion of steroid taper.	**For Grade 2:**<br>– Regular and frequent checking of LFTs (e.g., every 1 to 3 days) until elevations of these are improving or resolved.<br>– Recommend consult hepatologist; consider abdominal ultrasound, including Doppler assessment of liver perfusion.<br>– Consider, as necessary, discussing with study physician.<br>– If event is persistent (>3 to 5 days) or worsens, and investigator suspects toxicity to be immune-mediated AE, recommend to start prednisone 1 to 2 mg/kg/day PO or IV equivalent.<br>– If still no improvement within 3 to 5 days despite 1 to 2 mg/kg/day of prednisone PO or IV equivalent, consider additional workup and treatment with IV methylprednisolone 2 to 4 mg/kg/day.<br>– If still no improvement within 3 to 5 days despite 2 to 4 mg/kg/day of IV methylprednisolone, consider additional abdominal workup (including liver biopsy) and imaging (i.e., liver ultrasound), and consider starting immunosuppressives (i.e., mycophenolate mofetil).[a] Discuss with study physician if mycophenolate mofetil is not available. **Infliximab should**

			**NOT be used.**
	**Grade 3**<br>(Isolated AST or ALT >8.0×ULN and ≤20.0×ULN, if normal at baseline)<br><br>(Isolated AST or ALT >12.5×ULN and ≤20.0×ULN, if elevated >ULN at baseline)	• Hold study drug/study regimen dose until resolution to Grade ≤1 or baseline<br>• Resume study drug/study regimen if elevations downgrade to Grade ≤1 or baseline within 14 days and after completion of steroid taper.<br>• Permanently discontinue study drug/study regimen if the elevations do not downgrade to Grade ≤1 or baseline within 14 days<br>Permanently discontinue study drug/study regimen for any case meeting Hy's law criteria, in the absence of any alternative cause.[b]	**For Grade 3:**<br>– Regular and frequent checking of LFTs (e.g., every 1-2 days) until elevations of these are improving or resolved.<br>– Consult hepatologist (unless investigator is hepatologist); obtain abdominal ultrasound, including Doppler assessment of liver perfusion; and consider liver biopsy.<br>– Consider, as necessary, discussing with study physician.<br>– If investigator suspects toxicity to be immune-mediated, promptly initiate empiric IV methylprednisolone at 1 to 4 mg/kg/day or equivalent.<br>– If no improvement within 3 to 5 days despite 1 to 4 mg/kg/day methylprednisolone IV or equivalent, obtain liver biopsy (if it has not been done already) and promptly start treatment with immunosuppressive therapy (mycophenolate mofetil). Discuss with study physician if mycophenolate is not available. **Infliximab should NOT be used.**<br>– Once the patient is improving, gradually taper steroids over ≥28 days and consider prophylactic antibiotics, antifungals, and anti-PCP treatment (refer to current NCCN guidelines for treatment of cancer-related infections [Category 2B recommendation]).[a]
	**Grade 4**<br>(Isolated AST or ALT >20×ULN, whether normal or elevated at baseline)	Permanently discontinue study drug/study regimen.	**For Grade 4:**<br>**Same as above**<br>**(except would recommend obtaining liver biopsy early)**

If transaminase rise is not isolated but (at any time) occurs in setting of either increasing total/direct bilirubin (≥1.5×ULN, if normal at baseline; or 2×baseline, if >ULN at baseline) or signs of DILI/liver decompensation (e.g., fever, elevated INR):

- **Manage dosing for Grade 1 transaminase rise as instructed for Grade 2 transaminase rise**
- **Manage dosing for Grade 2 transaminase rise as instructed for Grade 3 transaminase rise**
- **Grade 3-4: Permanently discontinue study drug/study regimen**

**Nephritis or renal dysfunction**<br>(elevated serum creatinine)	**Any Grade**	**General Guidance**	**For Any Grade:**<br>– Consult with nephrologist.<br>– Monitor for signs and symptoms that may be related to changes in renal function (e.g., routine urinalysis, elevated serum BUN and creatinine, decreased creatinine clearance, electrolyte imbalance, decrease in urine output, or proteinuria).<br>– Patients should be thoroughly evaluated to rule out any alternative etiology (e.g., disease progression or infections).<br>– Steroids should be considered in the absence of clear alternative etiology even for low-grade events (Grade 2), in order to prevent potential progression to higher grade event.
	**Grade 1**<br>(Serum creatinine > 1 to 1.5 × baseline; > ULN to 1.5 × ULN)	No dose modifications.	**For Grade 1:**<br>– Monitor serum creatinine weekly and any accompanying symptoms.<br>• If creatinine returns to baseline, resume its regular monitoring per study protocol.<br>• If creatinine worsens, depending on the severity, treat as Grade 2, 3, or 4.<br>– Consider symptomatic treatment, including hydration, electrolyte replacement, and diuretics.
	**Grade 2**<br>(serum creatinine >1.5 to 3.0 × baseline; >1.5 to 3.0 × ULN)	Hold study drug/study regimen until resolution to Grade ≤1 or baseline.<br>• If toxicity worsens, then treat as Grade 3 or 4.<br>• If toxicity improves to Grade ≤1 or baseline, then resume study drug/study regimen after completion of steroid taper.	**For Grade 2:**<br>– Consider symptomatic treatment, including hydration, electrolyte replacement, and diuretics.<br>– Carefully monitor serum creatinine every 2 to 3 days and as clinically warranted.<br>– Consult nephrologist and consider renal biopsy if clinically indicated.<br>– If event is persistent (>3 to 5 days) or worsens, promptly start prednisone 1 to 2 mg/kg/day PO or IV equivalent.<br>– If event is not responsive within 3 to 5 days or worsens despite prednisone at 1 to 2 mg/kg/day PO or IV equivalent, additional workup should be considered and prompt treatment with IV methylprednisolone at 2 to 4 mg/kg/day started.<br>– Once the patient is improving, gradually taper steroids over ≥28 days and consider prophylactic antibiotics, antifungals, and anti-PJP treatment (refer to current NCCN guidelines for treatment of cancer-related infections [Category 2B recommendation]).[a]

			– When event returns to baseline, resume study drug/study regimen and routine serum creatinine monitoring per study protocol.
	**Grade 3 or 4**<br>(Grade 3: serum creatinine >3.0 × baseline; >3.0 to 6.0 × ULN;<br><br>Grade 4: serum creatinine >6.0 × ULN)	Permanently discontinue study drug/study regimen.	**For Grade 3 or 4:**<br>– Carefully monitor serum creatinine on daily basis.<br>– Consult nephrologist and consider renal biopsy if clinically indicated.<br>– Promptly start prednisone 1 to 2 mg/kg/day PO or IV equivalent.<br>– If event is not responsive within 3 to 5 days or worsens despite prednisone at 1 to 2 mg/kg/day PO or IV equivalent, additional workup should be considered and prompt treatment with IV methylprednisolone 2 to 4 mg/kg/day started.<br>– Once the patient is improving, gradually taper steroids over ≥28 days and consider prophylactic antibiotics, antifungals, and anti-PJP treatment (refer to current NCCN guidelines for treatment of cancer-related infections [Category 2B recommendation]).[a]
**Rash**<br>(excluding bullous skin formations)	**Any Grade**<br>(refer to NCI CTCAE v 4.03 for definition of severity/grade depending on type of skin rash)	**General Guidance**	**For Any Grade:**<br>– Monitor for signs and symptoms of dermatitis (rash and pruritus).<br>– IF THERE IS ANY BULLOUS FORMATION, THE STUDY PHYSICIAN SHOULD BE CONTACTED **AND STUDY DRUG DISCONTINUED.**
	**Grade 1**	No dose modifications.	**For Grade 1:**<br>– Consider symptomatic treatment, including oral antipruritics (e.g., diphenhydramine or hydroxyzine) and topical therapy (e.g., urea cream).
	**Grade 2**	For persistent (>1 to 2 weeks) Grade 2 events, hold scheduled study drug/study regimen until resolution to Grade ≤1 or baseline.<br>• If toxicity worsens, then treat as Grade 3.<br>• If toxicity improves to Grade ≤1 or baseline, then resume drug/study	**For Grade 2:**<br>– Obtain dermatology consult.<br>– Consider symptomatic treatment, including oral antipruritics (e.g., diphenhydramine or hydroxyzine) and topical therapy (e.g., urea cream).<br>– Consider moderate-strength topical steroid.<br>– If no improvement of rash/skin lesions occurs within 3 to 5 days or is worsening despite symptomatic treatment and/or use of moderate strength topical steroid, consider, as necessary,

		regimen after completion of steroid taper.	discussing with study physician and promptly start systemic steroids such as prednisone 1 to 2 mg/kg/day PO or IV equivalent.<br>– Consider skin biopsy if the event is persistent for >1 to 2 weeks or recurs.
	**Grade 3 or 4**	**For Grade 3:**<br>Hold study drug/study regimen until resolution to Grade ≤1 or baseline.<br>If temporarily holding the study drug/study regimen does not provide improvement of the Grade 3 skin rash to Grade ≤1 or baseline within 30 days, then permanently discontinue study drug/study regimen.<br><br>**For Grade 4:**<br>Permanently discontinue study drug/study regimen.	**For Grade 3 or 4:**<br>– Consult dermatology.<br>– Promptly initiate empiric IV methylprednisolone 1 to 4 mg/kg/day or equivalent.<br>– Consider hospitalization.<br>– Monitor extent of rash [Rule of Nines].<br>– Consider skin biopsy (preferably more than 1) as clinically feasible.<br>– Once the patient is improving, gradually taper steroids over ≥28 days and consider prophylactic antibiotics, antifungals, and anti-PJP treatment (refer to current NCCN guidelines for treatment of cancer-related infections [Category 2B recommendation]).[a]<br>– Consider, as necessary, discussing with study physician.
**Endocrinopathy**<br>(e.g., hyperthyroidism, hypothyroidism, Type 1 diabetes mellitus, hypophysitis, hypopituitarism, and adrenal insufficiency; exocrine event of amylase/lipase increased also included in this section)	**Any Grade**<br>(depending on the type of endocrinopathy, refer to NCI CTCAE v4.03 for defining the CTC grade/severity)	**General Guidance**	**For Any Grade:**<br>– Consider consulting an endocrinologist for endocrine events.<br>– Consider, as necessary, discussing with study physician.<br>– Monitor patients for signs and symptoms of endocrinopathies. Non-specific symptoms include headache, fatigue, behavior changes, changed mental status, vertigo, abdominal pain, unusual bowel habits, polydipsia, polyuria, hypotension, and weakness.<br>– Patients should be thoroughly evaluated to rule out any alternative etiology (e.g., disease progression including brain metastases, or infections).<br>– Depending on the suspected endocrinopathy, monitor and evaluate thyroid function tests: TSH, free T3 and free T4 and other relevant endocrine and related labs (e.g., blood glucose and ketone levels, HgA1c).

			– For modest asymptomatic elevations in serum amylase and lipase, corticosteroid treatment is not indicated as long as there are no other signs or symptoms of pancreatic inflammation.<br>– If a patient experiences an AE that is thought to be possibly of autoimmune nature (e.g., thyroiditis, pancreatitis, hypophysitis, or diabetes insipidus), the investigator should send a blood sample for appropriate autoimmune antibody testing.
	**Grade 1**	No dose modifications.	**For Grade 1 (including those with asymptomatic TSH elevation):**<br>– Monitor patient with appropriate endocrine function tests.<br>– For suspected hypophysitis/hypopituitarism, consider consultation of an endocrinologist to guide assessment of early-morning ACTH, cortisol, TSH and free T4; also consider gonadotropins, sex hormones, and prolactin levels, as well as cosyntropin stimulation test (though it may not be useful in diagnosing early secondary adrenal insufficiency).<br>– If TSH < 0.5 × LLN, or TSH >2 × ULN, or consistently out of range in 2 subsequent measurements, include free T4 at subsequent cycles as clinically indicated and consider consultation of an endocrinologist.
	**Grade 2**	For Grade 2 endocrinopathy other than hypothyroidism and Type 1 diabetes mellitus, hold study drug/study regimen dose until patient is clinically stable.<br>• If toxicity worsens, then treat as Grade 3 or Grade 4.<br>Study drug/study regimen can be resumed once event stabilizes and after completion of steroid taper.<br>Patients with endocrinopathies who may require prolonged or continued steroid replacement (e.g., adrenal insufficiency) can be retreated with study drug/study regimen on the following conditions:<br>4. The event stabilizes and is controlled.<br>5. The patient is clinically stable as per investigator or treating physician's clinical judgement.	**For Grade 2 (including those with symptomatic endocrinopathy):**<br>– Consult endocrinologist to guide evaluation of endocrine function and, as indicated by suspected endocrinopathy and as clinically indicated, consider pituitary scan.<br>– For all patients with abnormal endocrine work up, except those with isolated hypothyroidism or Type 1 DM, and as guided by an endocrinologist, consider short-term corticosteroids (e.g., 1 to 2 mg/kg/day methylprednisolone or IV equivalent) and prompt initiation of treatment with relevant hormone replacement (e.g., hydrocortisone, sex hormones).<br>– Isolated hypothyroidism may be treated with replacement therapy, without study drug/study regimen interruption, and without corticosteroids.<br>– Isolated Type 1 diabetes mellitus (DM) may be treated with appropriate diabetic therapy, without study drug/study regimen interruption, and without corticosteroids.<br>– Once patients on steroids are improving, gradually taper immunosuppressive steroids (as appropriate and with guidance of endocrinologist) over ≥28 days and consider prophylactic antibiotics, antifungals, and anti-PJP treatment (refer to current NCCN guidelines for treatment of cancer-related infections [Category 2B recommendation]).[a]

		6. Doses of prednisone are ≤10 mg/day or equivalent.	– For patients with normal endocrine workup (laboratory assessment or MRI scans), repeat laboratory assessments/MRI as clinically indicated.
	**Grade 3 or 4**	For Grade 3 or 4 endocrinopathy other than hypothyroidism and Type 1 diabetes mellitus, hold study drug/study regimen dose until endocrinopathy symptom(s) are controlled.<br>Study drug/study regimen can be resumed once event stabilizes and after completion of steroid taper.<br>Patients with endocrinopathies who may require prolonged or continued steroid replacement (e.g., adrenal insufficiency) can be retreated with study drug/study regimen on the following conditions:<br>4. The event stabilizes and is controlled.<br>5. The patient is clinically stable as per investigator or treating physician's clinical judgement.<br>6. Doses of prednisone are ≤10 mg/day or equivalent.	**For Grade 3 or 4:**<br>– Consult endocrinologist to guide evaluation of endocrine function and, as indicated by suspected endocrinopathy and as clinically indicated, consider pituitary scan. Hospitalization recommended.<br>– For all patients with abnormal endocrine work up, except those with isolated hypothyroidism or Type 1 DM, and as guided by an endocrinologist, promptly initiate empiric IV methylprednisolone 1 to 2 mg/kg/day or equivalent, as well as relevant hormone replacement (e.g., hydrocortisone, sex hormones).<br>– For adrenal crisis, severe dehydration, hypotension, or shock, immediately initiate IV corticosteroids with mineralocorticoid activity.<br>– Isolated hypothyroidism may be treated with replacement therapy, without study drug/study regimen interruption, and without corticosteroids.<br>– Isolated Type 1 diabetes mellitus may be treated with appropriate diabetic therapy, without study drug/study regimen interruption, and without corticosteroids.<br>– Once patients on steroids are improving, gradually taper immunosuppressive steroids (as appropriate and with guidance of endocrinologist) over ≥28 days and consider prophylactic antibiotics, antifungals, and anti-PJP treatment (refer to current NCCN guidelines for treatment of cancer-related infections [Category 2B recommendation]).[a]
**Neurotoxicity**<br>(to include but not be limited to limbic encephalitis and autonomic neuropathy, excluding Myasthenia Gravis and Guillain-Barre)	**Any Grade**<br>(depending on the type of neurotoxicity, refer to NCI CTCAE v4.03 for defining the CTC grade/severity)	**General Guidance**	**For Any Grade:**<br>– Patients should be evaluated to rule out any alternative etiology (e.g., disease progression, infections, metabolic syndromes, or medications).<br>– Monitor patient for general symptoms (headache, nausea, vertigo, behavior change, or weakness).<br>– Consider appropriate diagnostic testing (e.g., electromyogram and nerve conduction investigations).<br>– Perform symptomatic treatment with neurological consult as appropriate.<br>–

	**Grade 1**	No dose modifications.	**For Grade 1:**<br>– See “Any Grade” recommendations above.
	**Grade 2**	For acute motor neuropathies or neurotoxicity, hold study drug/study regimen dose until resolution to Grade ≤1.<br>For sensory neuropathy/neuropathic pain, consider holding study drug/study regimen dose until resolution to Grade ≤1.<br>If toxicity worsens, then treat as Grade 3 or 4.<br>Study drug/study regimen can be resumed once event improves to Grade ≤1 and after completion of steroid taper.	**For Grade 2:**<br>– Consider, as necessary, discussing with the study physician.<br>– Obtain neurology consult.<br>– Sensory neuropathy/neuropathic pain may be managed by appropriate medications (e.g., gabapentin or duloxetine).<br>– Promptly start systemic steroids prednisone 1 to 2 mg/kg/day PO or IV equivalent.<br>– If no improvement within 3 to 5 days despite 1 to 2 mg/kg/day prednisone PO or IV equivalent, consider additional workup and promptly treat with additional immunosuppressive therapy (e.g., IV IG).
	**Grade 3 or 4**	**For Grade 3:**<br>Hold study drug/study regimen dose until resolution to Grade ≤1.<br>Permanently discontinue study drug/study regimen if Grade 3 imAE does not resolve to Grade ≤1 within 30 days.<br>**For Grade 4:**<br>Permanently discontinue study drug/study regimen.	**For Grade 3 or 4:**<br>– Consider, as necessary, discussing with study physician.<br>– Obtain neurology consult.<br>– Consider hospitalization.<br>– Promptly initiate empiric IV methylprednisolone 1 to 2 mg/kg/day or equivalent.<br>– If no improvement within 3 to 5 days despite IV corticosteroids, consider additional workup and promptly treat with additional immunosuppressants (e.g., IV IG).<br>– Once stable, gradually taper steroids over ≥28 days.
**Peripheral neuromotor syndromes**<br>(such as Guillain-Barre and myasthenia gravis)	**Any Grade**	**General Guidance**	**For Any Grade:**<br>– The prompt diagnosis of immune-mediated peripheral neuromotor syndromes is important, since certain patients may unpredictably experience acute decompensations that can result in substantial morbidity or in the worst case, death. Special care should be taken for certain sentinel symptoms that may predict a more severe outcome, such as prominent dysphagia, rapidly progressive weakness, and signs of respiratory insufficiency or autonomic instability.<br>– Patients should be evaluated to rule out any alternative etiology (e.g., disease progression, infections, metabolic syndromes or medications). It should be noted that the diagnosis of immune-

			mediated peripheral neuromotor syndromes can be particularly challenging in patients with underlying cancer, due to the multiple potential confounding effects of cancer (and its treatments) throughout the neuraxis. Given the importance of prompt and accurate diagnosis, it is essential to have a low threshold to obtain a neurological consult.<br>– Neurophysiologic diagnostic testing (e.g., electromyogram and nerve conduction investigations, and “repetitive stimulation” if myasthenia is suspected) are routinely indicated upon suspicion of such conditions and may be best facilitated by means of a neurology consultation.<br>– It is important to consider that the use of steroids as the primary treatment of Guillain-Barre is not typically considered effective. Patients requiring treatment should be started with IV IG and followed by plasmapheresis if not responsive to IV IG.
	**Grade 1**	No dose modifications.	**For Grade 1:**<br>– Consider, as necessary, discussing with the study physician.<br>– Care should be taken to monitor patients for sentinel symptoms of a potential decompensation as described above.<br>– Obtain a neurology consult.
	**Grade 2**	Hold study drug/study regimen dose until resolution to Grade ≤1.<br>Permanently discontinue study drug/study regimen if it does not resolve to Grade ≤1 within 30 days or if there are signs of respiratory insufficiency or autonomic instability.	**For Grade 2:**<br>– Consider, as necessary, discussing with the study physician.<br>– Care should be taken to monitor patients for sentinel symptoms of a potential decompensation as described above.<br>– Obtain a neurology consult<br>– Sensory neuropathy/neuropathic pain may be managed by appropriate medications (e.g., gabapentin or duloxetine).<br>*MYASTHENIA GRAVIS:*<br>○ Steroids may be successfully used to treat myasthenia gravis. It is important to consider that steroid therapy (especially with high doses) may result in transient worsening of myasthenia and should typically be administered in a monitored setting under supervision of a consulting neurologist.<br>○ Patients unable to tolerate steroids may be candidates for treatment with plasmapheresis or IV IG. Such decisions are best made in consultation with a neurologist, taking into account the unique needs of each patient.<br>○ If myasthenia gravis-like neurotoxicity is present, consider starting AChE inhibitor therapy in addition

			to steroids. Such therapy, if successful, can also serve to reinforce the diagnosis.<br>*GUILLAIN-BARRE:*<br>○ It is important to consider here that the use of steroids as the primary treatment of Guillain-Barre is not typically considered effective.<br>○ Patients requiring treatment should be started with IV IG and followed by plasmapheresis if not responsive to IV IG.
	**Grade 3 or 4**	**For Grade 3:**<br>Hold study drug/study regimen dose until resolution to Grade ≤1.<br>Permanently discontinue study drug/study regimen if Grade 3 imAE does not resolve to Grade ≤1 within 30 days or if there are signs of respiratory insufficiency or autonomic instability.<br>**For Grade 4:**<br>Permanently discontinue study drug/study regimen.	**For Grade 3 or 4 (severe or life-threatening events):**<br>– Consider, as necessary, discussing with study physician.<br>– Recommend hospitalization.<br>– Monitor symptoms and obtain neurological consult.<br>*MYASTHENIA GRAVIS:*<br>○ Steroids may be successfully used to treat myasthenia gravis. They should typically be administered in a monitored setting under supervision of a consulting neurologist.<br>○ Patients unable to tolerate steroids may be candidates for treatment with plasmapheresis or IV IG.<br>○ If myasthenia gravis-like neurotoxicity present, consider starting AChE inhibitor therapy in addition to steroids. Such therapy, if successful, can also serve to reinforce the diagnosis.<br>*GUILLAIN-BARRE:*<br>○ It is important to consider here that the use of steroids as the primary treatment of Guillain-Barre is not typically considered effective.<br>○ Patients requiring treatment should be started with IV IG and followed by plasmapheresis if not responsive to IV IG.
**Myocarditis**	**Any Grade**	**General Guidance**<br>Discontinue drug permanently if biopsy-proven immune-mediated myocarditis.	**For Any Grade:**<br>– The prompt diagnosis of immune-mediated myocarditis is important, particularly in patients with baseline cardiopulmonary disease and reduced cardiac function.<br>– Consider, as necessary, discussing with the study physician.<br>– Monitor patients for signs and symptoms of myocarditis (new onset or worsening chest pain, arrhythmia, shortness of breath,

		peripheral edema). As some symptoms can overlap with lung toxicities, simultaneously evaluate for and rule out pulmonary toxicity as well as other causes (e.g., pulmonary embolism, congestive heart failure, malignant pericardial effusion). A Cardiology consultation should be obtained early, with prompt assessment of whether and when to complete a cardiac biopsy, including any other diagnostic procedures.<br>– Initial work-up should include clinical evaluation, BNP, cardiac enzymes, ECG, echocardiogram (ECHO), monitoring of oxygenation via pulse oximetry (resting and exertion), and additional laboratory work-up as indicated. Spiral CT or cardiac MRI can complement ECHO to assess wall motion abnormalities when needed.<br>– Patients should be thoroughly evaluated to rule out any alternative etiology (e.g., disease progression, other medications, or infections)
**Grade 1**<br>(asymptomatic with laboratory (e.g., BNP) or cardiac imaging abnormalities)	No dose modifications required unless clinical suspicion is high, in which case hold study drug/study regimen dose during diagnostic work-up for other etiologies. If study drug/study regimen is held, resume after complete resolution to Grade 0.	**For Grade 1 (no definitive findings):**<br>- Monitor and closely follow up in 2 to 4 days for clinical symptoms, BNP, cardiac enzymes, ECG, ECHO, pulse oximetry (resting and exertion), and laboratory work-up as clinically indicated.<br>- Consider using steroids if clinical suspicion is high.
**Grade 2, 3 or 4**<br>(Grade 2: Symptoms with mild to moderate activity or exertion)<br>(Grade 3: Severe with symptoms at rest or with minimal activity or exertion; intervention indicated)<br>(Grade 4: Life-threatening consequences; urgent	- If Grade 2 -- Hold study drug/study regimen dose until resolution to Grade 0. If toxicity rapidly improves to Grade 0, then the decision to reinitiate study drug/study regimen will be based upon treating physician's clinical judgment and after completion of steroid taper. If toxicity does not rapidly improve, permanently. discontinue study drug/study regimen.<br>If Grade 3-4, permanently discontinue study drug/study regimen.	**For Grade 2-4:**<br>– Monitor symptoms daily, hospitalize.<br>– Promptly start IV methylprednisolone 2 to 4 mg/kg/day or equivalent after Cardiology consultation has determined whether and when to complete diagnostic procedures including a cardiac biopsy.<br>– Supportive care (e.g., oxygen).<br>– If no improvement within 3 to 5 days despite IV methylprednisolone at 2 to 4 mg/kg/day, promptly start immunosuppressive therapy such as TNF inhibitors (e.g., infliximab at 5 mg/kg every 2 weeks). Caution: It is important to rule out sepsis and refer to infliximab label for general guidance before using infliximab.<br>– Once the patient is improving, gradually taper steroids over ≥28 days and consider prophylactic antibiotics, antifungals, or anti-PJP treatment (refer to current NCCN guidelines for treatment of cancer-related infections [Category 2B recommendation]).[a]

	intervention indicated (e.g., continuous IV therapy or mechanical hemodynamic support))		
**Myositis/Polymyositis (“Poly/myositis”)**	**Any Grade**	**General Guidance**	**For Any Grade:**<br>– Monitor patients for signs and symptoms of poly/myositis. Typically, muscle weakness/pain occurs in proximal muscles including upper arms, thighs, shoulders, hips, neck and back, but rarely affects the extremities including hands and fingers; also difficulty breathing and/or trouble swallowing can occur and progress rapidly. Increased general feelings of tiredness and fatigue may occur, and there can be new-onset falling, difficulty getting up from a fall, and trouble climbing stairs, standing up from a seated position, and/or reaching up.<br>– If poly/myositis is suspected, a Neurology consultation should be obtained early, with prompt guidance on diagnostic procedures. Myocarditis may co-occur with poly/myositis; refer to guidance under Myocarditis. Given breathing complications, refer to guidance under Pneumonitis/ILD. Given possibility of an existent (but previously unknown) autoimmune disorder, consider Rheumatology consultation.<br>– Consider, as necessary, discussing with the study physician.<br>– Initial work-up should include clinical evaluation, creatine kinase, aldolase, LDH, BUN/creatinine, erythrocyte sedimentation rate or C-reactive protein level, urine myoglobin, and additional laboratory work-up as indicated, including a number of possible rheumatological/antibody tests (i.e., consider whether a rheumatologist consultation is indicated and could guide need for rheumatoid factor, antinuclear antibody, anti-smooth muscle, antisynthetase [such as anti-Jo-1], and/or signal-recognition particle antibodies). Confirmatory testing may include electromyography, nerve conduction studies, MRI of the muscles, and/or a muscle biopsy. Consider Barium swallow for evaluation of dysphagia or dysphonia.<br>Patients should be thoroughly evaluated to rule out any alternative etiology (e.g., disease progression, other medications, or infections).
	**Grade 1**<br>(mild pain)	- No dose modifications.	**For Grade 1:**<br>– Monitor and closely follow up in 2 to 4 days for clinical symptoms and initiate evaluation as clinically indicated.<br>– Consider Neurology consult.

		– Consider, as necessary, discussing with the study physician.
**Grade 2**<br>(moderate pain associated with weakness; pain limiting instrumental activities of daily living [ADLs])	Hold study drug/study regimen dose until resolution to Grade ≤1.<br>- Permanently discontinue study drug/study regimen if it does not resolve to Grade ≤1 within 30 days or if there are signs of respiratory insufficiency.	**For Grade 2:**<br>– Monitor symptoms daily and consider hospitalization.<br>– Obtain Neurology consult, and initiate evaluation.<br>– Consider, as necessary, discussing with the study physician.<br>– If clinical course is rapidly progressive (particularly if difficulty breathing and/or trouble swallowing), promptly start IV methylprednisolone 2 to 4 mg/kg/day systemic steroids along with receiving input from Neurology consultant<br>– If clinical course is *not* rapidly progressive, start systemic steroids (e.g., prednisone 1 to 2 mg/kg/day PO or IV equivalent); if no improvement within 3 to 5 days, continue additional work up and start treatment with IV methylprednisolone 2 to 4 mg/kg/day<br>– If after start of IV methylprednisolone at 2 to 4 mg/kg/day there is no improvement within 3 to 5 days, consider start of immunosuppressive therapy such as TNF inhibitors (e.g., infliximab at 5 mg/kg every 2 weeks). Caution: It is important to rule out sepsis and refer to infliximab label for general guidance before using infliximab.<br>– Once the patient is improving, gradually taper steroids over ≥28 days and consider prophylactic antibiotics, antifungals, or anti-PJP treatment (refer to current NCCN guidelines for treatment of cancer-related infections [Category 2B recommendation]).[a]
**Grade 3 or 4**<br>(pain associated with severe weakness; limiting self-care ADLs)	**For Grade 3:**<br>Hold study drug/study regimen dose until resolution to Grade ≤1.<br>Permanently discontinue study drug/study regimen if Grade 3 imAE does not resolve to Grade ≤1 within 30 days or if there are signs of respiratory insufficiency.<br>**For Grade 4:**<br>- Permanently discontinue study drug/study regimen.	**For Grade 3 or 4 (severe or life-threatening events):**<br>– Monitor symptoms closely; recommend hospitalization.<br>– Obtain Neurology consult, and complete full evaluation.<br>– Consider, as necessary, discussing with the study physician.<br>– Promptly start IV methylprednisolone 2 to 4 mg/kg/day systemic steroids along with receiving input from Neurology consultant.<br>– If after start of IV methylprednisolone at 2 to 4 mg/kg/day there is no improvement within 3 to 5 days, consider start of immunosuppressive therapy such as TNF inhibitors (e.g., infliximab at 5 mg/kg every 2 weeks). Caution: It is important to rule out sepsis and refer to infliximab label for general guidance before using infliximab.<br>– Consider whether patient may require IV IG, plasmapheresis.<br>– Once the patient is improving, gradually taper steroids over ≥28 days and consider prophylactic antibiotics, antifungals, or anti-PJP treatment (refer to current NCCN guidelines for

treatment of cancer-related infections [Category 2B recommendation]).[a]

[a]ASCO Educational Book 2015 "Managing Immune Checkpoint Blocking Antibody Side Effects" by Michael Postow MD.

[b]FDA Liver Guidance Document 2009 Guidance for Industry: Drug Induced Liver Injury – Premarketing Clinical Evaluation.

AChE Acetylcholine esterase; ADL Activities of daily living; AE Adverse event; ALP Alkaline phosphatase test; ALT Alanine aminotransferase; AST Aspartate aminotransferase; BUN Blood urea nitrogen; CT Computed tomography; CTCAE Common Terminology Criteria for Adverse Events; ILD Interstitial lung disease; imAE immune-mediated adverse event; IG Immunoglobulin; IV Intravenous; GI Gastrointestinal; LFT Liver function tests; LLN Lower limit of normal; MRI Magnetic resonance imaging; NCI National Cancer Institute; NCCN National Comprehensive Cancer Network; PJP *Pneumocystis jirovecii* pneumonia (formerly known as *Pneumocystis carinii* pneumonia); PO By mouth; T3 Triiodothyronine; T4 Thyroxine; TB Total bilirubin; TNF Tumor necrosis factor; TSH Thyroid-stimulating hormone; ULN Upper limit of normal.

Infusion-Related Reactions

Severity Grade of the Event (NCI CTCAE version 4.03)	Dose Modifications	Toxicity Management
**Any Grade**	General Guidance	**For Any Grade:**<br>– Manage per institutional standard at the discretion of investigator.<br>– Monitor patients for signs and symptoms of infusion-related reactions (e.g., fever and/or shaking chills, flushing and/or itching, alterations in heart rate and blood pressure, dyspnea or chest discomfort, or skin rashes) and anaphylaxis (e.g., generalized urticaria, angioedema, wheezing, hypotension, or tachycardia).
**Grade 1 or 2**	**For Grade 1:**<br>The infusion rate of study drug/study regimen may be decreased by 50% or temporarily interrupted until resolution of the event.<br>**For Grade 2:**<br>The infusion rate of study drug/study regimen may be decreased 50% or temporarily interrupted until resolution of the event.<br>Subsequent infusions may be given at 50% of the initial infusion rate.	**For Grade 1 or 2:**<br>– Acetaminophen and/or antihistamines may be administered per institutional standard at the discretion of the investigator.<br>– Consider premedication per institutional standard prior to subsequent doses.<br>– Steroids should not be used for routine premedication of Grade ≤2 infusion reactions.
**Grade 3 or 4**	**For Grade 3 or 4:**<br>Permanently discontinue study drug/study regimen.	**For Grade 3 or 4:**<br>– Manage severe infusion-related reactions per institutional standards (e.g., IM epinephrine, followed by IV diphenhydramine and ranitidine, and IV glucocorticoid).

CTCAE Common Terminology Criteria for Adverse Events; IM intramuscular; IV intravenous; NCI National Cancer Institute.

Non–Immune-Mediated Reactions

Severity Grade of the Event (NCI CTCAE version 4.03)	Dose Modifications	Toxicity Management
**Any Grade**	Note: Dose modifications are not required for AEs not deemed to be related to study treatment (i.e., events due to underlying disease) or for laboratory abnormalities not deemed to be clinically significant.	Treat accordingly, as per institutional standard.
**Grade 1**	No dose modifications.	Treat accordingly, as per institutional standard.
**Grade 2**	Hold study drug/study regimen until resolution to ≤Grade 1 or baseline.	Treat accordingly, as per institutional standard.
**Grade 3**	Hold study drug/study regimen until resolution to ≤Grade 1 or baseline.<br>For AEs that downgrade to ≤Grade 2 within 7 days or resolve to ≤Grade 1 or baseline within 14 days, resume study drug/study regimen administration. Otherwise, discontinue study drug/study regimen.	Treat accordingly, as per institutional standard.
**Grade 4**	Discontinue study drug/study regimen (Note: For Grade 4 labs, decision to discontinue should be based on accompanying clinical signs/symptoms, the Investigator's clinical judgment, and consultation with the Sponsor.).	Treat accordingly, as per institutional standard.

Note: As applicable, for early phase studies, the following sentence may be added: "Any event greater than or equal to Grade 2, please discuss with Study Physician."

AE Adverse event; CTCAE Common Terminology Criteria for Adverse Events; NCI National Cancer Institute.

APPENDIX 9: SUMMARY OF CHARACTERISTICS PRODUCT AND INVESTIVATOR'S BROCHURE

SmPC – FLUOROURACILE EBEWE®

http://agence-prd.ansm.sante.fr/php/ecodex/frames.php?specid=67294648&typedoc=R&ref=R0315740.htm

SmPC– ELVORINE®

http://agence-prd.ansm.sante.fr/php/ecodex/frames.php?specid=62645381&typedoc=R&ref=R0278351.htm

SmPC– IRINOTECAN®

http://agence-prd.ansm.sante.fr/php/ecodex/frames.php?specid=60120812&typedoc=R&ref=R0290828.htm

INFORMATIONS ON DURVALUMAB AND TREMILIMUMAB WILL BE PROVIDED IN THE INVESTIGATOR'S BROCHURE

APPENDIX 10: AUTHORIZED AND PROHIBITED CONCOMITTANT MEDICATIONS

**Supportive medication/class of drug:**	**Usage:**
Concomitant medications or treatments (e.g., acetaminophen or diphenhydramine) deemed necessary to provide adequate prophylactic or supportive care, except for those medications identified as “prohibited,” as listed above	To be administered as prescribed by the Investigator
Best supportive care (including antibiotics, nutritional support, correction of metabolic disorders, optimal symptom control, and pain management [including palliative radiotherapy to non-target lesions, etc])	Should be used, when necessary, for all patients
Inactivated viruses, such as those in the influenza vaccine	Permitted
**Prohibited medication/class of drug:**	**Usage:**
Any investigational anticancer therapy other than those under investigation in this study	Should not be given concomitantly whilst the patient is on study treatment
mAbs against CTLA-4, PD-1, or PD-L1 other than those under investigation in this study	Should not be given concomitantly whilst the patient is on study treatment
Any concurrent chemotherapy, radiotherapy, immunotherapy, or biologic or hormonal therapy for cancer treatment other than those under investigation in this study	Should not be given concomitantly whilst the patient is on study treatment. (Concurrent use of hormones for non-cancer-related conditions [e.g., insulin for diabetes and hormone replacement therapy] is acceptable. Local treatment of isolated lesions, excluding target lesions, for palliative intent is acceptable [e.g., by local surgery or radiotherapy])

Immunosuppressive medications including, but not limited to, systemic corticosteroids at doses exceeding 10 mg/day of prednisone or equivalent, methotrexate, azathioprine, and tumor necrosis factor-α blockers	Should not be given concomitantly, or used for premedication prior to the I-O infusions. The following are allowed exceptions:<br>• Use of immunosuppressive medications for the management of IP-related AEs.<br>• short-term premedication for patients receiving combination agent durvalumuab ± tremelimumab where the prescribing information for the agent requires the use of steroids for documented hypersensitivity reactions.<br>• Use in patients with contrast allergies.<br>• In addition, use of inhaled, topical, and intranasal corticosteroids is permitted.<br><br>A temporary period of steroids will be allowed if clinically indicated and considered to be essential for the management of non-immunotherapy related events experienced by the patient (e.g., chronic obstructive pulmonary disease, radiation, nausea, etc).
Drugs with laxative properties and herbal or natural remedies for constipation	Should be avoided through 90 days after the last dose of tremelimumab during the study.
Sunitinib	Should not be given concomitantly or through 90 days after the last dose of tremelimumab (acute renal failure has been reported with combination therapy of tremelimumab and sunitinib).
EGFR TKIs	Should not be given concomitantly.<br>Should be used with caution in the 90 days post last dose of durvalumab.<br>Increased incidences of pneumonitis (with third generation EGFR TKIs) and increased incidence of transaminase increases (with 1st generation EGFR TKIs) has been reported when durvalumab has been given concomitantly.
Live attenuated vaccines	Should not be given through 30 days after the last dose of IP (including SoC).
Herbal and natural remedies which may have immune-modulating effects	Should not be given concomitantly unless agreed by the sponsor.

APPENDIX 11: SERIOUS ADVERSE EVENT REPORT FORM

**PRODIGE 59 – FFCD 1707 – DURIGAST** T ☐ M ☐<br>**SERIOUS ADVERSE EVENT REPORT FORM (SAE)** CRA Initials βχδ	**Page 1/3**

SPONSOR : FFCD **PRINCIPAL INVESTIGATOR : Pr David TOUGERON**

Study title : A randomized phase II study evaluating FOLFIRI + durvalumab vs FOLFIRI + durvalumab and tremelimumab in second-line treatment of patients with advanced gastric or gastro-oesophageal junction adenocarcinoma. Randomized – non-comparative – multicenter phase II

N° EudraCT : 2018-002014-13

Author of the declaration : Dr ☐ – Pr ☐ - CRA ☐ - Other ☐, specify : ………………………………
Name : .... Center :..
Phone : .... Fax : ....

SAE n° : βδ **Type of report :** ☐ **initial** ☐ **follow-up n° :** βδ
Date of report : βδβδχχχδ

SPACE RESERVED FOR DATA CENTER (CRGA)

Date of reception : βδβδχχχδ **Sponsor reference for the event :**................................

**Patient N°** βχδ	**Patient's initials :** βδ α	**Sex :** ☐ Female ☐ Male
**Date of birth :** βδβδχχχδ		**Inclusion date :** βδβδχχχδ

☐ FOLFIRI + durvalumab
☐ FOLFIRI + durvalumab + tremelimumab (irinotécan 150 mg/m²)
☐ FOLFIRI + durvalumab + tremelimumab (irinotécan 180 mg/m²)

Weight (kg) : ααα **Height (cm) :** ααα

**Serious adverse event :**	**Date of start :** βδβδχχχδ<br>**Date of end :** βδβδχχχδ

**Seriousness criteria**	**Grade/severity**	**Outcome**
☐ hospitalization (or prolongation)<br>☐ medically significant<br>☐ durable or significant disability or incapacity<br>☐ life-threatening<br>☐ death<br>☐ congenital anomaly or fetal malformation	α<br>**Coded as NCI-CTC 4.0**<br>**If not applicable, specify :**<br>1 = mild<br>2 = moderate<br>3 = severe<br>4 = life-threatening<br>5 = death due to SAE	☐ recovered/resolved without sequelae<br>☐ recovered/resolved with sequelae<br>☐ recovering/resolving<br>☐ not recovered/resolved<br>☐ death

If hospitalization Date of admission : BΔBΔBXXΔ ongoing ☐ Date of discharge : BΔBΔBXXΔ

If death Date of death : BΔBΔBXXΔ Death cause ______________________________

Specify : ☐ Death related to SAE ☐ Death for which SAE may have contributed ☐ Death related to SAE

Description
Please describe below the chronological sequence of events including the history of the disease and the relevant concomitant diseases existing in the context of the Serious Adverse Event.

PRODIGE 59 – FFCD 1707 – DURIGAST T ☐ M ☐<br>SERIOUS ADVERSE EVENT REPORT FORM (SAE) CRA Initials βχδ	Page 2/3 SAE n°: βδ<br>initial ☐ follow-up☐<br>Patient N° : βχδ

Drug	Administration	Last dose	Treatment modification due to SAE
**If arm B, specify treatment sequence when SAE occurs :**<br>☐ **FOLFIRI + durvalumab + tremelimumab**<br>☐ **FOLFIRI + durvalumab**			
**Irinotecan**<br>☐ Not applicable	Date of first administration :<br>βδβδχχχδ<br>Date of last administration before SAE :<br>βδβδχχχδ<br>Cycle n°: αα	_________ mg	☐ Dose not changed<br>☐ Dose reduced, specify : new dose : _________ mg<br>☐ Temporary withdrawal, specify date of reintroduction : βδβδχχχδ<br>☐ Definitive withdrawal, specify date : βδβδχχχδ
**Folinic acid**<br>☐ Not applicable	Date of first administration :<br>βδβδχχχδ<br>Date of last administration before SAE :<br>βδβδχχχδ<br>Cycle n°: αα	D-L ☐ L ☐<br>_________ mg	☐ Dose not changed<br>☐ Dose reduced, specify : new dose : _________ mg<br>☐ Temporary withdrawal, specify date of reintroduction : βδβδχχχδ<br>☐ Definitive withdrawal, specify date : βδβδχχχδ
**5-FU bolus**<br>☐ Not applicable	Date of first administration :<br>βδβδχχχδ<br>Date of last administration before SAE :<br>βδβδχχχδ<br>Cycle n°: αα	_________ mg	☐ Dose not changed<br>☐ Dose reduced, specify : new dose : _________ mg<br>☐ Temporary withdrawal, specify date of reintroduction : βδβδχχχδ<br>☐ Definitive withdrawal, specify date : βδβδχχχδ
**5-FU infusion**<br>☐ Not applicable	Date of first administration :<br>βδβδχχχδ<br>Date of last administration before SAE :<br>βδβδχχχδ<br>Cycle n°: αα	_________ mg	☐ Dose not changed<br>☐ Dose reduced, specify : new dose : _________ mg<br>☐ Temporary withdrawal, specify date of reintroduction : βδβδχχχδ<br>☐ Definitive withdrawal, specify date : βδβδχχχδ

**Durvalumab**<br>☐ Not applicable	Date of first administration :<br>βδβδχχχδ<br>Date of last administration before SAE :<br>βδβδχχχδ<br>Cycle n°: αα	_________ mg	☐ Dose not changed<br>☐ Dose reduced, specify : new dose : _________ mg<br>☐ Temporary withdrawal, specify date of reintroduction : βδβδχχχδ<br>☐ Definitive withdrawal, specify date : βδβδχχχδ
**Tremelimumab**<br>☐ Not applicable	Date of first administration :<br>βδβδχχχδ<br>Date of last administration before SAE :<br>βδβδχχχδ<br>Cycle n°: αα	_________ mg	☐ Dose not changed<br>☐ Dose reduced, specify : new dose : _________ mg<br>☐ Temporary withdrawal, specify date of reintroduction : βδβδχχχδ<br>☐ Definitive withdrawal, specify date : βδβδχχχδ

Disparition of event after stop or dose reduced of suspected drugs :
Yes ☐ No ☐ Unknown ☐ Not applicable ☐

Recurrence of event after reintroduction of suspected drugs :
Yes ☐ No ☐ Unknown ☐ Not applicable ☐

Concomitants drugs : (regular treatment of the patient or other drugs received within 15 days)

**Drugs**	**Date of start**	**Ongoing**	**Date of end**	**Dose**	**Indication**
	βδβδχχχδ	☐	βδβδχχχδ		
	βδβδχχχδ	☐	βδβδχχχδ		
	βδβδχχχδ	☐	βδβδχχχδ		
	βδβδχχχδ	☐	βδβδχχχδ		
	βδβδχχχδ	☐	βδβδχχχδ		
	βδβδχχχδ	☐	βδβδχχχδ		
	βδβδχχχδ	☐	βδβδχχχδ		
	βδβδχχχδ	☐	βδβδχχχδ		
	βδβδχχχδ	☐	βδβδχχχδ		
	βδβδχχχδ	☐	βδβδχχχδ		

Causality assesment

**Irinotecan :**	☐ **related**	☐ **not related**	☐ **doubtfully related**	or :	☐ **not applicable**
**Folinic acid :**	☐ **related**	☐ **not related**	☐ **doubtfully related**	or :	☐ **not applicable**
**5-FU bolus :**	☐ **related**	☐ **not related**	☐ **doubtfully related**	or :	☐ **not applicable**
**5-FU infusion :**	☐ **related**	☐ **not related**	☐ **doubtfully related**	or :	☐ **not applicable**

**Durvalumab :**	☐ **related**	☐ **not related**	☐ **doubtfully related**	or :	☐ **not applicable**
**Tremelimumab :**	☐ **related**	☐ **not related**	☐ **doubtfully related**	or :	☐ **not applicable**

If the causality assessment between SAE and study drugs are « not related », which is, to your opinion, the cause of SAE ? (tick the appropriate box(es))

☐ Progression of cancer

☐ Preexisting condition, specify : ___________________________________

☐ Concomitant drug, specify which one : ______________________________

☐ Other illness, specify : ____________________________

☐ Other, specify : ___________________________________

PLEASE ATTACH ANONYMIZED HOSPITALIZATION REPORT, AND, IF NEEDED BIOLOGICAL TESTS, COMPLEMENTARY EXAMS…

Form to fax at Data Center CRGA Dijon Fax : 03 80 38 18 41

DATE : **NAME :** **SIGNATURE :**

APPENDIX 12: RULES FOR PUBLICATION FOR PRODIGE TRIALS

PRODIGE RULES FOR PUBLICATION

The rules for publication that will be used for this study will be those in effect at the time of the last inclusion.

(partnership version of May 3, 2012)

PRODIGE RULES FOR PUBLICATION

Having a good-quality journal publish the PRODIGE trials quickly is a vital objective for disseminating advances in treatment. The PRODIGE coordination committee is responsible for publication, deciding on:

- when the preliminary and definitive results of a trial are published.
  All information arising from trials is confidential, at least until the sponsor, coordinating investigator and statistician have finished the appropriate analysis and verification of the trial.
- the composition of the drafting committee (which generally comprises a maximum of seven members).

The coordination committee may delegate these functions to the trial coordinator.

It validates the choices made and ensures deadlines are met. If the coordination committee does not respond within 1 month of submission by the drafting committee, this may be taken as approval.

1. The drafting committee comprises:

- The committee chair as defined by internal regulation
- The most important contributors

In collaborative, national and international trials, if the other associations have enrolled at least 10% of the sample population, the drafting committee comprises a representative chosen from among the investigators of each of the other associations.

Any coordinator from a country or association who has not enrolled any patients will not be on the drafting committee or be an author of the publication, but will be thanked at the end of the article.

2. The lead author undertakes to submit the article for publication within a time period specified by the coordination committee. This period must be no more than 1 year after the trial has closed. If the lead author cannot do this, the coordination committee may designate a new author who becomes the lead author. To facilitate the writing of articles arising from the trials, a medical writer may be called on and writing workshops may be organized for the lead author in collaboration with the statistician.

3. Before each publication, the study project manager sends the coordination committee the planned list of authors accompanied by a table of inclusions for each investigator center.
The coordination committee validates the number and order of authors before each publication in accordance with the PRODIGE rules for publication. If far from the coordination committee's meetings, validation is made by email. A period of 7 days without reply implies approval.

4. **Title of the publication and oral communications**: the title of the trial must be PRODIGE XX, followed by any name given by the sponsor group.

5. **The publication's authors** are ordered according to work contributed and number of patients enrolled:

- A lead author
- A limited number of investigators, by order of participation. There is generally one investigator per center but the steering committee may decide to name two investigators for some centers. This rule may be weighted so that some small- and medium-sized centers that contribute significantly to enrollment may appear as authors. The coordination committee will approve any such weighting so that no party is wronged.
- If he or she is not the lead author, the trial coordinator, or any person who contributes in a major way to the conception and/or conduct of the trial (such as a co-coordinator), is generally the last author. The coordination committee will decide should there be any disagreement.
- The maximum number of authors permitted by the journals will be used.
- Regardless of the number of patients included, at least one author will represent one of the two partners (FFCD or UNICANCER-GI).
- In spin-off publications and ancillary studies, the authors may be different from those of the original article and reflect the specialty in question – for instance, in trials on radiochemotherapy, an article on radiotherapy may be signed by the co-investigator radiotherapists of the centers that enroll. The first author of the original article is therefore the last author of the spin-off publication.

- The PRODIGE partnership is mentioned in the title or after the authors. If the trial is a collaborative study, the first association mentioned is the one that initiated the trial. The others are mentioned if they included at least 5% of the patients, in order of their contribution.
- For trials sponsored or managed by the FFCD, a member of the INSERM U1231 research unit will be the second last author and will be mentioned as having "equally contributed" if this member is not the lead author so that this work done by the INSERM is taken into consideration.
- For trials sponsored or managed by UNICANCER, a representative of the sponsor will be included in the authors.
- The statistician will be named among the authors, generally after the third place. The statistician may be the first or second author of a spin-off publication.

All contributors who do not appear among the authors are mentioned at the end of the article. Study managers (such as the project manager or data manager) are also mentioned.

One of these may occasionally be mentioned as an author if the PRODIGE coordination committee believes it to be justified.

Partners are thanked, as are the patients and their families.

The authors and sponsor are sent a copy of the manuscript for review before submission to a journal. To have their opinions taken into account, they undertake to reply within 15 working days, or 30 days in the summer.

6. **Oral communications** based on the trial results:

An investigator may, after obtaining the approval of the PRODIGE coordination committee and trial steering committee, present in his or her own name all or part of the trial results in an oral presentation. The authors are generally the same as in the written article, but the order of authorship may vary across articles and communications, and also depending on the conference where the presentation is being made. In certain cases, such as multidisciplinary studies or pathological, biological, endoscopic or imaging studies conducted alongside a therapeutic trial, other authors may be chosen depending on their work. The name of the trial remains PRODIGE XX (see § 3) and the other associations will be mentioned if necessary.

7. **These rules must appear in the appendices of all PRODIGE trial protocols.**

APPENDIX 13: INSURANCE CERTIFICATE

sham
PARTAGEONS PLUS QUE L'ASSURANCE

ATTESTATION D'ASSURANCE

RESPONSABILITÉ CIVILE
PROMOTEUR DE RECHERCHES INTERVENTIONNELLES relevant de l'article L 1121-1, 1° du Code de la santé publique
--
Loi n°2012-300 du 5 mars 2012 et textes d'application subséquent

SOCIÉTÉ HOSPITALIÈRE D'ASSURANCES MUTUELLES
18, rue Edouard Rochet - 69372 LYON CEDEX 08

Atteste que la **FEDERATION FRANCAISE DE CANCEROLOGIE DIGESTIVE**
FACULTE DE MEDECINE
BP 87900
21079 DIJON

A souscrit sous le n° **137681** un contrat d'assurance de la Responsabilité Civile Promoteur d'une Recherche interventionnelle relevant de l'article L 1121-1, 1° (Recherche interventionnelle comportant une intervention sur la personne non justifiée par sa prise en charge habituelle) du Code de la santé publique-conforme aux dispositions de l'article R 1121-4 du même code, afin de couvrir les obligations mises à leur charge en application de l'article L.1121-10 du même Code.

Le contrat couvre la recherche intitulée :

PRODIGE 59 - FFCD 1707 – DURIGAST « Etude de Phase II randomisée évaluant l'efficacité du FOLFIRI + durvalumab vs FOLFIRI + durvalumab + tremelimumab en deuxième ligne de traitement chez des patients présentant un adénocarcinome gastrique avancé ou gastro-oesophagien » (Pr David TOUGERON)

Dates prévisionnelles de début et de fin de la recherche : **Janvier 2019 – Mai 2023**

Nombre prévisionnel de personnes qu'il est prévu d'inclure : **105**

La garantie s'exerce pour les recherches réalisées exclusivement en France métropolitaine et dans les départements et territoires d'Outre-mer.

La présente attestation ne constitue toutefois qu'une présomption d'assurance à la charge de la Société avant validation par les autorités compétentes.

Fait et Certifié, à LYON, 29/11/2018

Quentin GILLY
Souscription et vie des contrats
Direction établissements privés et professionnels de santé

> SHAM - Société Hospitalière d'Assurances Mutuelles
18 rue Édouard Rochet - 69372 LYON Cedex 08
Tél : +33 (0)4 72 75 50 25 - Fax : +33 (0)4 72 74 22 32 - www.sham.fr

Société d'Assurance Mutuelle à cotisations fixes.
Entreprise régie par le code des assurances - 779 860 881 RCS Lyon
N° TVA Intracommunautaire FR 79779860881 - RIP 20041 01007 0033305Z038 15

APPENDIX 14: APPROVAL OF THE IRB

COMPTE RENDU DE DELIBERATION : AVIS FAVORABLE avec observation.

Ont participé à la délibération les membres titulaires et les membres suppléants en cas d'absence du titulaire.

1er collège	2ème collège
Catégorie I - Médecins	*Catégorie IV - Juristes*
Pr M. ANDREJAK (T)	M. T. PERERA (T)
J. DAUCHET (T)(1)	Mme E. GALLET (T)
Dr I. DESAILLY (T)	*Catégorie V - Associations de patients*
Dr G. KRIM (S)(3)	Mme M.P. BERGERET (T)
Dr B. C. GUINHOUYA (S)(1)	Mme M. MINARD (T)
Dr M. PIERSON-MARCHANDISE (S)	
Catégorie II – Médecins généralistes	
Dr P. ELETUFE (T)	
Dr J. DALLE (S)	
Catégorie III - Pharmaciens	
Dr C. VANTYGHEM (T)	
Dr. S. ROUTIER (S)	
(1)Méthodologiste (2) Psychiatre (3) Pédiatre<br>(4) Délégué à la protection des données	(T) = titulaire – (S) = suppléant

Plusieurs remarques majeures émises lors du dernier CPP en date du 15 novembre 2018.

Le but de cette étude repose sur deux bras :

- BRAS A : FOLFIRI+ durvalumab
- BRAS B : FOLFIRI+ durvalumab + trémélimumab

Les différentes remarques formulées par le CPP ont été prises en compte dans cette nouvelle formulation du protocole et des différents documents d'information et de consentement. La liste des centres investigateurs reste limitée aux 44 centres initialement prévus. D'autres centres devraient être ajoutés et feront l'objet de la soumission d'une modification substantielle.

Concernant, l'inscription au fichier national, il convient de préciser si l'inscription est faite car il n'est pas permis au patient de participer simultanément à une autre recherche biomédicale.

L'ANSM a demandé une étude préalable portant sur 11 patients : un rapport sera fait sur cet échantillon avant de pouvoir démarrer l'étude.

AVIS FAVORABLE avec observation sur la nécessité de compléter la phrase sur l'inscription au fichier national.

Le Comité rappelle que le promoteur doit informer le CPP et l'Agence Nationale de Sécurité du Médicament (ANSM) de la **date effective du début de l'essai**, qui correspond à la date de la signature du formulaire de consentement par la première personne qui se prête à la recherche en France.

Si la recherche n'a pas débuté dans un délai de 2 ans suivant l'avis du CPP ou l'autorisation de l'ANSM (article R1123-26 du CSP), l'avis et l'autorisation deviennent caducs. Toutefois, sur justification produite avant l'expiration dudit délai, celui-ci peut être prorogé par le CPP et l'ANSM. De même, le promoteur doit informer le CPP et l'ANSM de la **fin de la recherche** dans les 90 jours maximum ou de son **arrêt anticipé** (15 jours).

Par ailleurs, en application de l'article L. 1123-9 du code de santé publique (CSP), toute **modification substantielle** du dossier initialement soumis doit faire l'objet d'une demande préalable d'avis au CPP.

Les effets/événements indésirables graves ainsi que les faits nouveaux susceptibles de porter atteinte à la sécurité des personnes sont également à déclarer en application de l'article L. 1123-10 du CSP.

Veuillez agréer, Monsieur, l'expression de mes salutations distinguées.

Fait à Amiens, le 16 avril 2019

Pr Michel ANDREJAK
Vice-Président du CPP Nord-Ouest II

APPENDIX 15: ANSM AUTHORIZATION

ansm
Agence nationale de sécurité du médicament et des produits de santé

AUTORISATION D'ESSAI CLINIQUE DE MEDICAMENT A USAGE HUMAIN

Date : 27 NOV. 2018

Identifiants de l'essai clinique			
Titre	PRODIGE 59 - (FFCD 1707) – DURIGAST<br>A randomized phase II study evaluating FOLFIRI + durvalumab vs FOLFIRI + durvalumab and tremelimumab in second-line treatment of patients with advanced gastric or gastro-oesophageal junction adenocarcinoma		
Promoteur	Fédération Francophone de cancérologie digestive		
Réf. à rappeler	MEDAECNAT-2018-09-00059	N° EudraCT	**2018-002014-13**

Expéditeur
ANSM / Direction Produit ONCOH / Equipe Oncologie
Dossier suivi par : Annick NJONGA<br>Mel : aec-essaiscliniques@ansm.sante.fr

Destinataire (demandeur : nom / société / tél.)	
Daniel GONZALEZ<br>Fédération Francophone de cancérologie digestive<br>+33 3 80 39 34 83	
Mél	daniel.gonzalez@u-bourgogne.fr

**CPP destinataire**		**Mél**	
**INCA destinataire**		**mél**	inca-registre-ec@institutcancer.fr

Vu le code de la santé publique et notamment l'article L. 1123-8, et les dispositions réglementaires prises pour son application, et vu le dossier de demande d'autorisation d'essai clinique adressé à l'Agence nationale de sécurité du médicament et des produits de santé (ANSM) ;

Vu les compléments versés par le promoteur en date du 14 et 26 novembre 2018 et notamment le protocole de l'essai cité en objet modifié (version 1.0 datée du 08 novembre 2018), suite à la demande de l'ANSM ;

L'autorisation mentionnée à l'article L. 1123-8 du code de la santé publique est accordée pour l'essai clinique cité en objet.

Direction des médicaments en oncologie, hématologie, transplantation, néphrologie, thérapie cellulaire, produits sanguins et radiopharmaceutiques

Le Directeur
Lotfi BOUDALI

Je vous demande de transmettre toute demande de modifications concernant ce dossier par courriel adressé à la boite : **ams-essaisclinques@ansm.sante.fr** . Lors de l'envoi de ces dossiers, je vous demande de veiller à reporter dans l'objet du message la mention : **MSA/ 2018-002014-13/ MEDAECNAT-2018-09-00059** pour les MS soumises pour autorisation ou pour les dossiers mixtes (comportant des modifications soumises pour autorisation et d'autres pour information).

Confidentialité
Cette transmission est à l'attention exclusive du(des) destinataires ci-dessus mentionné(s) et peut contenir des informations privilégiées et/ou confidentielles. Si vous n'êtes pas le destinataire voulu ou une personne mandatée pour lui remettre cette transmission, vous avez reçu ce document par erreur et toute utilisation, révélation, copie ou communication de son contenu est interdite. Si vous avez reçu cette transmission par erreur, veuillez nous en informer par téléphone immédiatement et nous retourner le message original par courrier. Merci.

Confidentiality
This transmission is intended to the addressee(s) listed above only and may contain preferential or/and confidential information. If you are not the intended recipient, you are hereby notified that you have received the document by mistake and any use, disclosure, copying or communication of the content of this transmission is prohibited. If you have received this transmission by mistake, please call us immediately and return the original message by mail. Thank you.

code : AEC_FOR004 v03

143/147, bd Anatole France - F-93285 Saint-Denis cedex - tél. +33 (0)1 55 87 30 00 - **www.ansm.sante.fr** **Page 1 sur 1**
