# Supplementary material for: FOLFIRI Plus Durvalumab With or Without Tremelimumab in Second-Line Treatment of Advanced Gastric or Gastroesophageal Junction Adenocarcinoma: The PRODIGE 59-FFCD 1707-DURIGAST Randomized Clinical Trial
Source: JAMA Oncol. 2024 Apr 4;10(6):709–17. doi: 10.1001/jamaoncol.2024.0207 (PMC11190792; doi:10.1001/jamaoncol.2024.0207)
Supplement: Supplement 2. — Statistical Analysis Plan [file jamaoncol-e240207-s002.pdf]

A randomized phase II study evaluating FOLFIRI + durvalumab vs FOLFIRI + durvalumab and tremelimumab in second-line treatment of patients with advanced gastric or gastro-oesophageal junction adenocarcinoma

Phase II randomized, non-comparative study

DURIGAST – PRODIGE 59 (FFCD 1707)

Statistical Analysis Plan

Final Analysis

Version 3.0 dated 11/03/2022

Writer : Karine Le Malicot

Review Committee : Pr David Tougeron, Leathicia Ndong, Charles Fuchey, Emilie Barbier

Signature page :

I approve the statistical analysis plan:

**Pr David Tougeron**	Date	Signature
Coordonnator		

**Karine Le Malicot**	Date	Signature
FFCD	14/03/2022	
Biostatistician		

**Emilie Barbier**	Date	Signature
FFCD		
Independent Statistician		

Table des matières

ABBREVIATIONS 5

1 INTRODUCTION 6
1.1 Study objectives 6
1.1.1 Primary objectives 6
1.1.2 Secondary objectives 6
1.1.3 Ancillary analyses 6

2 EXPERIMENTAL PLAN 6
2.1 Study design 6
2.2 Treatment arms 7
2.3 Randomization 7
2.4 Study flow-chart 8
2.5 Sample size justification 8
2.6 Planning / history of study analyses 9

3 STUDY POPULATION 10
3.1 Intent-to-treat population (ITT) 10
3.1 Modified Intent-to-treat population (mITT) 10
3.2 Per protocol population (PP) 10
3.3 Safety population (SP) 10
3.4 Quality of life population (QoL) 10

4 STATISTICAL METHODS OVERVIEW 10
4.1 Softwares 10
4.2 Conventions for dates 10
4.3 Conventions for missing data 11
4.4 Baseline definition 11

5 GENERAL CONSIDERATIONS FOR DATA ANALYSES 11

6 STATISTICAL ANALYSES 13
6.1 Baseline Characteristics 13
6.1.1 Patients eligibility 13
6.1.2 Stratification Criteria 14
6.1.3 Demographics 14
6.1.4 Clinical Characteristics 14
6.1.5 Biological Characteristics 14
6.1.6 Disease Characteristics 15
6.2 Efficacy evaluation 15
6.2.1 Median follow-up time 15

6.2.2 Primary efficacy criterion: Percentage of patients alive without radiological progression at 4 months 15
6.2.3 Secondary efficacy criteria 16
6.3 Safety Evaluation 19
6.3.1 Treatment Administration 19
6.3.2 Toxicities 20
6.3.3 Serious Adverse Event 20
6.4 Other criteria evaluation 20
6.4.1 Subsequent Treatments 20
6.4.2 G-CSF Administration 20
6.4.3 Quality of life (QoL) 21
7 VALIDATION OF ANALYSES BY A THIRD PARTY 22

Abbreviations

BMI : Body Mass Index
BRR : Best response rate
BSA : Body Surface Area
DCR : Disease Control Rate
ITT : Intent-To-Treat
mITT : Modified Intent-to-treat
OS : Overall Survival
PFS : Progression-free survival
PT : Preferred term
QoL : Quality of Life
SOC : System Organ Class
TTP : Time to progression

1 Introduction

1.1 Study objectives

1.1.1 Primary objectives

The primary endpoint is the percentage of patients alive and without radiological progression (according to RECIST 1.1) at 4 months after randomization according to investigator.

1.1.2 Secondary objectives

The secondary objectives are:

- Percentage of patients alive and without progression at 4 months according to centralized review
- Overall survival (OS)
- Time to strategy failure
- Safety profile
- Quality of life (QoL)
- Time to progression (TTP), the progression-free survival (median PFS), the best objective response rate (BRR) and disease control rate (DCR) according to the investigator and centralized review (according RECIST V1.1 and iRECIST criteria)
- Efficacy endpoints (OS, PFS, TTP, BRR and DCR) according to the expression PD-L1 and others biomarkers (see biological study)

1.1.3 Ancillary analyses

Blood and tumor samples will be collected in all patients in order to allow translational research projects (Centre de Ressources Biologiques EPIGENETEC, UMR-S 1147, Paris, France, Headed by Prof. Pierre Laurent-Puig):

- Biomarkers analysis (for more details see "ancillary studies" – Appendix 2): microsatellite instability, immune response/immune score, circulating tumor DNA, tumor mutation load, gastric molecular sub-groups, expression and/or amplification of PD-L1 and PD-L2).
- Stool samples will be collected prospectively in all patients in order to allow analysis of microbiota (16S rRNA to identification of bacteria composing the intestinal microbiota of patients).

2 Experimental plan

2.1 Study design

This study is open and randomized multicenter and non-comparative study,

2.2 Treatment arms

Phase II Study

FOLFIRI plus durvalumab

- Durvalumab: 1500 mg by 1-hour IV infusion.

Every 4 weeks until progression

- FOLFIRI (1 course every 2 weeks, until progression):
  - Irinotecan: 180 mg/m^2 by 2-hour IV infusion,
  - Folinic acid: 400 mg/m^2 (or 200 mg/m^2 if Elvorine) by 2-hours IV infusion,
  - 5-FU bolus: 400 mg/m^2 by 10-minutes IV bolus,
  - Continuous 5-FU: 2400 mg/m^2 by 46-hour IV infusion

FOLFIRI plus durvalumab plus tremelimumab

Induction treatment: 4 cycles (*i.e.* 1 course every 4 weeks)

- Durvalumab: 1500 mg by 1-hour IV infusion - Every 4 weeks.
- Tremelimumab: 75 mg by 1-hour IV infusion - Every 4 weeks (for only 4 cycles).
- FOLFIRI (1 course every 2 weeks, until progression):
  - Irinotecan: 180 mg/m^2 by 2-hour IV infusion
  - Folinic acid: 400 mg/m^2 (or 200 mg/m^2 if Elvorine) by 2-hours IV infusion
  - 5-FU bolus: 400 mg/m^2 by 10-minutes IV bolus
  - Continuous 5-FU: 2400 mg/m^2 by 46-hour IV infusion

Tremelimumab was administered for 4 courses (4 months) and then patient continue to receive FOLFIRI plus durvalumab. In case of progression on FOLFIRI plus durvalumab and disease control, tremelimumab can be re-introduced at investigator discretion.

Patient must have 2 weeks of washout period of first-line treatment before receiving the treatment in the trial. Treatment are repeated every 4 weeks until disease progression, unacceptable toxicity or patient's refusal.

All drugs (irinotecan, folinic acid and 5-FU) except durvalumab and tremelimumab are used in the context of their marketed authorization or recommendations (Thésaurus National de Cancérologie Digestive (www.tncd.org)) in France. Thus, durvalumab and tremelimumab are provided in this clinical trial. A pharmacovigilance follow-up is implemented during the study.

2.3 Randomization

The randomization procedure was performed for the Phase II study.

The randomization was done using minimization technique according to the ratio 1:1 and the following factors are considered for the stratification:

- Center
- Duration of disease control to previous first-line chemotherapy (no disease control vs < 3 months vs ≥ 3 months)

2.4 Study flow-chart

<u>For the phase II study:</u>

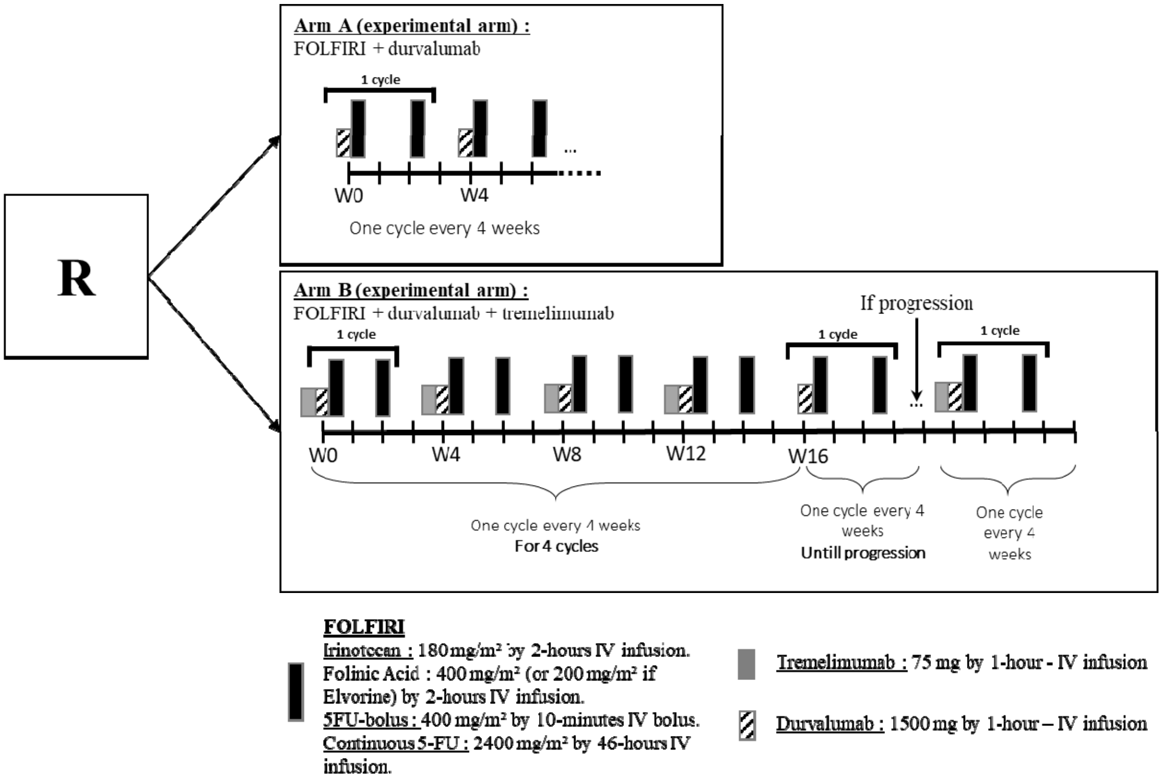

2.5 Sample size justification

Median PFS with FOLFIRI as second-line chemotherapy in gastric and GEJ adenocarcinoma is between 2 to 4 months. We expected at least 5 months median PFS difference with FOLFIRI + durvalumab ± tremelimumab which is clinically significant.

<u>The hypotheses for the randomized phase II were:</u>

H_0: 50% of patients alive and without progression at 4 months is not acceptable.

H_1: 70% of patients alive and without progression at 4 months is expected.

With a risk α (one-sided) of 5%, a power of 85% and according to the binomial exact method (A'Hern), 44 evaluable patients (i.e. patients randomized and with at least one dose of products taken) were needed by arm. Assuming 5% of non-evaluable or lost to follow-up patients, **47 patients were to be included by arm (94 patients in total).**

Taking into account the 11 patients included in the safety run-in phase, 105 patients were be included in the trial.

Rules for selection to be applied to both experimental arms (on the 44 evaluable patients):

- if 28 or more patients are alive without progression at 4 months then the arm will be considered as efficient.

In case both arms will conclude to efficacy, safety data will be analyzed (both Adverse events and Serious Adverse events) in order to see if one has a better safety profile. One or both arms could be compared to a control arm (FOLFIRI) in a phase III study.

2.6 Planning / history of study analyses

For safety run-in phases, patients were treated in 5 expert centers with a huge experience in the use of immune checkpoints inhibitors.

1st step: In order to check the good tolerability of FOLFIRI plus durvalumab combination, 5 patients were treated by FOLFIRI (irinotecan 180mg/m^2) plus durvalumab (1500 mg) in 5 expert centers. The inclusion was stopped at 5 patients. When the 5th patient received 2 cycles of treatment, the safety analysis was done with all the safety data available at this date. The review was done by an Independent Data Monitoring Committee (IDMC) on 07 January 2020.

The decision of IDMC and the data available were sent to ANSM. ANSM approved to re-open the inclusion of patients.

2nd step: 3 patients per arm were randomized to receive either FOLFIRI (irinotecan 180 mg/m^2) plus durvalumab (1500 mg) or FOLFIRI (irinotecan 150 mg/m^2) plus durvalumab (1500 mg) plus tremelimumab (75 mg). These 6 patients were treated in the same 5 expert centers. When the 6th patient received 2 cycles of treatment, the safety analysis was done with all the safety data available at this date (for the 11 patients included in these safety run-in phases). The review was done by an Independent Data Monitoring Committee (IDMC) on the 21 July 2020.
The decision of IDMC and the data available were sent to ANSM. ANSM approved to open the phase II trial.

There was no statistical hypothesis for the safety run-in phase. A total of 11 patients were included in the 2 steps of the safety run-in phase before the randomized phase II study began.

3 Study population

3.1 Intent-to-treat population (ITT)

The intention-to-treat (ITT) population is defined as all patients included in the phase II whatever the eligibility criteria are and the treatment received. Patients will be analyzed according to the allocated group by randomisation, even if they receive a different treatment.

3.1 Modified Intent-to-treat population (mITT)

The modified intention-to-treat (mITT) population is defined as all patients whatever the eligibility criteria and who received at least one dose of treatment in the study. Patients will be analyzed according to randomized treatment.

3.2 Per protocol population (PP)

The per protocol population (PP) is defined as all patients with no major deviation on the eligibility criteria, who received at least two doses of treatment in the study and a survival superior to 3 months. Patients will be analyzed according to randomized treatment.

3.3 Safety population (SP)

The safety population (SP) is defined as all patients who have had received at least one dose of treatment in the study. Patients will be analyzed according to treatment received.

3.4 Quality of life population (QoL)

The Quality of life (QoL) population is defined as all mITT patients with a baseline questionnaire and at least one questionnaire during follow-up. Patients will be analyzed according to the received arm.

4 Statistical methods overview

4.1 Softwares

Statistical analyses will be done with SAS (Statistical Analysis System, SAS Institute, North Carolina, USA). version 9.4.

4.2 Conventions for dates

Randomization/Inclusion date will be considered as Day 1. The previous day is defined as Study day –1 (no Study day 0 is defined).

Duration will be calculated according the following rule:

As an example, time between death and randomization: Date of death - Date of randomization + 1. Date of last news will the later date between date of clinical exam, date of last treatment administered or date of last contact.

Rules for conversion in month or later will be the usual ones:

- 1 month = 30.4375 days
- 1 year = 365.25 days

4.3 Conventions for missing data

Except for specific cases, missing data will not be replaced.

The following conventions will be used for completing dates:

For the start dates:

- if the day is missing (UK/01/2012), the day "01" will be used (01/01/2012)
- if the month is missing (UK/UK/2012), the month "01" will be used (01/01/2012).

For the end dates:

- if the day is missing (UK/01/2012), the day "30" will be used (30/01/2012 – warning: be careful for February)
- if the month is missing (UK/UK/2012), the month "12" will be used (30/12/2012).

For other dates:

- if the day is missing (UK/01/2012), the 15 of the month will be used (15/01/2012)
- if the month is missing (UK/UK/2012), the month "06" will be used (15/06/2012).

4.4 Baseline definition

Baseline measures will be the last measure done before the inclusion/randomization. In case of missing data, the last measure could also be the last measure before the first treatment intake.

5 General considerations for data analyses

The ***quantitative*** variables will be described by the usual statistics: n, mean, standard deviation, median, interquartile range, minimum and maximum. They can also be categorized according to cut-offs of the medical literature.

The ***qualitative*** variables will be described using number and percentages. The missing values will not be counted for the percentage calculation.

The ***time to event endpoint*** will be estimated and plotted using the Kaplan-Meier estimator (Kaplan and Meier, 1958). Number of events will be described according treatment arms. Survival curves and also % at different time-points (and their 95%CI) will be also estimated. The median time and the rates at different times will be described with their 95% confidence interval. The standard error will be estimated by the Greenwood formula and the log-log transformation will be used to compute confidence intervals.

Median follow-up time will be calculated using the reverse Kaplan-Meier method (Schemper et Smith, 1996)[1].

Excepted particular cases, for example baseline characteristics, results will be described by treatment arms.

[1] Schemper, M., & Smith, T. L. (1996). A note on quantifying follow-up in studies of failure time. Controlled clinical trials, 17(4), 343-346.

6 Statistical Analyses

	ITT	mITT	SP	PP	QoL
Eligibility	X				
Baseline characteristics		X		X	
**Primary criterion**					
Percentage of patients alive without progression (RECIST V1.1) at 4 months		X		X	
**Secondary criteria**					
Time to progression (RECIST V1.1)		X		X	
Progression-free survival (median PFS) RECIST V1.1		X		X	
Objective Response rate (RECIST V1.1)		X		X	
Disease control rate (RECIST V1.1)		X		X	
Time to progression (according iRECIST criteria)					
Progression-free survival (median PFS) according to iRECIST V1.1					
Objective Response rate (iRECIST criteria)					
Disease control rate (iRECIST V1.1)					
Time to strategy failure		X			
Overall survival		X		X	
Toxicities			X		
Treatment intake			X		
Quality of life QLQ-C30					X
**Central review**					
Time to progression RECIST V1.1		X			
Time to progression iRECIST		X			
Progression-free survival RECIST V1.1		X			
Progression-free survival iRECIST		X			
Best objective response rate RECIST V1.1		X			
Best objective response rate iRECIST		X			
Disease control rate RECIST V1.1		X			
Disease control rate iRECIST		X			
**Ancillary analyses**					
Efficacy endpoints (OS, PFS, TTP, BRR and DCR) according to the expression PD-L1 and others biomarkers		X			

6.1 Baseline Characteristics

Baseline characteristics (except eligibility done on ITT population) will be described by treatment arms and on the overall population in mITT and PP population.

6.1.1 Patients eligibility

- the number of patients who all inclusion criteria are respected,

- the number of patients who all non-inclusion criteria are respected,
- the number of patients who all eligibility criteria (i.e inclusion and non-inclusion) are respected,
- patient data listing of protocol deviations,
- the number of patients per population will be described (reason for non-inclusion will be described)

6.1.2 Stratification Criteria

Stratification factors will be described (from the randomization form) according to treatment arms to ensure the correct balancing of factors (on ITT population). No statistical tests will be done.

Durée de contrôle de la maladie avec la chimiothérapie de 1ère ligne (pas de contrôle vs contrôle de la maladie < 3 mois vs contrôle de la maladie ≥ 3 mois).

6.1.3 Demographics

- Center
- Age (year)
- Gender (Male *vs* Female)

6.1.4 Clinical Characteristics

- ECOG at baseline
- BMI (Kg/m^2)

6.1.5 Biological Characteristics

- Haemoglobin (g/dL)
- Platelets ($10^3/mm^3$)
- PNN ($/mm^3$)
- Leucocytes ($/mm^3$)
- Lymphocytes ($/mm^3$)
- Total bilirubin (µmol/L)
- Creatinine clairance (mL/Min)
- ASAT (UI/L)
- ALAT (UI/L)
- GGT (UI/L)
- PAL (UI/L)
- LDH (UI/L)
- Albumin (g/L)
- Pre-Albumin (g/L)
- CRP (mg/L)
- Uracilémie

6.1.6 Disease Characteristics

- Delay between primary tumor diagnosis and inclusion (months)
- HER2 status
- Type of tumor
- MSI status
- Method used to determine MSI status
- Location of the primary tumor
- Primary tumor resection (Delay between resection and inclusion, margin quality)
- Type of disease
- Localisation of metastases (if applicable), resection, localisation of resected metastases and margin quality
- Previous Treatment :
    - Neo-adjuvant treatment with schema description
    - Adjuvant treatment with schema description
    - 1st line treatment with schema description and reason of stop

6.2 Efficacy evaluation

Efficacy analyses will be done on the mITT and PP population. Statistics will be presented by treatment arms.

6.2.1 Median follow-up time

Median follow-up is defined as the time between date of randomization and the last news date (Alive or lost-to-follow-up patients) or death (whatever the cause is). The median follow-up time and its 95%CI will be calculated in months by reverse Kaplan-Meier method. It will be described by treatment arms and on the whole population.

6.2.2 Primary efficacy criterion: Percentage of patients alive without radiological progression at 4 months

6.2.2.1 Definition

The primary endpoint is the percentage of patients alive and without radiological progression (according to RECIST 1.1) at 4 months after randomization according to investigator.
Progression will be assessed by the investigator according to RECIST 1.1 criteria on the basis of imagery performed every 8 weeks, Imagery done until <4.5 months will be considered for the evaluation of the progression.

Patients with progression on imageries prior to 4.5 months will be considered as progressing at 4 months.

Patients without progression on imageries prior to 4 months will be reviewed in case of a scan between 4 and 4.5 to determine if progressive at 4 months.

A medical review will be performed to decide on the case of patient lost to follow-up or not evaluable at 4 months without progression identified before 4 months; based on the patient's complete file, the patient may be considered as a treatment failure (progression), or as really not evaluable and not participating in the analysis of the primary endpoint.

6.2.2.2 Evaluation

The rules for selection to be applied to both experimental arms (on the 44 evaluable patients):

- if 28 or more patients are alive without progression at 4 months then the arm will be considered as efficient.

The percentage of patients alive and without progression at 4 months will be described with its two-sided 90% confidence interval.

6.2.3 Secondary efficacy criteria

6.2.3.1 Progression free survival (PFS)

6.2.3.1.1 Definition

Progression free survival (PFS) is defined as the time between date of randomization and date of the first radiological progression (according to RECIST 1.1) or death (from any cause), whichever occurs first. Patients alive without progression will be censored at date of last news.

6.2.3.1 Evaluation

The time scale considered is the month.

Progression-free survival will be plotted using the Kaplan Meier estimator and the rates will be given at different time points along with their 95% confidence intervals as well as the median.

6.2.3.2 Overall Survival (OS)

6.2.3.2.1 Definition

Overall Survival (OS) is defined as the time between date of randomization and date of death (from any cause). Patients alive will be censored at date of last news.

6.2.3.2.2 Evaluation

The time scale considered is the month.

Overall survival will be plotted using the Kaplan Meier estimator and the rates will be given at different time points along with their 95% confidence intervals as well as the median.

6.2.3.3 Time to progression (TTP)

6.2.3.3.1 Definition

Time to progression (TTP) is defined as the time between date of randomization and the date of first radiological progression (according to RECIST v1.1) or death linked to cancer. Patients without progression will be censored at date of last news or date of death. The death not linked to cancer will not be considered as an event.

6.2.3.3.2 Evaluation

The time scale considered is the month.
Time to progression will be plotted using the Kaplan Meier estimator and the rates will be given at different time points along with their 95% confidence intervals as well as the median.

6.2.3.4 Best Objective Response rate (BRR):

6.2.3.4.1 Definition

Best Objective Response rate (BRR) is defined as complete or partial response at the best response evaluation during the treatment according to RECIST v1.1.
Imageries collected during the protocol treatment period (one month after last cycle or definitive end of treatment) and before the beginning of a subsequent treatments will be considered.
Waterfall plots will be done to measure the quality of the best response.
For complete response and partial response the duration of the response will be calculated taking into account the next progression or death after the objective response

6.2.3.5 Evaluation

Percentages will be described with the usual statistics by treatment arms.

6.2.3.6 Disease control rate (DCR)

6.2.3.6.1 Definition

Disease control rate (DCR) is defined as complete or partial response or stable disease at the best response evaluation according to RECIST v1.1.

6.2.3.6.2 Evaluation

Percentages will be described with the usual statistics by treatment arms.

6.2.3.7 Time to strategy failure

6.2.3.7.1 Definition

Time to strategy failure is defined as the time between randomization date and date of death (from any cause) or the date of first radiological progression (RECIST V1.1) in the FOLFIRI + durvalumab arm or date of the second radiological progression after re-introduction of tremelimumab in the FOLFIRI/durvalumab/tremelimumab arm or date of definitive discontinuation.

In case a treatment is stopped for toxicity reason but re-introduced later for progression, then this progression will not be considered for this endpoint.

6.2.3.7.2 Evaluation

Time to strategy failure will be described with the usual statistics by treatment arms.

6.2.3.8 Central review

6.2.3.8.1 Progression-free Survival (PFS)

For RECIST v1.1 evaluation, same definition as §6.2.3.1.

A secondary analysis will be done in iRECIST for both arms. The progression to be taken into account is the first Progression confirmed (iCPD).

6.2.3.8.2 Time to progression (TTP)

For RECIST v1.1 evaluation, same definition as § 6.2.3.3.

Secondary analysis will be done in iRECIST for both arms. The progression to be taken into account is the first Progression confirmed (iCPD).

6.2.3.8.3 Best objective response (BRR)

For RECIST v1.1 evaluation, same definition as § 6.2.3.4

Secondary analysis will be done in iRECIST for both arms.

6.2.3.8.4 Disease control duration

For RECIST v1.1 evaluation, same definition as § 6.2.3.5

Secondary analysis will be done in iRECIST for both arms.

6.2.3.8.5 Ancillary analyses

Centralized radiological assessments of RECIST v1.1 response and iRECIST response according Seymour *et al.* criteria. For exploration, secondary endpoints (OS, PFS, TTP, BRR and DCR) will be analysed according to this centralized review.

6.3 Safety Evaluation

All safety analyses will be done on SP population and presented by treatment arms.

6.3.1 Treatment Administration

6.3.1.1 Treatment duration

Treatment duration will be calculated as follow:

Last treatment administration (D15 or D1) - D1 of the first treatment administration + 1

This duration will be evaluated in month. Free-chemotherapy intervals and number of days for cycles delayed will not be subtracted of this time.

In case of free-chemotherapy interval, if the protocol is not re-introduced then the date of end of treatment could be the date of a subsequent line.

It will be described using usual descriptive statistics by treatment arms.

6.3.1.2 Doses administered

The following will be described by treatment arm:

- the number of patients with at least one dose of treatment,
- the number of cures performed overall and by products

It will be interesting to see if patients stopped Irinotecan and continue under 5FU+immunotherapy or only on immunotherapy.

In case of the body surface area (BSA) is not indicated in the CRF it will be calculated using the following formula (Gehan and Georges):

$$0.0235 \times \text{height(cm)}^{0.42246} \times \text{weight(kg)}^{0.51456}$$

The weight is the weight x indicated by the investigator to the cure x

If weight is missing, the previous weight indicated will be used.

The dose received and the percentages of actual dose received over theoretical dose will be described by patient and summarized by treatment.

Theoretical doses depend of the treatment taken:

- 5FU bolus : 400 mg/m^2
- 5FU continuous : 2400 mg/m^2
- Irinotecan : 180 mg/m^2
- Durvalumab : 1500 mg
- Tremelimumab: 75 mg

6.3.1.3 Dose modifications and administration postponement

The following will be summarized by treatment arm:

- Number and percentage of patients presenting at least one dose modification

- Number and percentage of patients presenting at least one administration postponement
- Reasons for modifications/postponement will be tabulated

6.3.1.4 Definitive treatment stop

The number and the percentage of patients with a definitive stop of treatment as well as the reason of definitive stop (% over the number of patients with a definitive stop) will be described by treatment arm.

6.3.2 Toxicities

Toxicities (graded according NCI-CTC v 4.0) will be described by treatment arms and regarding the treatment causality (Linked/doubtful versus not linked) with:

- Number and percentage of patients presenting at least one maximal grade 3-4 toxicities and those presenting at least one maximal grade 1-2, over the whole treatment period.
- Number and percentage of patients presenting at least one maximal grade 3-4 toxicities and those presenting at least one maximal grade 1-2, over the whole treatment period, by types of toxicities (SOC: System Organ Class) and preferred term (PT).

A listing of grade 5 toxicities will be also provided.

A listing will done to describe the persisting toxicities.

6.3.3 Serious Adverse Event

Summary of SAEs will be provided by the PV department.

6.4 Other criteria evaluation

6.4.1 Subsequent Treatments

Analysis will be done on SP population.

Subsequent treatments will be described by treatment arms according the line of treatments (if more than one).

6.4.2 G-CSF Administration

Analysis will be done on SP population.

The number of patients who took G-CSF will be described as well as the type of prophylaxis and the type of treatment by treatment arms.

6.4.3 Quality of life (QoL)

Quality of life (QoL) will be evaluated using EORTC QLQ-C30 and the STO22 questionnaires and will be done on the QOL population.

6.4.3.1 QLQ-C30 questionnaire

The QLQ-C30 is a cancer-specific tool composed of 30 items. Five functional scores (physical, role, cognitive, social, and emotional), a global health score ranging from 0 (worst) to 100 (best) have been developed as well as 9 symptom scores (nausea, pain, fatigue, dyspnoea, difficulty sleeping, anorexia, constipation, diarrhea and perceived financial difficulties) ranging from 0(best) to 100 (worse).
Scores will be calculated in agreement with the scoring EORTC manual.
The scores of EORTC QLQ-C30 will be described at baseline (last questionnaire before the start of treatment ie date of questionnaire ≤ date of first treatment administration), by treatment arm on the QoL population.

Number and percentage of patients with at least one questionnaire at baseline and during the study will be described by treatment arms.

6.4.3.1.1 Survival without QoL deterioration

6.4.3.1.1.1 Definition

Survival without QoL deterioration is defined as the time interval between randomization and the occurrence of a definitive deterioration ≥ 5 points or death based on the global health score.
A definitive deterioration ≥ 5 points is defined as a decrease in QLQ-C30 QL2 score ≥ 5 points (compared to the QoL score at inclusion) without any further improvement in QoL score ≥ 5 points or any further available QoL data.
Patients alive without definitive deterioration will be censored at the last follow-up.

6.4.3.1.1.2 Evaluation

Months will be considered as time scale.
Survival without QoL deterioration will be analyzed using the Kaplan Meier method on QoL population. The description will be made by treatment arm using the median, and the rates will be estimated at different stages of evaluation (95% confidence intervals will also be provided).

6.4.3.1 STO-22 questionnaire

The STO-22 questionnaire is composed of 22 items. Nine scores are calculated: one functional ability score (body image) and eight symptom scores (dysphagia, pain, reflux symptoms, food restrictions, anxiety, dry mouth, taste, hair loss).

Number and percentage of patients with at least one questionnaire will be described for baseline, on study and both baseline and on-study.

Scores will be described by treatment for patients having both a baseline and at least one questionnaire on-study with:

- Baseline evaluation of the score
- Last evaluation on treatment of the score
- Difference between both score

Evaluation across time for each score could also be done in case of sufficient number of questionnaires.

7 Validation of analyses by a third party

The primary endpoint will be analyzed by another statistician (PFS evaluated by investigators according to RECIST 1.1 in mITT population) to consolidate the conclusion of the study.
