# Supplementary material for: FOLFIRI Plus Durvalumab With or Without Tremelimumab in Second-Line Treatment of Advanced Gastric or Gastroesophageal Junction Adenocarcinoma: The PRODIGE 59-FFCD 1707-DURIGAST Randomized Clinical Trial
Source: JAMA Oncol. 2024 Apr 4;10(6):709–17. doi: 10.1001/jamaoncol.2024.0207 (PMC11190792; doi:10.1001/jamaoncol.2024.0207)
Supplement: Supplement 3. — eTable 1. Univariate and multivariate analysis of predictive of factors of progression-free survival eFigure 1. Progression-free survival according to combined positive score (CPS) eFigure 2. Progression-free survival according to tumor proportion score (TPS) eFigure 3. Time to deterioration (10 points) in global quality of life score [file jamaoncol-e240207-s003.pdf]

Tougeron D, Dahan L, Evesque L, et al. FOLFIRI Plus Durvalumab With or Without Tremelimumab in Second-Line Treatment of Advanced Gastric or Gastroesophageal Junction Adenocarcinoma: The PRODIGE 59-FFCD 1707-DURIGAST Randomized Clinical Trial. JAMA Oncol. Published online April 4, 2024. doi:10.1001/jamaoncol.2024.0207

***First Name and Middle Initial(s)**	***Last Name**	***Suffix (eg, Jr, III)**	Academic Degrees	Institution	Location (city, state/province, country)	Role or Contribution, eg, chair, principal investigator	Group (if more than 1 Group listed in the byline) and/or Subgroup (eg, Steering Committee)
Vincent	HAUTEFEUILLE		DR	CHU HÔPITAL SUD	AMIENS (FRANCE)	Investigator	
Anne	THIROT-BIDAULT		DR	HOPITAL PRIVÉ HOPITAL PRIVE	ANTONY (FRANCE)	Investigator	
Rania	BOUSTANY-GRENIER		DR	CLINIQUE PRIVÉE INSTITUT DU CANCER AVIGNON PROVENCE	AVIGNON (FRANCE)	Investigator	
Marjorie	FAURE		DR	CLINIQUE PRIVÉE BELHARRA	BAYONNE (FRANCE)	Investigator	
Christophe	DEBELLEIX		DR	CLINIQUE PRIVÉE TIVOLI	BORDEAUX (FRANCE)	Investigator	
David	TAVAN		DR	CLINIQUE PRIVÉE INFIRMERIE PROTESTANTE	CALUIRE ET CUIRE (FRANCE)	Investigator	
Amr	EL WESHI		DR	CH CHP DU COTENTIN	CHERBOURG-EN-COTENTIN (FRANCE)	Investigator	
You-Heng	LAM		DR	CH	CHOLET (FRANCE)	Investigator	
Denis	PEZET		PR	CHU ESTAING	CLERMONT FERRAND (FRANCE)	Investigator	
Marion	BOLLIET		DR	CH HOPITAUX CIVILS DE COLMAR	COLMAR (FRANCE)	Investigator	
Ariane	DARUT-JOUVE		DR	CLINIQUE PRIVÉE INSTITUT DE CANCÉROLOGIE DE BOURGOGNE GRRECC	DIJON (FRANCE)	Investigator	
Jean-François	PAITEL		DR	CH FREJUS ST RAPHAEL	FREJUS (FRANCE)	Investigator	
Aurélien	CARNOT		DR	CAC OSCAR LAMBRET	LILLE (FRANCE)	Investigator	
Diane	PANNIER		DR	CAC OSCAR LAMBRET	LILLE (FRANCE)	Investigator	
Valérie	LE BRUN LY		DR	CHU DUPUYTREN	LIMOGES (FRANCE)	Investigator	
Jérôme	DESRAME		PR	CLINIQUE PRIVÉE JEAN MERMOZ	LYON (FRANCE)	Investigator	
Julie	GIGOUT		DR	HOPITAL PRIVÉ HOPITAL EUROPEEN MARSEILLE	MARSEILLE (FRANCE)	Investigator	
Philippe	DOMINICI		DR	HOPITAL PRIVÉ HOPITAL EUROPEEN MARSEILLE	MARSEILLE (FRANCE)	Investigator	

*First Name and Middle Initial(s)	*Last Name	*Suffix (eg, Jr, III)	Academic Degrees	Institution	Location (city, state/province, country)	Role or Contribution, eg, chair, principal investigator	Group (if more than 1 Group listed in the byline) and/or Subgroup (eg, Steering Committee)
Muriel	DULUC		DR	CHU AP-HM LA TIMONE	MARSEILLE (FRANCE)	Investigator	
Emmanuelle	NORGUET MONNEREAU		DR	CHU AP-HM LA TIMONE	MARSEILLE (FRANCE)	Investigator	
Christophe	LOCHER		DR	CH GHI DE L'EST FRANCILIEN SITE DE MEAUX	MEAUX (FRANCE)	Investigator	
Morgan	ANDRE		DR	CH LAYNÉ	MONT DE MARSAN (FRANCE)	Investigator	
Eric	FRANCOIS		DR	CAC ANTOINE LACASSAGNE	NICE (FRANCE)	Investigator	
Romain	COHEN		DR	CHU AP-HP SAINT ANTOINE	PARIS (FRANCE)	Investigator	
Olivier	DUBREUIL		DR	HOPITAL PRIVÉ GROUPE HOSPITALIER DIACONESSES CROIX SAINT SIMON	PARIS (FRANCE)	Investigator	
Mostefa	BENNAMOUN		DR	HOPITAL PRIVÉ MONTSOURIS	PARIS (FRANCE)	Investigator	
Christophe	LOUVET		PR	HOPITAL PRIVÉ MONTSOURIS	PARIS (FRANCE)	Investigator	
Emilie	SOULARUE		DR	HOPITAL PRIVÉ MONTSOURIS	PARIS (FRANCE)	Investigator	
Eric	TERREBONNE		DR	CHU HAUT LÉVÊQUE	PESSAC (FRANCE)	Investigator	
Rayan	ELFADEL		DR	CHU LA MILETRIE	POITIERS (FRANCE)	Investigator	
Aurélie	FERRU		DR	CHU LA MILETRIE	POITIERS (FRANCE)	Investigator	
Damien	BOTSEN		DR	CHU ROBERT DEBRÉ	REIMS (FRANCE)	Investigator	
Romain	DESGRIPPES		DR	CH	SAINT MALO (FRANCE)	Investigator	
Thierry	MURON		DR	CHU INSTITUT CANCEROLOGIQUE LUCIEN NEUWIRTH	SAINT PRIEST EN JAREZ (FRANCE)	Investigator	
Asmahane	BENMAZIANE-TEILLET		DR	CH FOCH	SURESNES (FRANCE)	Investigator	
Thierry	LECOMTE		PR	CHU TROUSSEAU	TOURS (FRANCE)	Investigator	
