# Supplementary material for: FOLFIRI Plus Durvalumab With or Without Tremelimumab in Second-Line Treatment of Advanced Gastric or Gastroesophageal Junction Adenocarcinoma: The PRODIGE 59-FFCD 1707-DURIGAST Randomized Clinical Trial
Source: JAMA Oncol. 2024 Apr 4;10(6):709–17. doi: 10.1001/jamaoncol.2024.0207 (PMC11190792; doi:10.1001/jamaoncol.2024.0207)
Supplement: Supplement 4. — The PRODIGE 59-FFCD 1707-DURIGAST investigators/collaborators [file jamaoncol-e240207-s004.pdf]

Supplemental Online Content

Tougeron D, Dahan L, Evesque L, et al. FOLFIRI plus durvalumab with or without tremelimumab in second-line treatment of advanced gastric or gastroesophageal junction adenocarcinoma: the PRODIGE 59-FFCD 1707-DURIGAST Randomized Clinical Trial. *JAMA Oncol*. Published online April 4, 2024. doi:10.1001/jamaoncol.2024.0207

eTable 1. Univariate and multivariate analysis of predictive of factors of progression-free survival
eFigure 1. Progression-free survival according to combined positive score (CPS)
eFigure 2. Progression-free survival according to tumor proportion score (TPS)
eFigure 3. Time to deterioration (10 points) in global quality of life score

This supplemental material has been provided by the authors to give readers additional information about their work.

© 2024 Tougeron D, et al. *JAMA Oncol.*

eTable 1. Univariate and multivariate analysis of predictive of factors of progression-free survival

		Events / n (%)	Univariate analysis Hazard ratio [95%CI] p-value	Multivariate analysis Hazard ratio [95%CI] p-value
**Arm**	Folfiri + durvalumab	44/47 (93.6)	Reference	
	Folfiri + durvalumab + tremelimumab	41/45 (91.1)	0.84 [0.54;1.29]; p=0.429	
**Age (years)**	≥ 60 years	41/45 (91.1)	Reference	
	< 60 years	44/47 (93.6)	1.22 [0.8;1.88]; p=0.356	
**Sex**	Female	26/28 (92.9)	Reference	
	Male	59/64 (92.2)	1.14 [0.72;1.82]; p=0.573	
**ECOG PS**	1	57/61 (93.4)	Reference	
	0	28/31 (90.3)	0.87 [0.55;1.39]; p=0.570	
**Liver metastases**	Yes	36/37 (97.3)	Reference	Reference
	No	49/55 (89.1)	0.47 [0.3;0.74]; p=0.001	0.46 [0.29;0.74]; p=0.001
**Body Mass Index (kg/m^2)**	> 25 kg/m²	52/59 (88.1)	Reference	Reference
	≤ 25 kg/m²	33/33 (100)	1.50 [0.97;2.33]; p=0.071	1.29 [0.82;2.02]; p=0.273
**Time to metastatic disease**	Synchronous	56/60 (93.3)	Reference	
	Metachronous	29/32 (90.6)	0.73 [0.46;1.14]; p=0.168	
**HER2 status**	Positive	18/21 (85.7)	Reference	Reference
	Negative	66/70 (94.3)	1.54 [0.91;2.61]; p=0.106	1.59 [0.94;2.71]; p=0.086
**Primary tumour site**	Stomach	40/43 (93.0)	Reference	
	Gastro-oesophageal junction	45/49 (91.8)	0.76 [0.49;1.16]; p=0.200	
**Tumour subtype (Lauren classification)**	Diffuse type	35/37 (94.6)	Reference	
	Intestinal type	44/48 (91.7)	0.75 [0.48;1.18]; p=0.216	
	Unknown	5/6 (83.3)	0.55 [0.22;1.41]; p=0.214	
**Peritoneal carcinomatosis**	Yes	31/33 (93.9)	Reference	
	No	54/59 (91.5)	0.91 [0.59;1.42]; p=0.692	
**Previous Treatment**	Doublet	53/56 (94.6)	Reference	
	Single agent	2/2 (100)	1.04 [0.25;4.32]; p=0.954	
	Triplet	30/34 (88.2)	0.79 [0.5;1.25]; p=0.310	

ECOG PS: Eastern Cooperative Oncology Group performance status

© 2024 Tougeron D, et al. *JAMA Oncol.*

eFigure 1. Progression-free survival according to combined positive score (CPS)

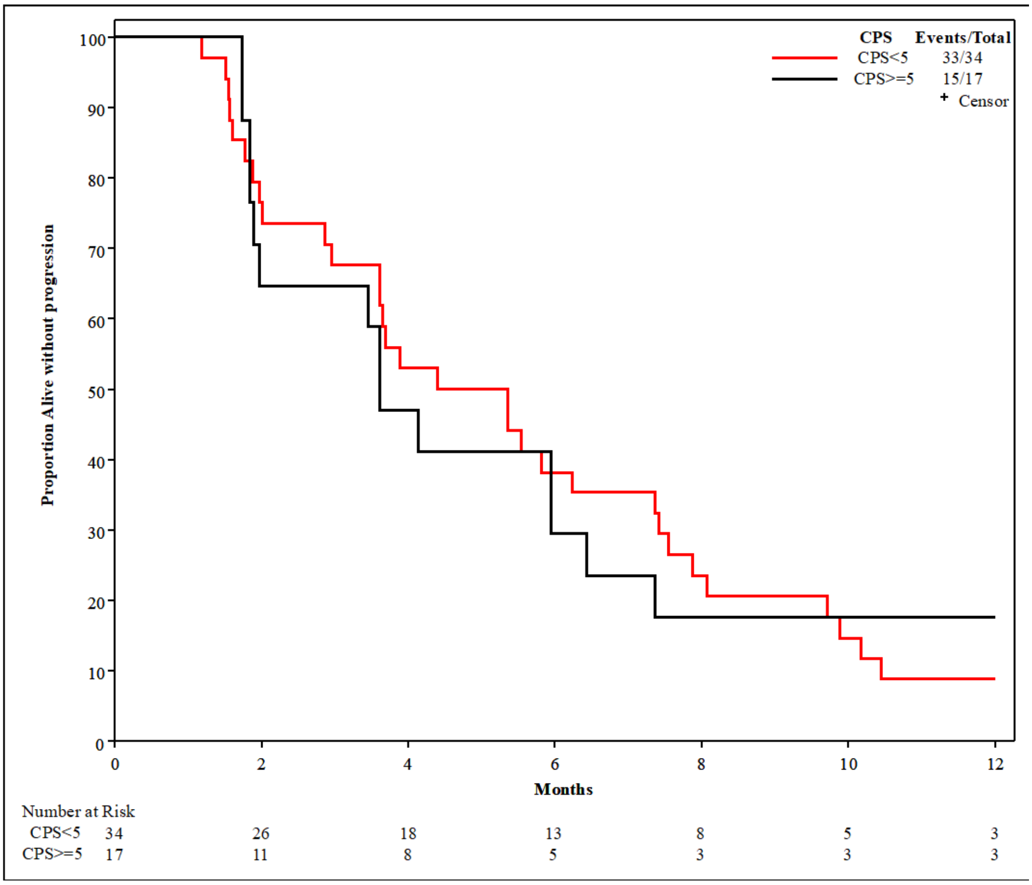

© 2024 Tougeron D, et al. *JAMA Oncol.*

eFigure 2. Progression-free survival according to tumour proportion score (TPS)

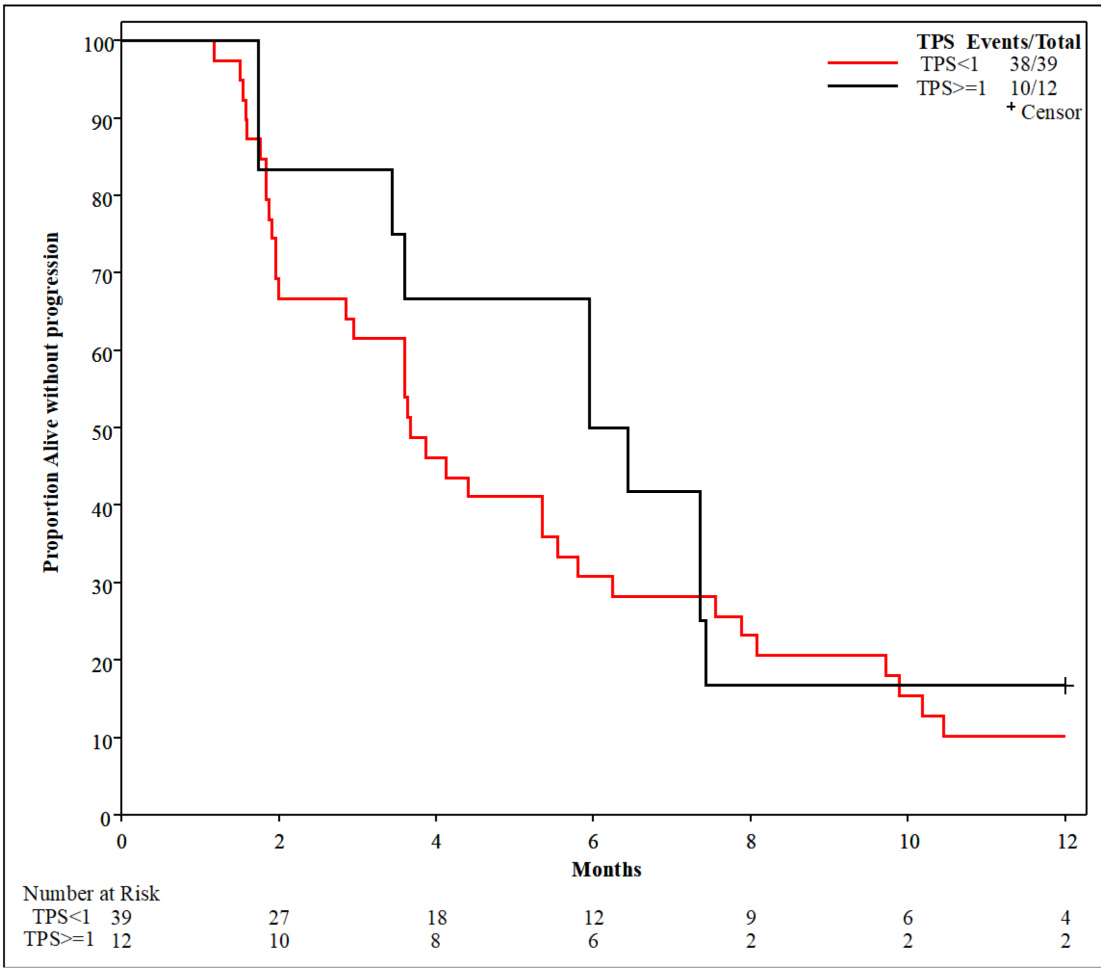

© 2024 Tougeron D, et al. *JAMA Oncol.*

eFigure 3. Time to deterioration (10 points) in global quality of life score

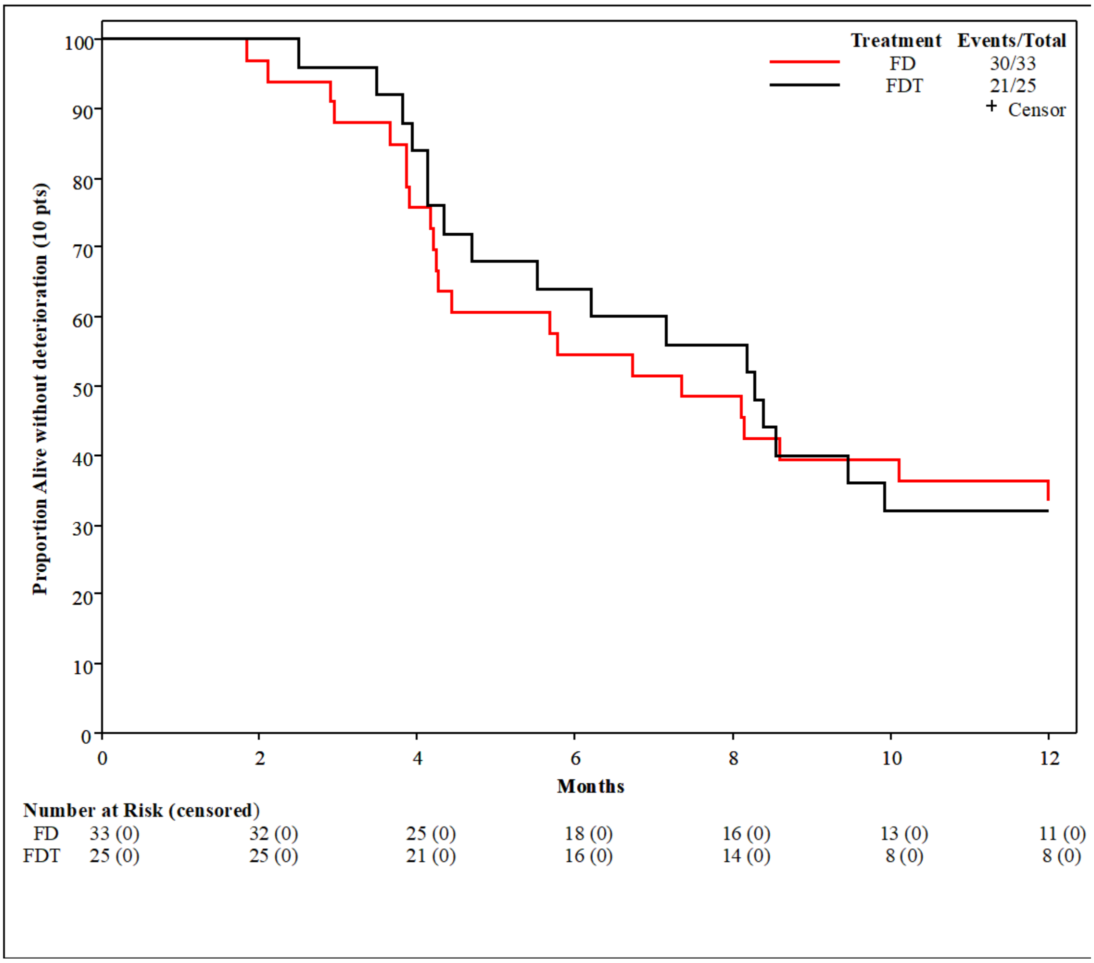

© 2024 Tougeron D, et al. *JAMA Oncol.*
