# Supplementary material for: FOLFIRI Plus Durvalumab With or Without Tremelimumab in Second-Line Treatment of Advanced Gastric or Gastroesophageal Junction Adenocarcinoma: The PRODIGE 59-FFCD 1707-DURIGAST Randomized Clinical Trial
Source: JAMA Oncol. 2024 Apr 4;10(6):709–17. doi: 10.1001/jamaoncol.2024.0207 (PMC11190792; doi:10.1001/jamaoncol.2024.0207)
Supplement: Supplement 5. — Data Sharing Statement [file jamaoncol-e240207-s005.pdf]

Data Sharing Statement

Data
Data available: No

Additional Information
Explanation for why data not available: The PRODIGE 59-FFCD 1707-DURIGAST data base is open to the scientific and medical community upon request to the steering committee. All data will be made available (de-identified participant data, participant data with identifiers, data dictionary, or other specified data set) depending on the collaboration in place. Study protocol, statistical analysis plan, informed consent form, consortium status are available upon request. Proposals should be addressed to Mrs Cecile Girault, project manager of the FFCD at: cecile.girault@ubourgogne.fr. Cecile Girault will then prepare the dossiers for the steering committee. The steering committee FFCD will evaluate the pertinence of the request before sending the database to any academic partners. After agreement of this Steering Committee, data requestors will have to sign a data access agreement to gain access to the database. FFCD as the sponsor will be vigilant regarding the General Data Protection Regulation (GDPR) compliance of the requestors.
